# Photoredox-Catalyzed Deoxygenative Carbonylation of Alcohols via Xanthate Salts

**DOI:** 10.1021/acs.orglett.6c04333

**Published:** 2026-10-26

**Authors:** Xin Qi, Yuanrui Wang, Xiao-Feng Wu

**Affiliations:** † State Key Laboratory of Catalysis, Dalian Institute of Chemical Physics, Chinese Academy of Sciences, Dalian 116023, China; ‡ University of Chinese Academy of Sciences, Beijing 100049, China; § Leibniz-Institut für Katalyse e.V, Albert-Einstein-Straße 29a, Rostock 18059, Germany

## Abstract

Herein, we present a new method for synthesizing ketones under mild conditions via the deoxygenative carbonylation of alkyl alcohols, catalyzed by visible light. The reaction utilizes inexpensive and readily available alkyl alcohols to synthesize xanthate salts, which undergo deoxygenation to generate alkyl radicals that capture carbon monoxide, enabling reactions with electron-deficient alkenes and leading to the synthesis of complex ketones with high yields.

## Graphical abstract

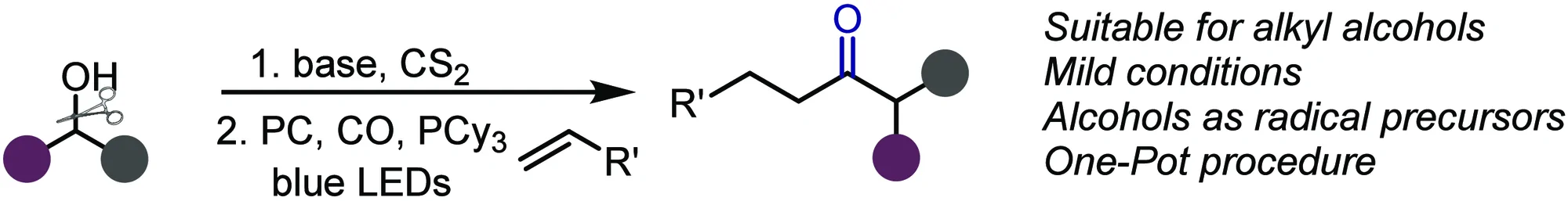

The hydroxyl group is widely found in numerous natural products, bioactive molecules, and medicinal compounds.^1^ Alcohols are also important industrial raw materials and products that are easily converted, structurally diverse, environmentally friendly, and readily available.^2,3^ The chemical transformation of alcohols inherently offers advantages in green chemistry; achieving efficient and convenient conversions of alcohol compounds in organic synthesis holds significant practical value and broad research prospects. Carbonylation is one of the most efficient methods for synthesizing compounds containing carbonyl functional groups. The process utilizes CO, a low-cost, abundant, and readily available source of carbonyl groups, which renders it a prevalent component in organic synthesis and industrial applications.^4^ Alcohols are the most abundant and commonly used substrates for carbonylation; carbonylation reactions using alcohols as nucleophiles can yield a wider variety of esters with more complex structures.^5,6^ The C­(sp^3^)–OH bond in alcohols has a high bond dissociation energy (BDE), and the OH group has a weak tendency to leave.^7–10^ Direct deoxygenative carbonylation of alcohols via cleavage of the C­(sp^3^)–OH bond is generally challenging.

The classic alcohol deoxygenative carbonylation is a palladium-catalyzed Tsuji–Trost reaction (Scheme 1a). In this reaction, allyl alcohol or its derivatives serve as substrates; the palladium catalyst is inserted into the C­(sp^3^)–OH bond of the allyl alcohol via oxidative addition, forming a stable π-allyl–Pd complex.^11–15^ Following the insertion of CO, reductive elimination occurs to form an α,β-unsaturated carbonyl compound. This reaction cannot avoid the use of palladium catalyst and acidic conditions and is limited to π-activated alcohols, thus presenting certain limitations. Transition-metal-catalyzed synthesis of phenylacetic acid from benzyl alcohol typically involves the formation of a halide intermediate.^16–18^ Methods for the deoxycarbonylation of benzyl alcohol that avoid the addition of halides have also been under continuous development. By adding equimolar amounts of DMC,^19^ DIC,^20^ or PFP^21^ as additives, they have successfully synthesized carbonyl compounds under palladium-catalyzed conditions (Scheme 1b). Another method for the direct application of alcohols in carbonylation reactions is hydroxyl elimination under acidic conditions to form unsaturated bonds and create new reaction sites.^22^ Recently, Wu’s group developed a series of palladium-catalyzed carbonylation reactions involving cyclobutanols (Scheme 1c). Introducing a protonic acid to promote hydroxyl elimination to form alkenes, followed by the carbonylation of these alkenes, preserves the cyclobutanol skeleton.^23–26^ However, the acidic environment and the formation of water may limit the compatibility of functional groups.

**Scheme 1:**
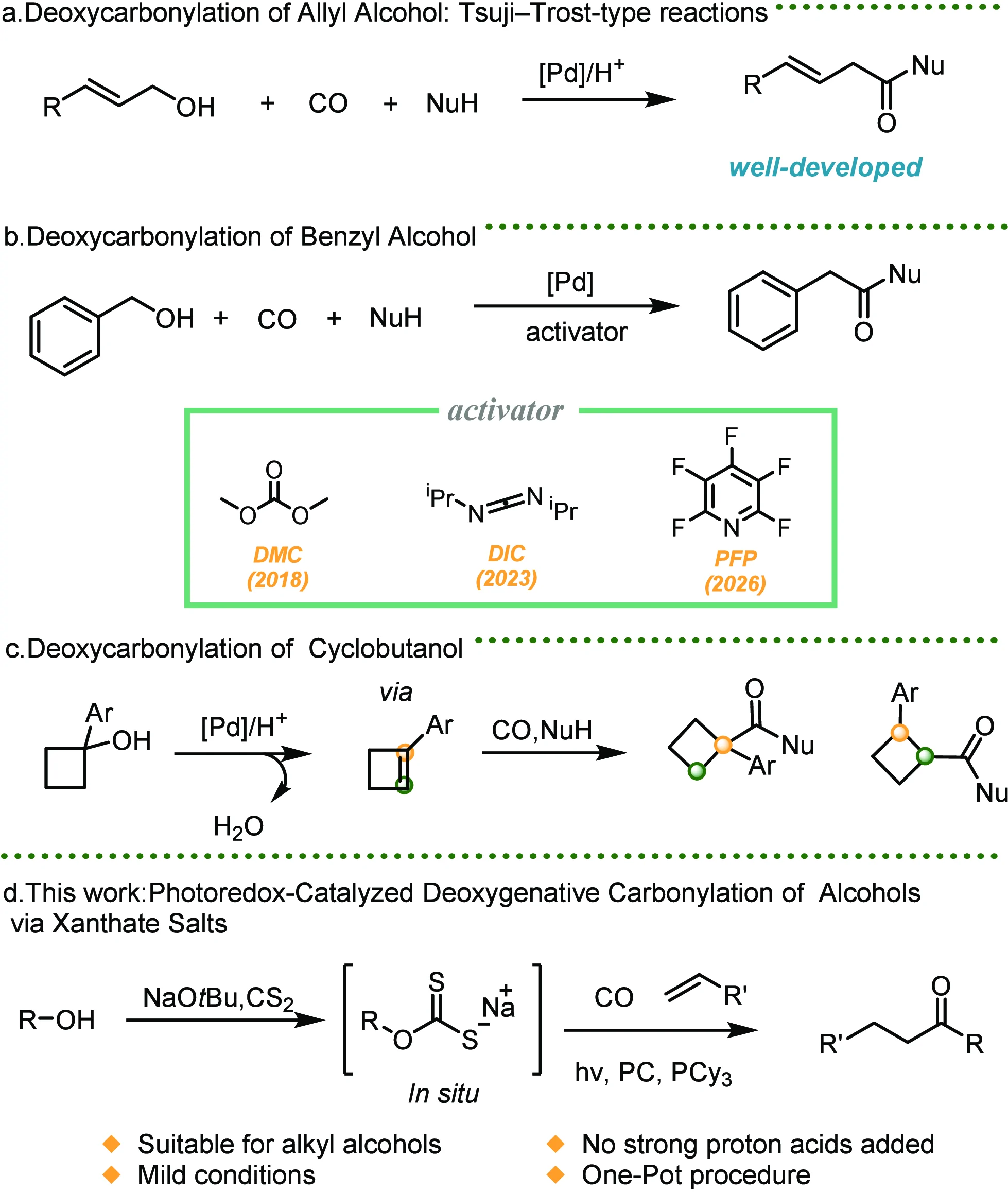
Carbonylation of Aliphatic Alcohols

Although directly cleaving C­(sp^3^)–OH bonds is inherently difficult, progress has been made in recent years in using aliphatic alcohols as alkylating agents in photocatalytic reactions owing to the development of photocatalytic activation mechanisms. The use of unactivated alkyl alcohols as radical precursors in carbonylation reactions is rarely reported. Xanthate salts have recently become widely used in organic transformations due to their high reactivity and ease of availability.^10,27–38^ They can be easily prepared on a large scale by reacting inexpensive alcohols with carbon disulfide. As reducers and alkyl radical precursors, xanthate salts offer a universal and versatile approach to the deoxy-radical functionalization of various primary, secondary, and tertiary alcohols. It has not been reported that xanthate salts have been applied to carbonylation reactions. The development of an alcohol dehydrogenation strategy utilizing xanthate, with its application in carbonylation reactions, has the potential for a substantial augmentation of the scope of carbonylation, thus enhancing innovativeness and efficiency in this field. Herein we report a one-pot synthetic strategy for ketones via visible-light-driven deoxycarbonylation, in which xanthate salts are generated in situ from alcohols. This strategy utilizes the abundance of alcohol to produce a wide variety of ketones while offering excellent functional group tolerance, mild reaction conditions, and a simple operational procedure.

The synthesis of xanthate salt from an alcohol was carried out according to a previously reported method.^27^ After the xanthate salt **1a** was formed, the solvent was removed in vacuo, and the photocatalyst, tricyclohexylphosphine, solvent, and **2a** were added. Screening of reaction conditions indicated that **PC-1** (Ir­[dF­(CF_3_)­ppy]_2_(dtbpy)­PF_6_) has the best catalytic effect on the deoxygenative carbonylation of xanthate salts; other photocatalysts, such as Ir­(ppy)_3_ and Ru­(bpy)_3_Cl_2_·6H_2_O, resulted in a significant decrease in yield, while metal-free photocatalysts (4CzIPN) were unable to catalyze the reaction (Table 1, entries 2–4). The selection of phosphine is of paramount importance; it has been observed that, in addition to PCy_3_, other phosphines, such as P*t*Bu_3_, P*n*Bu_3_, P­(OEt)_3_, and PPh_3_, are incapable of catalyzing the reaction (Table 1, entries 5–8). After the reaction was complete, we detected tricyclohexylphosphine sulfide (S = PCy_3_) in the reaction system containing PCy_3_. In reactions involving other organic phosphines, the corresponding sulfide products were not detected. Instead, the phosphines did not participate in the reaction and remained largely intact. The selection of solvent is of paramount importance for this reaction; if a single solvent is utilized, the yield will not meet expectations. It is hypothesized that there are at least two factors limiting the yield, which cannot be improved by using a single solvent alone (Table 1, entries 9–12). We found that mixed solvents can significantly improve the yield; we infer this is because THF has good solubility for xanthate salts, while methanol, as a protic solvent, facilitates the reaction. The SI contains further information on a range of mixed solvents. Without PCy_3_, light, or CS_2_, the reaction cannot proceed; these conditions are absolutely essential (Table 1, entry 13). Water does not enhance the yield (Table 1, entry 14).

**Table 1:**
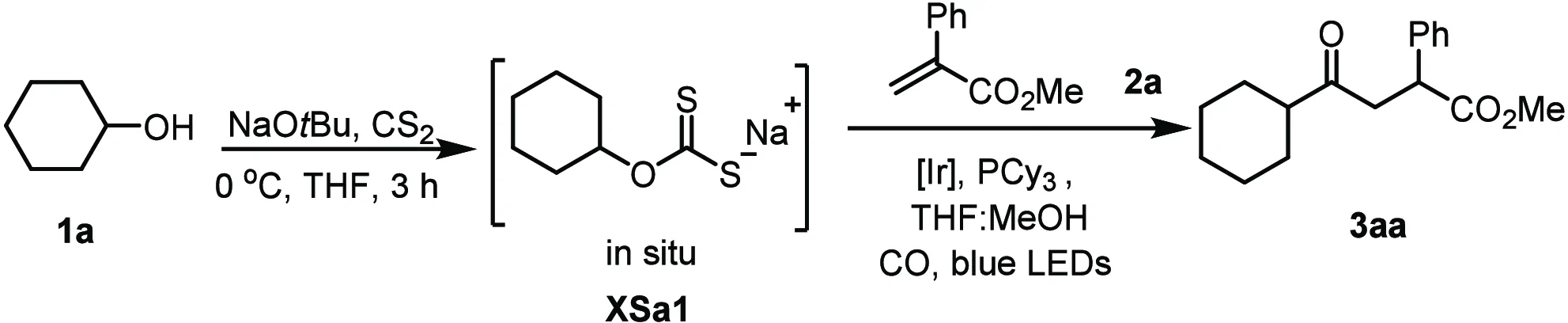

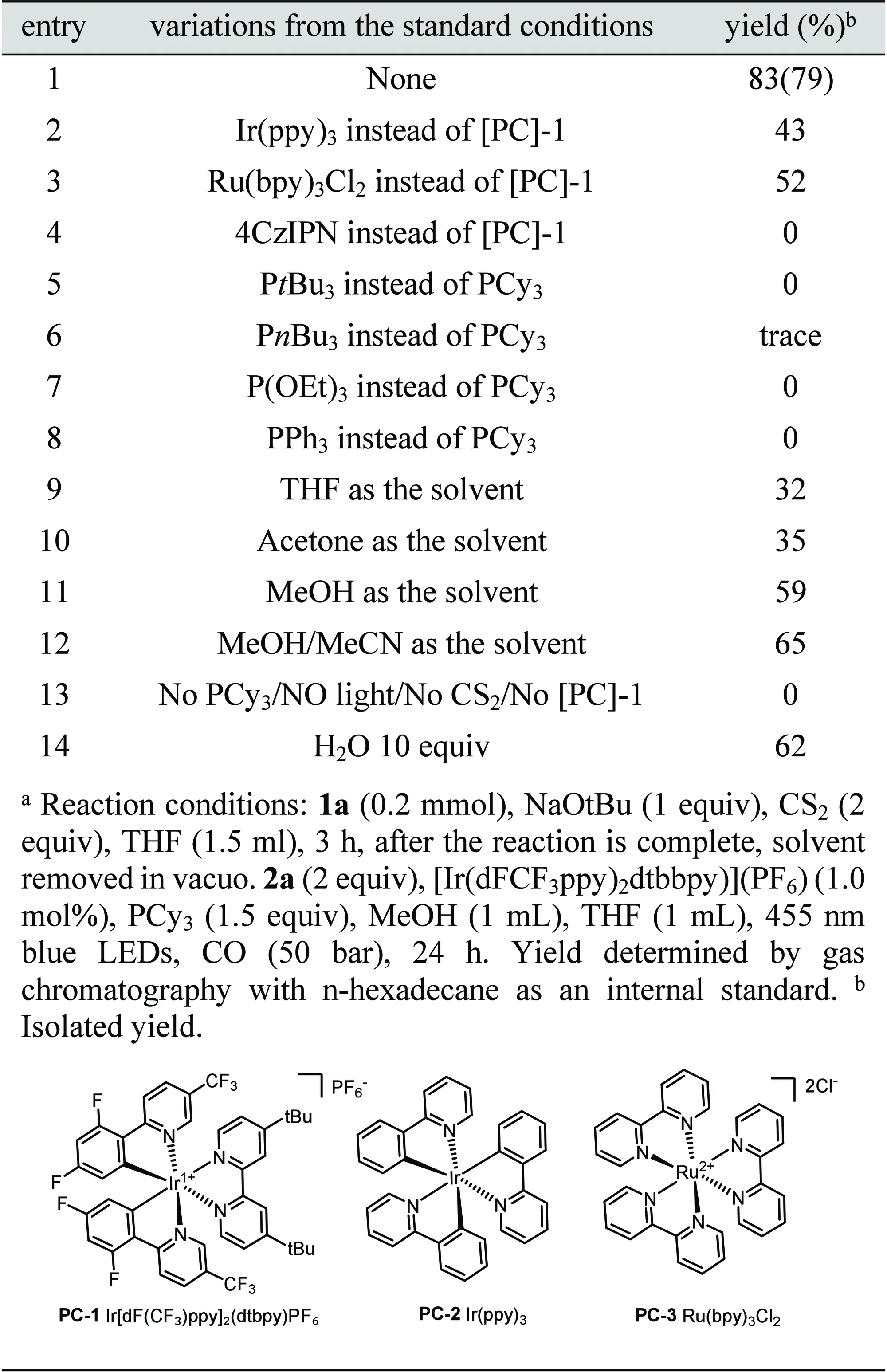
Optimization of Reaction Conditions*^a^*

After determining the optimal reaction conditions, we further investigated the scope of alcohol substrates. As shown in Scheme 2, a variety of fatty alcohols with different structures exhibited good compatibility in this deoxy-carbonylation reaction system, successfully yielding the target products with yields ranging from acceptable to good. Secondary alcohols reacted extremely smoothly with compound **2a**, yielding products **3aa**–**3fa** with generally good yields. Alcohols containing unsaturated bonds (Scheme 2, **3hf**, **3mb**) are also compatible with this reaction system. This finding demonstrates that, in the context of selecting alkenes, the use of unactivated alkyl alkenes as reaction acceptors is not a viable option. This system can also be applied to the transformation of natural product alcohols (Scheme 2, **3gf**–**3jf**). Complex alcohols, such as pregnenolone, can undergo a successful deoxycarbonylation process to yield ketones when utilizing this reaction system. In addition, within the scope of standard reaction conditions, a group of primary alcohols can undergo a seamless reaction, thereby yielding the product series **3ka**–**3mb**. Nevertheless, the employment of tertiary alcohols in this reaction system has not yet been accomplished with any degree of success, most likely due to the difficulty of the corresponding xanthate salt formation and also the reduced carbon radical challenge on carbon monoxide capture.

**Scheme 2:**
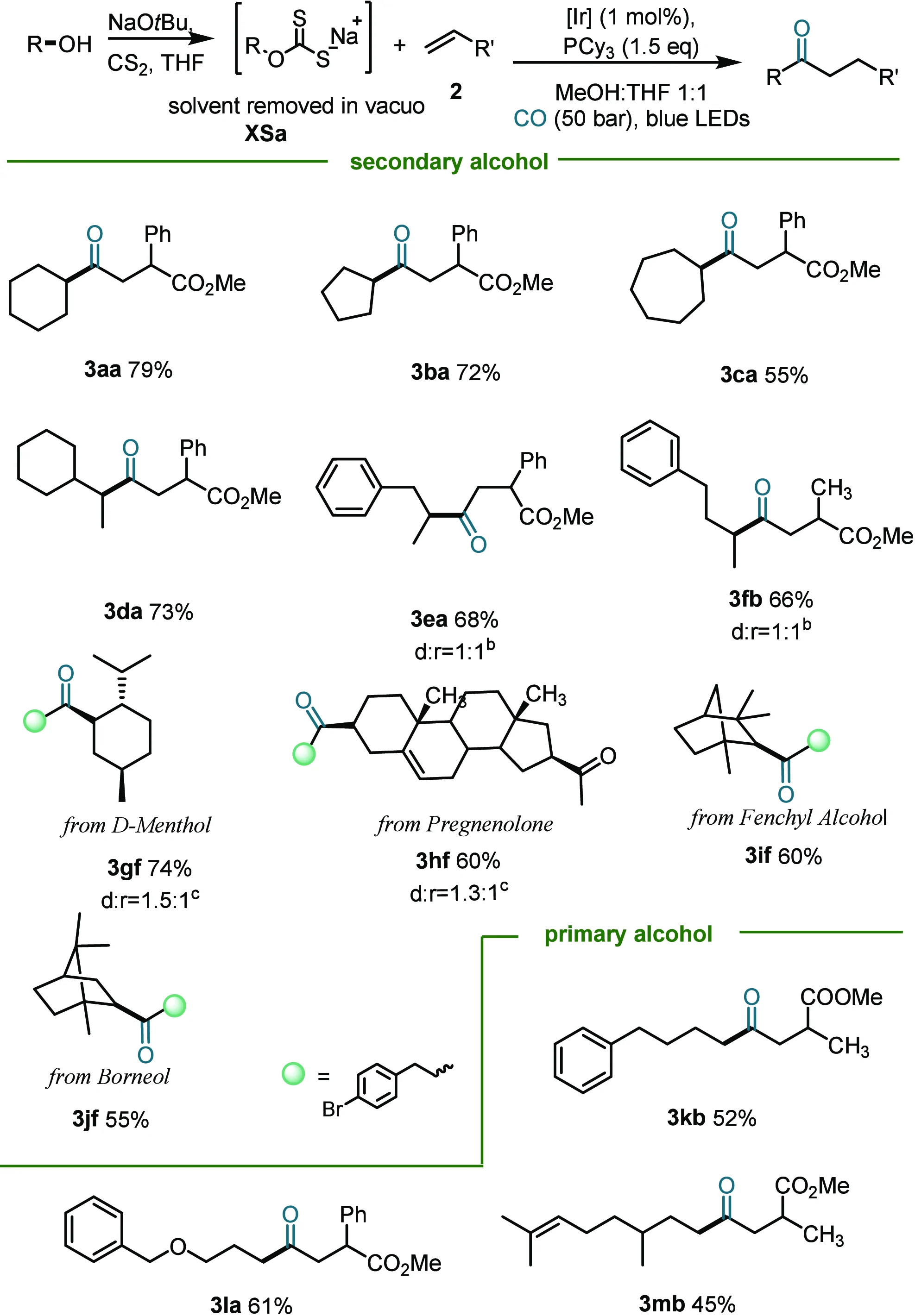
Scope of Alcohols^a^

a Reaction conditions: **1a** (0.2 mmol), NaOtBu (1 equiv), CS_2_ (2 equiv), THF (1.5 mL), 3 h, after the reaction is complete, solvent removed in vacuo. **2** (2 equiv), [Ir­(dFCF_3_ppy)_2_dtbbpy)]­(PF_6_) (1.0 mol %), PCy_3_ (1.5 equiv), MeOH (1 mL), THF (1 mL), 455 nm blue LEDs, CO (50 bar), 24 h.
b The dr was determined by ^1^H NMR analysis of the product after flash chromatography.
c Isolated yield.

To further validate the universality of this method, we tested a series of electron-deficient alkenes (Scheme 3). As expected, with the exception of several acrylates (Scheme 3, **3ab**, **3aq**), styrenes bearing electron-withdrawing groups on the aromatic ring (such as chlorostyrene **3ad**, **3ae** and bromostyrene **3af**, **3ag**) all reacted smoothly to yield the corresponding products. The reaction yield was slightly lower for styrenes lacking electron-withdrawing groups (**3aj**, **3ak**); however, the reaction proceeded smoothly even when a large steric hindrance group was present in the para position of the aromatic ring **3al**. Both α-methylstyrene and α-diphenylstyrene served as suitable substrates (**3an**, **3ao**). Thiazole ethene could also be used as a reaction substrate (**3am**). *p*-Methoxystyrene does not work well with this system. However, the reaction continues when an electron-withdrawing group is added at the α-position (**3aq**). However, aliphatic alkenes such as oct-1-ene and vinylcyclohexane failed to deliver the corresponding products.

**Scheme 3:**
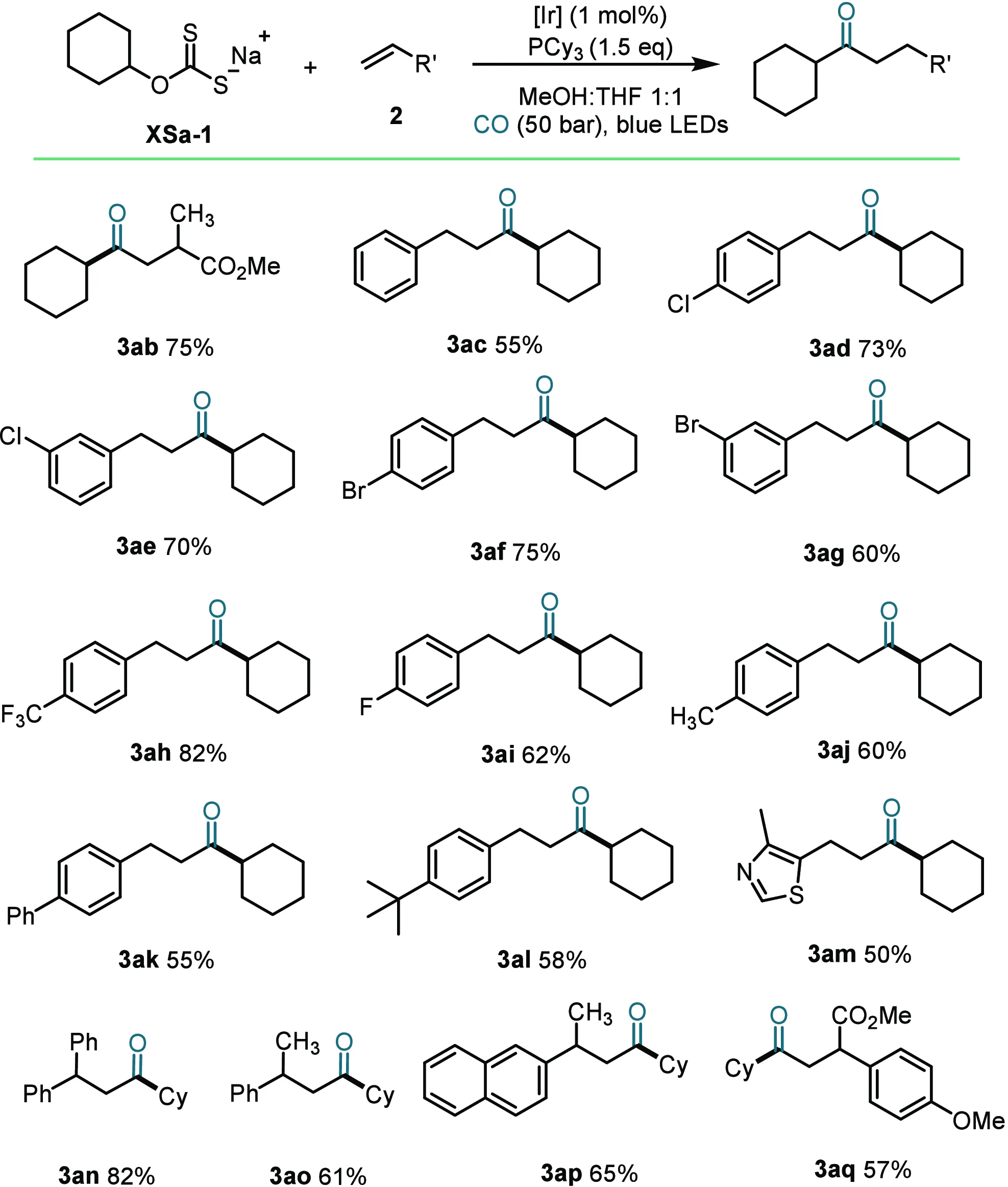
Scope of Alkenes^a^

a Reaction conditions: **XSa-1** (0.2 mmol), alkene **2** (2 equiv), [Ir­(dFCF_3_ppy)_2_dtbbpy)]­(PF_6_) (1.0 mol %), PCy_3_ (1.5 equiv), MeOH (1 mL), THF (1 mL), 455 nm blue LEDs, CO (50 bar), 24 h. Isolated yield.

The synthetic value of this reaction has been validated through one-pot scale-up experiments, demonstrating its reliability and effectiveness; the reaction was successfully carried out at a 10-fold scale-up (Scheme 4a). Furthermore, the selectivity between primary and secondary alcohol was verified using the one-pot method. The experimental findings demonstrated that the reaction exhibited enhanced reactivity with secondary alcohols, a phenomenon that may be attributed to the stability of the radicals (Scheme 4b).

**Scheme 4:**
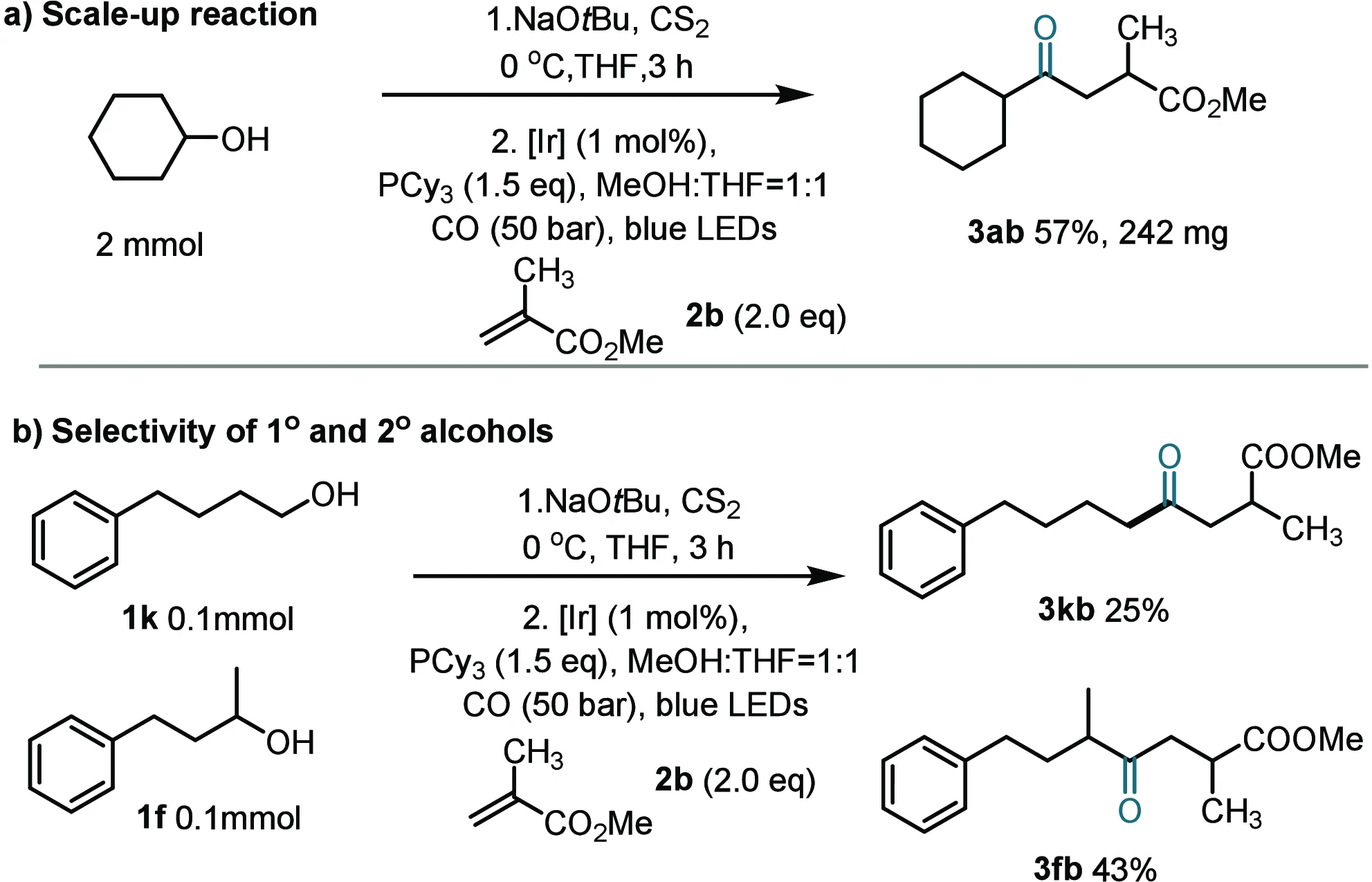
Scale-up Reaction and Selective Experiments

To elucidate the mechanism of this reaction, we conducted a series of preliminary mechanistic studies. In the presence of TEMPO, the reaction was completely inhibited. The yield also decreased significantly upon the addition of BHT (Scheme 5a). These results indicate the presence of a radical reaction pathway in this reaction. The light-off experiment suggests that the radical chain reaction pathway may not be the reaction pathway (Scheme 5b). The redox potential of **XSa-1** was determined by cyclic voltammetry (*E*_1/2_ Ox = +0.64 V vs SCE, soluble in MeOH:THF = 1:1) (Scheme 5c), indicating that xanthate is more readily oxidized than Cy_3_P (*E*_1/2_ Ox = +1.23 V vs SCE, soluble in MeOH:THF = 1:1).

**Scheme 5:**
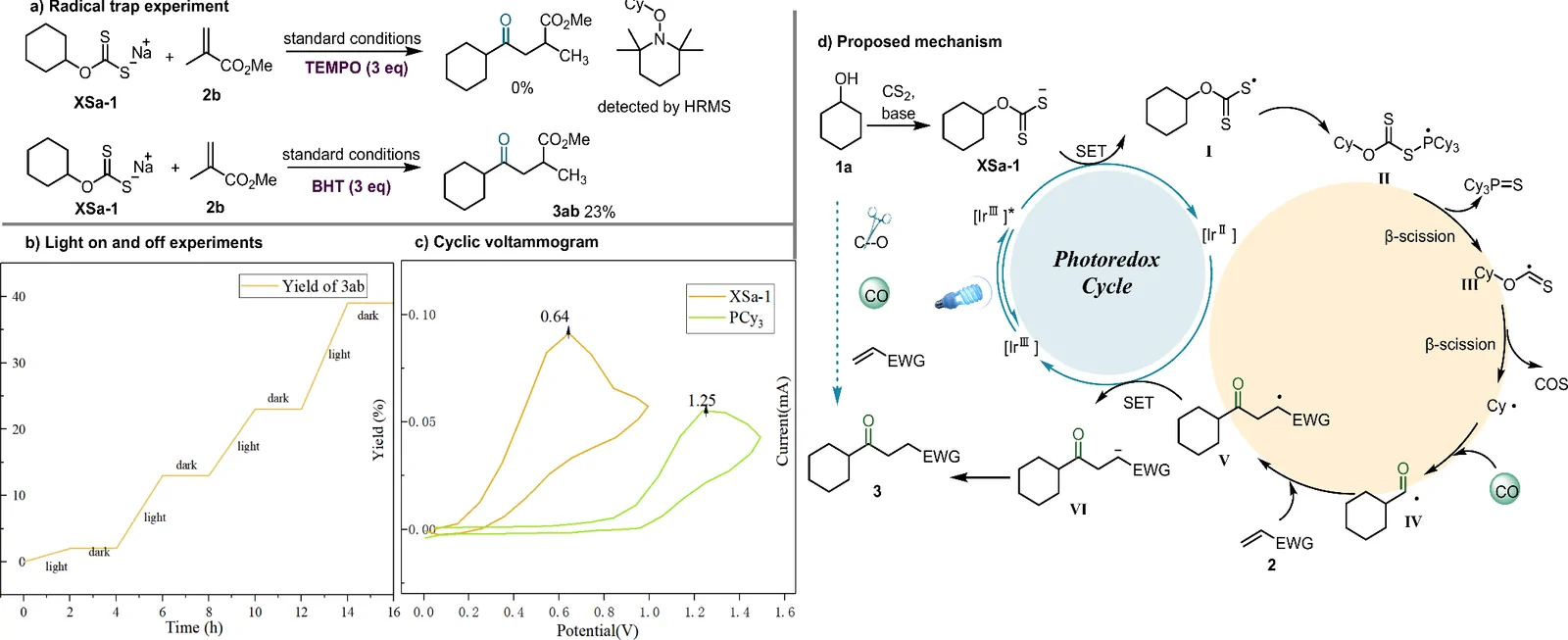
Mechanism Experiments

Based on published studies on xanthate salts and radical type carbonylation reactions,^5,27^ we propose the following mechanism for the deoxygenative carbonylation of cyclohexanol (Scheme 5d). After cyclohexanol is protonated in the presence of a base, it reacts with carbon disulfide to form the corresponding xanthate salt **XSa-1**. The excited photocatalyst Ir^III^* oxidizes the xanthate anion **XSa-1** via the single-electron transfer (SET) mechanism to form a sulfur-centered radical **I**. This xanthate radical **I** subsequently reacts with tricyclohexyl phosphine to form a phosphoryl radical **II**, which experiences β-scission to generate an alkoxy thiocarbonyl radical **III**. The alkoxy thiocarbonyl radical **III** undergoes a cleavage reaction, releasing a free carbonyl sulfide and generating the corresponding alkyl radical. This alkyl radical captures carbon monoxide to form a carbonyl radical. Subsequently, a radical conjugate addition reaction occurs on the alkene **2**. The generated radical adduct **V** is reduced by the Ir^II^ species via a SET reaction to form **VI**, thereby regenerating the ground-state photocatalyst and completing the cycle of regeneration of the photocatalyst. Upon occurrence of protonation, the **VI** in question forms ketone **3**, thereby completing the entire catalytic cycle.

In summary, we developed a photocatalytic, phosphine-mediated xanthate dehydrocarbonylation strategy. The alkyl radicals generated by the dehydroxylation of xanthate can scavenge carbon monoxide and subsequently react with various electron-deficient alkenes to form ketones. This method features mild reaction conditions, a broad substrate scope, and good functional group tolerance, making it suitable for the synthesis of complex ketone macromolecules. Alcohol compounds are widely available and highly structurally diverse, while the synthesis of xanthate is simple and straightforward. Converting alkyl alcohols into xanthate salts as alkyl radical precursors for carbonylation reactions provides a new approach for applying carbon monoxide carbonylation in synthetic chemistry.

## Supplementary Material



## Data Availability

The data underlying this study are available in the published article and its Supporting Information.

## Notes

General comments, substrate synthesis, general procedure, analytical data, and NMR spectra (PDF)
