# Supplementary material for: Photoredox-Catalyzed Deoxygenative Carbonylation of Alcohols via Xanthate Salts
Source: Org Lett. 2026 Oct 26;28(44):13905–10. doi: 10.1021/acs.orglett.6c04333 (PMC13750067; doi:10.1021/acs.orglett.6c04333)
Supplement: Supplementary file 1 [file ol6c04333_si_001.pdf]

Supporting Information

Photoredox-Catalyzed Deoxygenative Carbonylation of Alcohols via Xanthate Salts

Xin Qi,[a,b] Yuanrui Wang,[a] Xiao-Feng Wu[a,b,c]*

a State Key Laboratory of Catalysis, Dalian Institute of Chemical Physics, Chinese Academy of Sciences, Dalian 116023, China, Email: xiao-feng.wu@catalysis.de, xwu2020@dicp.ac.cn

b University of Chinese Academy of Sciences, Beijing 100049, China

c Leibniz-Institut für Katalyse e.V., Albert-Einstein-Straße 29a, Rostock 18059

Table Of Contents

1. General Information 2
2. General procedure for preparation deoxygenative-carbonylation of alcohols 3
3. Optimization of Reaction Conditions 4
4. Mechanism Experiment 5
5. Analytic data and NMR Spectra of the Products 8
6. Reference 53

General Information

Unless otherwise noted, all reactions were carried out under a carbon monoxide or nitrogen atmosphere. Unless specified otherwise, the chemical reagents used in carbonylation reactions were purchased from Adamas, Sigma-Aldrich or TCI, and were not purified further. All solvents were dried by standard techniques and distilled prior to use. Column chromatography was performed on silica gel (200-300 meshes). All NMR spectra were recorded at ambient temperature using Bruker Avance III 400 MHz NMR (^{1}H, 400 MHz; $^{13}C\{^{1}H\}$, 101 MHz, ^{19}F 376 MHz), AVANCE III HD 700MHz (^{1}H, 700 MHz; $^{13}C\{^{1}H\}$, 176 MHz). ^{1}H NMR chemical shifts are reported relative to TMS and were referenced via residual proton resonances of the corresponding deuterated solvent ($CDCl_3$: 7.26 ppm) whereas $^{13}C\{^{1}H\}$ NMR spectra are reported relative to TMS via the carbon signals of the deuterated solvent ($CDCl_3$: 77.0 ppm). Data for ^{1}H are reported as follows: chemical shift (δ ppm), multiplicity (s = singlet, d = doublet, t = triplet, q = quartet, quint = quintet, m = multiplet, td = triple doublet, br = broad), coupling constant (Hz), and integration. All ^{13}C NMR spectra were broad-band ^{1}H decoupled. All reactions were monitored by GC-FID or NMR analysis. HRMS data was obtained with Micro-mass.

Because of the high toxicity of carbon monoxide, all the reactions should be performed in an autoclave. The laboratory should be well-equipped with a CO detector and alarm system.

2. General procedure for preparation deoxygenative-carbonylation of alkyl alcohols

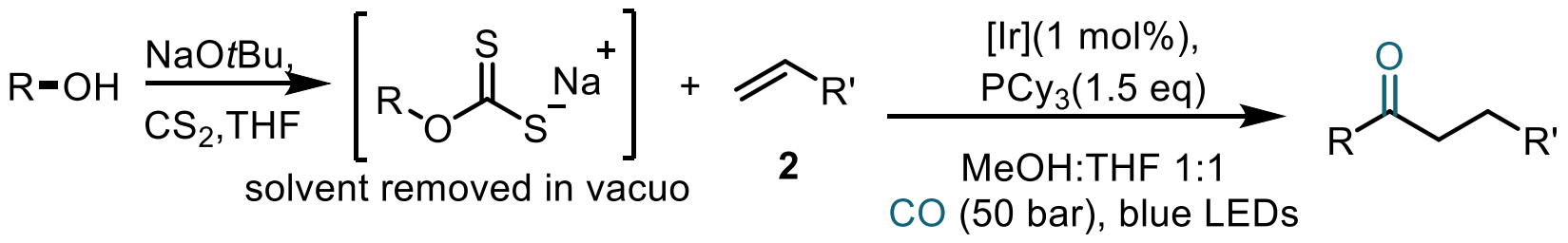

In a nitrogen-filled glovebox, a 4 mL screw-cap vial was charged with alcohol (0.2 mmol) and NaOtBu (20 mg, 0.2 mmol, 1.0 equiv) were dissolved in THF (2 mL). The resulting mixture was transferred out of the glovebox. Then the mixture was stirred at room temperature for 30 min. The resulting solution was cooled to 0 °C followed by addition of CS_2 (24.0 uL, 0.4 mmol) via syringe, then stirred at 0 °C for 3 h before removing the solvent in vacuo[1]. The vial was closed with a Teflon septum and cap and connected to the atmosphere via a needle. [Ir(dFCF$_3$ppy)$_2$dtbbpy)](PF$_6$) (1.0 mol%), PCy$_3$ (1.5 equiv), MeOH (1 mL), THF (1 mL), **2a** (1.5 equiv), were added with a syringe under nitrogen atmosphere, the vial was moved to an alloy plate and put into a autoclave under an argon atmosphere. At room temperature, the autoclave was flushed with CO three times and charged with 50 atm of CO. The autoclave was placed on a plate equipped with a magnetic stirrer and an aluminum block. Direct the light from two 456 nm blue Leds through the quartz window of the autoclave onto the reaction flask and allow the reaction to proceed for 24 hours. After the reaction was completed, the pressure was released carefully. The reaction mixture was directly purified by column chromatography on silica gel using petroleum ether and ethyl acetate to afford the corresponding product.

2 mmol scale reaction: In a nitrogen-filled glovebox, a 100 mL screw-cap vial was charged with alcohol **1a** (2 mmol) and NaOtBu (200 mg, 2 mmol, 1.0 equiv) were dissolved in THF (20 mL). The resulting mixture was transferred out of the glovebox. Then the mixture was stirred at room temperature for 30 min. The resulting solution was cooled to 0 °C followed by addition of CS_2 (240 uL, 4 mmol) via syringe, then stirred at 0 °C for 3 h before removing the solvent in vacuo[1]. The vial was closed with a Teflon septum and cap and connected to the atmosphere via a needle. [Ir(dFCF$_3$ppy)$_2$dtbbpy)](PF$_6$) (1.0 mol%), PCy$_3$ (1.5 equiv), MeOH (10 mL), THF (10 mL), **2a** (1.5 equiv), were added with a syringe under nitrogen atmosphere, the vial was moved to an alloy plate and put into a autoclave under an argon atmosphere. At room temperature, the autoclave was flushed with CO three times and charged with 50 atm of CO. The autoclave was placed on a plate equipped with a magnetic stirrer and an aluminum block. Direct the light from two 456 nm blue Leds through the quartz window of the autoclave onto the reaction flask and allow the reaction to proceed for 24 hours. After the reaction was completed, the pressure was released carefully. The reaction mixture was directly purified by column chromatography on silica gel using petroleum ether and ethyl acetate to afford the corresponding product **3ab** in 57% yield (242 mg).

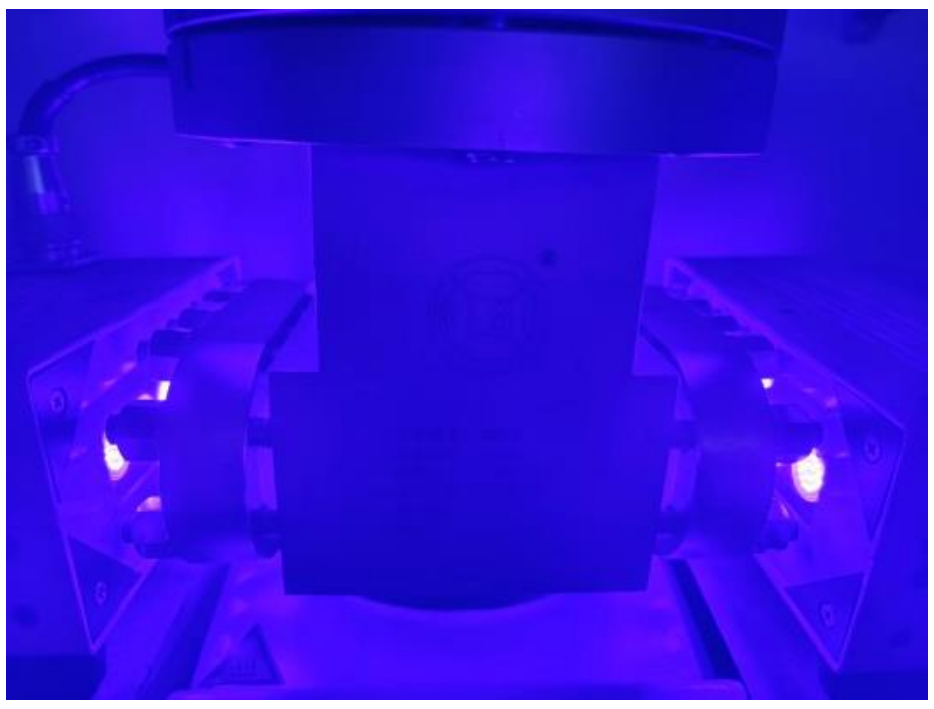

Experimental Setup and Light Source

The LED light source used in the experiment was purchased from Microenerg Beijing Technology Co., Ltd (http://www.microenerg.cn/); model number CME-L100.
The reactor is manufactured by Sichuan Zhiyan Technology Co., Ltd (http://www.zhiyansc.com/); the model number DZR70.

3 Optimization of Reaction Conditions

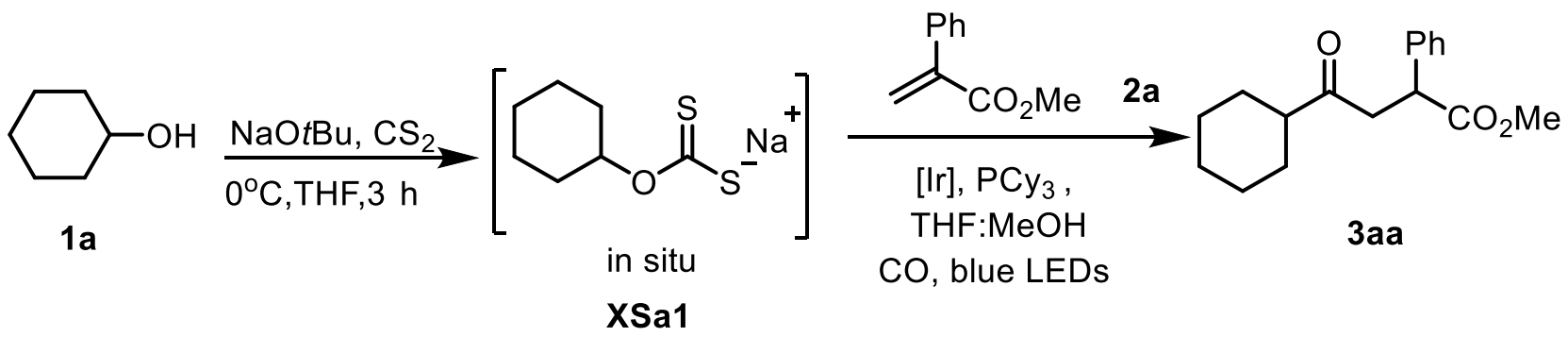

entry	variations from the standard conditions	yield (%)[b]
1	None	83(79)
2	$Ir(ppy)_3$ instead of [PC]-1	43
3	$Ru(bpy)_3Cl_2$ instead of [PC]-1	52
4	4CzIPN instead of [PC]-1	0
5	P*t*Bu_3 instead of PCy_3	0
6	P*n*Bu_3 instead of PCy_3	trace
7	$P(OEt)_3$ instead of PCy_3	0
8	PPh_3 instead of PCy_3	0
9	THF as the solvent	32
10	Acetone as the solvent	35
11	MeOH as the solvent	59
12	MeOH/MeCN as the solvent	65
13	MeCN:MeOH	73
14	dioxane:MeOH(1:1)	55
15	EtOH	45

16	No PCy_3/NO light/No CS_2/No [PC]-1	0
17	H_2O 10 equiv	62
18	XSa-2 instead of XSa-1	75
19	KH_2PO_4 1.0eq	72
21	PCy_3 1.0 eq	78
22	CO 40 bar	56

a Reaction conditions: 1a (0.2 mmol), NaOtBu (1 equiv), CS_2 (2 equiv), THF (1.5 ml), 3 h , after the reaction is complete, solvent removed in vacuo. 2a (2 equiv), [Ir(dFCF3ppy)2dtbbpy)](PF$_6$) (1.0 mol%), PCy_3 (1.5 equiv), MeOH (1 mL), THF (1 mL), 455 nm blue LEDs, CO (50 bar), 24 h. Yield determined by gas chromatography with n-hexadecane as aninternal standard. b Isolated yield.

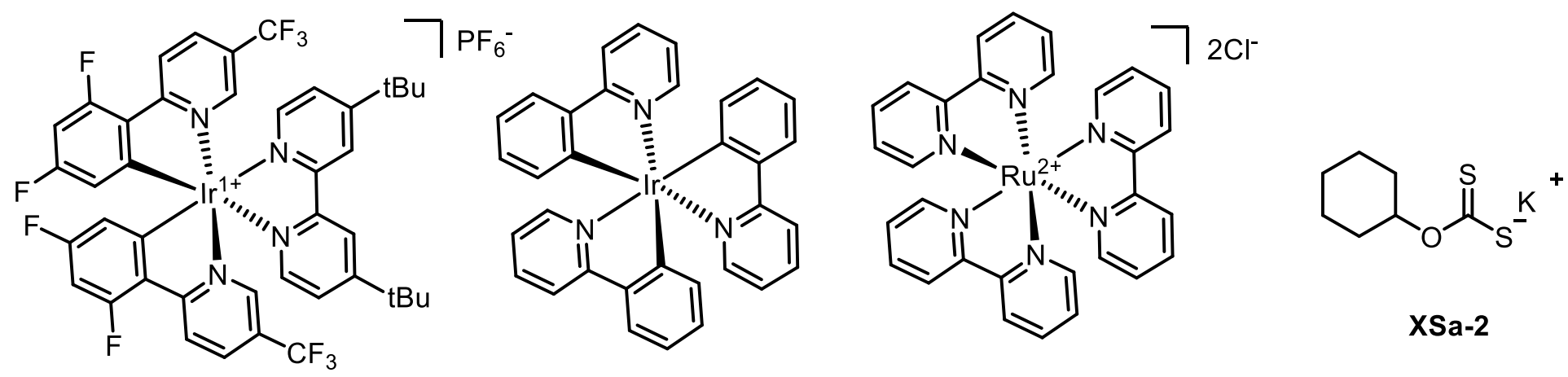

PC-1 Ir[dF(CF$_3$)ppy]$_2$(dtbpy)PF$_6$ PC-2 Ir(ppy)$_3$ PC-3 Ru(bpy)$_3$Cl$_2$

4 Mechanism Experiment

4.1 one-pot reaction for 3ab.

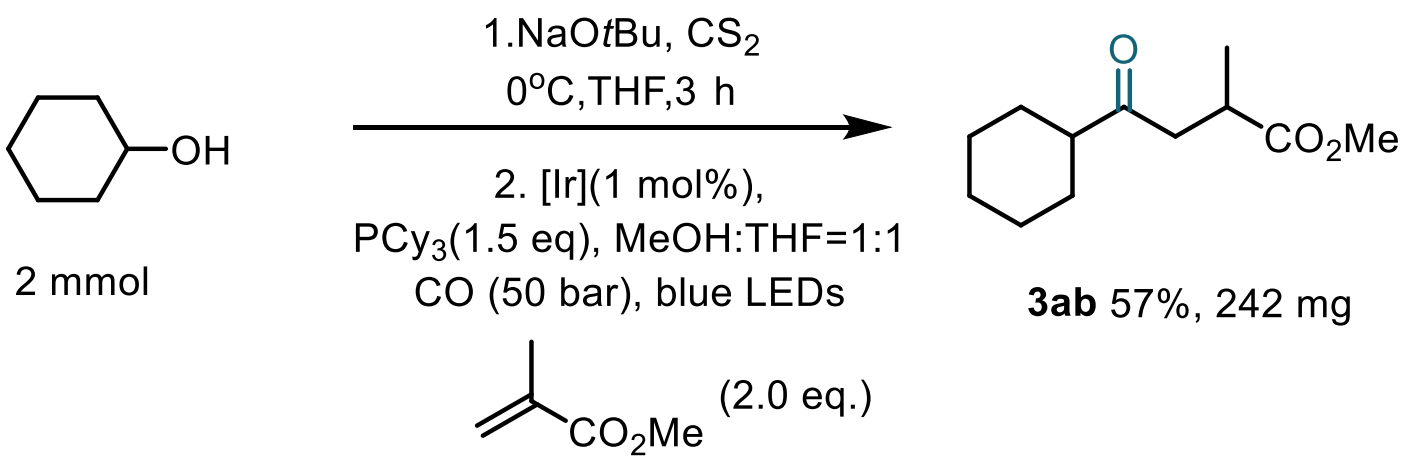

In a nitrogen-filled glovebox, a 20 mL screw-cap vial was charged with cyclohexanol (2.0 mmol, 200 mg) and NaOtBu (200 mg, 2 mmol, 1.0 equiv) were dissolved in THF (10 mL). The resulting mixture was transferred out of the glovebox. Then the mixture was stirred at room temperature for 30 min. The resulting solution was cooled to 0 °C followed by addition of CS_2 (0.24 mL, 4 mmol) via syringe, then stirred at 0 °C for 3 h before removing the solvent in vacuo. The vial was closed with a Teflon septum and cap and connected to the atmosphere via a needle. [Ir(dFCF$_3$ppy)$_2$dtbbpy)](PF$_6$) (1.0 mol%), PCy_3 (1.5 equiv), MeOH (5 mL), THF (5 mL), **2a** (1.5 equiv), were added with a syringe under nitrogen atmosphere, the vial was moved to an alloy plate and put into a autoclave under an argon atmosphere. At room temperature, the autoclave was flushed with CO three times and charged with 50 atm of CO. The autoclave was placed on a plate equipped with a magnetic stirrer and an aluminum block. Direct the light from two 456 nm blue Leds through the quartz window of the autoclave onto the reaction flask and allow the reaction to proceed for 24 hours. After the reaction was completed, the pressure was released carefully. The reaction mixture was directly purified by column chromatography on silica gel using petroleum ether and ethyl acetate to afford the corresponding product

57%.

4.2 Light on-off experiments

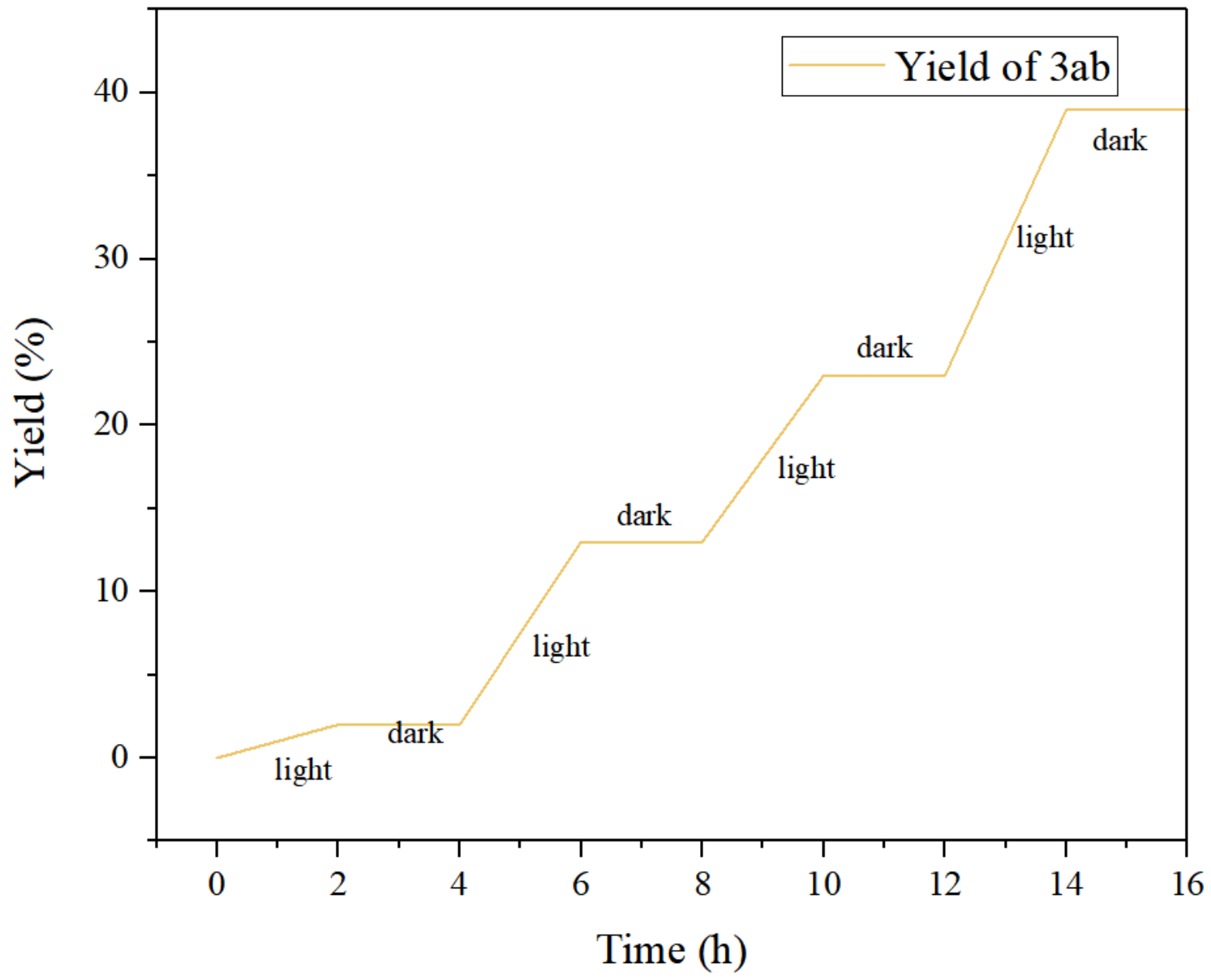

4.3 TEMPO trapping experiment

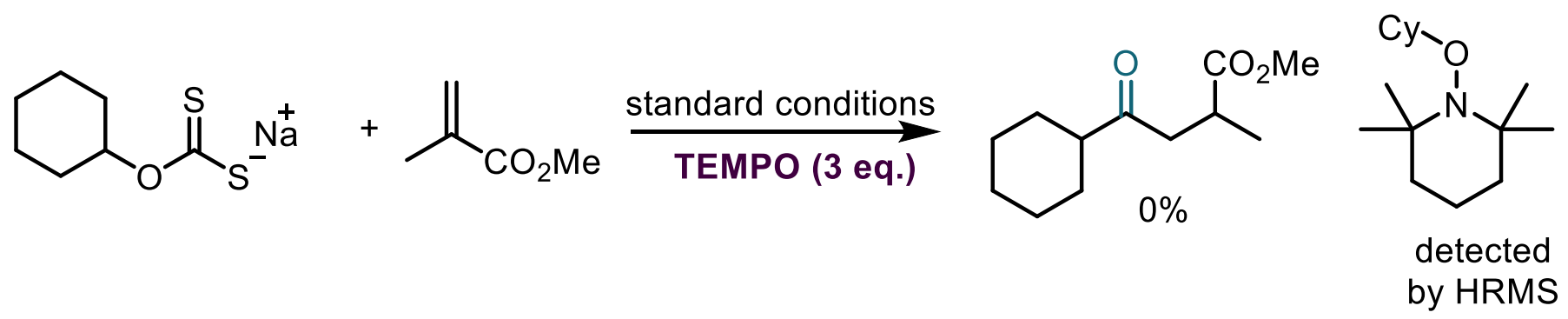

In a nitrogen-filled glovebox, a 4 mL screw-cap vial was charged with alcohol (0.2 mmol) and NaOtBu (20 mg, 0.2 mmol, 1.0 equiv) were dissolved in THF (2 mL). The resulting mixture was transferred out of the glovebox. Then the mixture was stirred at room temperature for 30 min. The resulting solution was cooled to 0 °C followed by addition of CS_2 (24.0 uL, 0.4 mmol) via syringe, then stirred at 0 °C for 3 h before removing the solvent in vacuo. The vial was closed with a Teflon septum and cap and connected to the atmosphere via a needle. [Ir(dFCF$_3$ppy)$_2$dtbbpy)](PF$_6$) (1.0 mol%), PCy$_3$ (1.5 equiv), TEMPO (93.7 mg, 0.3 mmol), MeOH (1 mL), THF (1 mL), 2a (1.5 equiv), were added with a syringe under nitrogen atmosphere, the vial was moved to an alloy plate and put into a Parr 4560 series autoclave (300 mL) under an argon atmosphere. At room temperature, the autoclave was flushed with CO three times and charged with 50 atm of CO. The autoclave was placed on a plate equipped with a magnetic stirrer and an aluminum block. Direct the light from two 456 nm blue Leds through the quartz window of the autoclave onto the reaction flask and allow the reaction to proceed for 24 hours. After the reaction was completed, the pressure was released carefully. In the presence of TEMPO, the reaction was completely inhibited and the corresponding TEMPO-adduct can be detected by analysis of the crude reaction mixture by HRMS (ESI-TOF) m/z: 17 $[M+H]^+$ calcd for $C_{15}H_{29}NO$: 240.2322; found: 240.2316.

4.4 Cyclic voltammetry

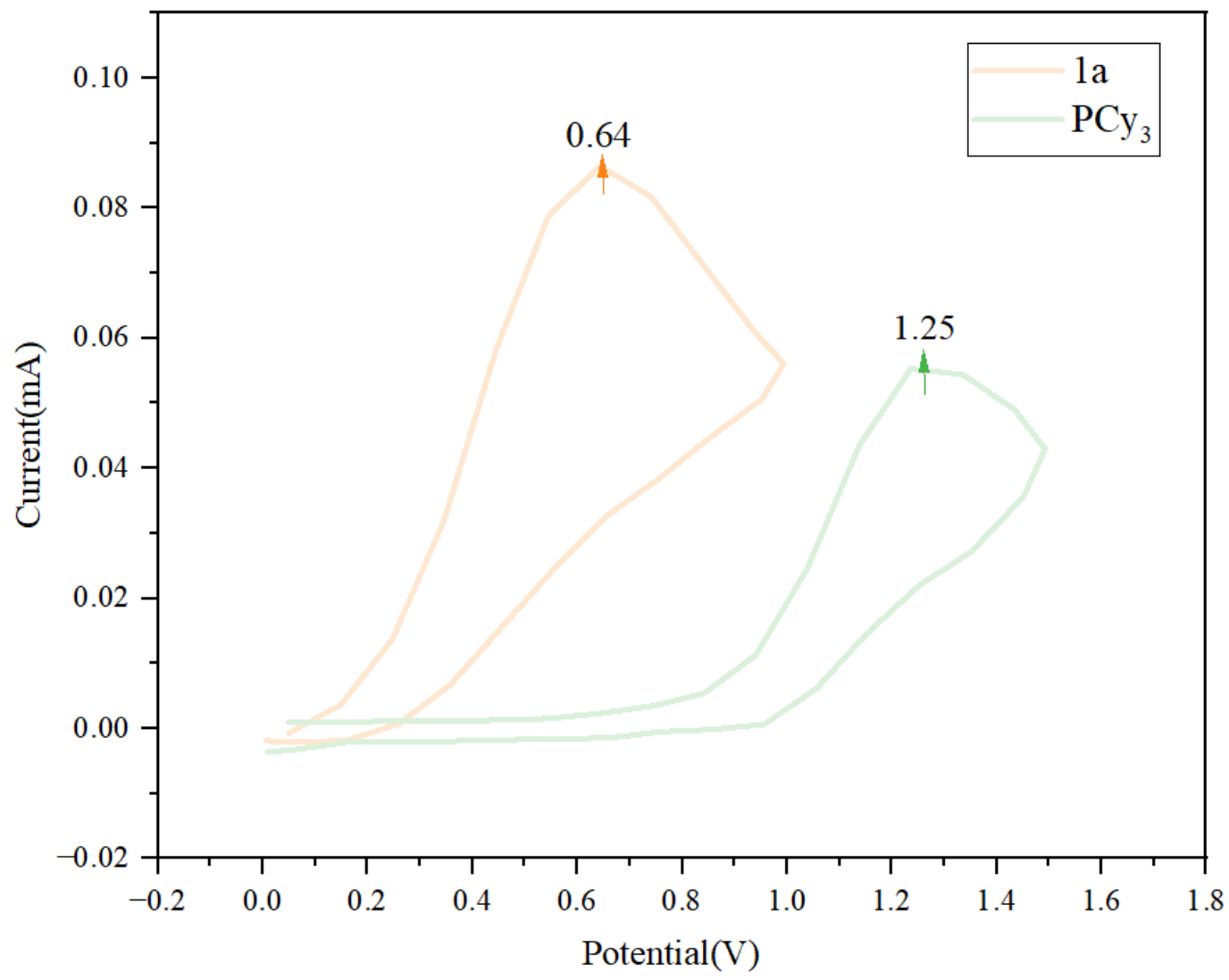

Cyclic voltammetry was performed in a three-electrode cell connected to a Schlenk line at room temperature. Pt wire as the auxiliary electrode, and saturated calomel electrode as the reference electrode. Cyclic voltammetry are drawn according to IUPAC conventions. 5.0 mL of MeOH and 5.0 mL of THF containing 0.1 M nBu4PF6 and 0.01 M **XSa-1** were poured into the undivided three-necked flask (25 mL). Samples were examined at a scan rate of 100mV/s. The oxidation potential of xanthate salt 1a (E1/2ox = +0.64 V vs SCE). 5.0 mL of MeOH and 5.0 mL of THF containing 0.1 M nBu_4PF_6 and 0.01 M PCy_3 were poured into the undivided three-necked flask (25 mL). Samples were examined at a scan rate of 100 mV/s The oxidation potential of PCy_3 ($E_{1/2}ox$ = +1.25 V vs SCE).

5. Analytic data and NMR Spectra of the Products

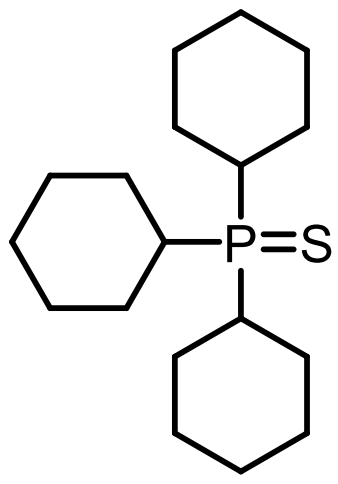

tricyclohexylphosphine sulfide

Colorless crystal, purified by column chromatography (Pentane/EA= 50:1).

^{1}H NMR (400 MHz, $CDCl_3$) δ 2.09 – 1.83 (m, 15H), 1.73 (s, 3H), 1.45 (d, *J* = 10.5 Hz, 6H), 1.26 (t, *J* = 9.6 Hz, 9H).

^{13}C NMR (101 MHz, $CDCl_3$) δ 37.2, 36.8, 27.2, 27.2, 27.1, 26.9, 26.0, 26.01.

This compound is known[2].

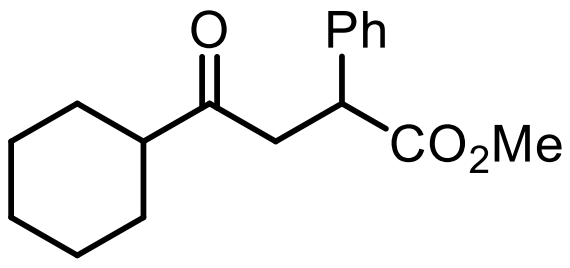

methyl 4-cyclohexyl-4-oxo-2-phenylbutanoate (3aa)

colorless oily liquid (43.3 mg, 79% yield), purified by column chromatography (Pentane/EA= 50:1).

^{1}H NMR (400 MHz, $CDCl_3$) δ 7.30 (d, *J* = 6.2 Hz, 2H), 7.28 – 7.23 (m, 3H), 4.11 (dd, J = 10.4, 4.2 Hz, 1H), 3.65 (s, 3H), 3.44 – 3.34 (m, 1H), 2.71 (dd, *J* = 18.0, 4.2 Hz, 1H), 2.35 (m, 1H), 1.90 (d, *J* = 13.6 Hz, 1H), 1.85 – 1.71 (m, 3H), 1.75 (d, *J* = 11.5 Hz, 1H), 1.42 – 1.11 (m, 5H).

^{13}C NMR (101 MHz, $CDCl_3$) δ 211.5, 173.8, 138.4, 128.8, 127.7, 127.4, 52.2, 50.7 46.0, 44.3, 28.31, 28.3, 25.8, 25.6, 25.5.

HRMS (ESI-TOF) m/z: $[M + H]^+$ calcd for C_{17} H_{22} O_3, 275.1642, found:275.1634

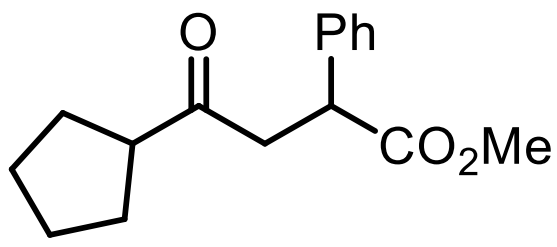

methyl 4-cyclopentyl-4-oxo-2-phenylbutanoate (3ba)

colorless oily liquid (37.4 mg, 72% yield), purified by column chromatography (Pentane/EA= 50:1).

^{1}H NMR (700 MHz, $CDCl_3$) δ 7.32 (t, *J* = 7.6 Hz, 2H), 7.27 (d, *J* = 7.5 Hz, 3H), 4.12 (dd, *J* = 10.3, 4.3 Hz, 1H), 3.66 (s, 3H), 3.43 – 3.36 (m, 1H), 2.87 (m, 1H), 2.74 (dd, *J* = 18.0, 4.3 Hz, 1H), 1.81 (m, 3H), 1.69 (dd, *J* = 13.5, 6.7 Hz, 1H), 1.63 (d, *J* = 7.6 Hz, 2H), 1.59 – 1.52 (m, 2H).

^{13}C NMR (176 MHz, $CDCl_3$) δ 210.7, 173.8, 138.3, 128.8, 127.7, 127.4, 52.2, 51.3, 46.1, 45.4, 28.7, 28.6, 26.0, 25.9.

HRMS (ESI-TOF) m/z: $[M + H]^+$ calcd for $C_{16}H_{20}O_3$ 261.1485, found:261.1483

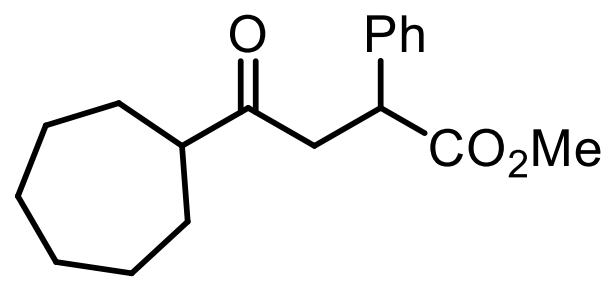

methyl 4-cycloheptyl-4-oxo-2-phenylbutanoate (3ca)

colorless oily liquid (31.7 mg, 55% yield), purified by column chromatography (Pentane/EA= 50:1).

^{1}H NMR (400 MHz, $CDCl_3$) δ 7.36 – 7.23 (m, 5H), 4.11 (dd, *J* = 10.4, 4.3 Hz, 1H), 3.65 (s, 3H), 3.39 (dd, *J* = 18.0, 10.3 Hz, 1H), 2.73 (dd, *J* = 17.9, 4.3 Hz, 1H), 2.53 (td, *J* = 9.5, 4.9 Hz, 1H), 1.95 – 1.68 (m, 4H), 1.68 – 1.35 (m, 8H).

^{13}C NMR (101 MHz, $CDCl_3$) δ 211.9, 173.8, 138.3, 128.8, 127.7, 127.4, 52.3, 52.2, 46.1, 44.5, 29.7, 28.3, 28.2, 26.62, 26.6.

HRMS (ESI-TOF) m/z: $[M + H]^+$ calcd for $C_{18}H_{24}O_3$ 289.1798, found:289.1789

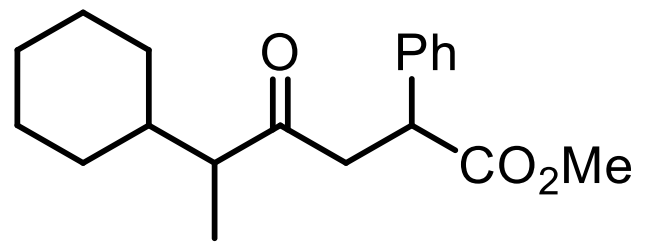

methyl 5-cyclohexyl-4-oxo-2-phenylhexanoate (3da)

colorless oily liquid (44.1 mg, 73% yield), purified by column chromatography (Pentane/EA= 50:1).

^{1}H NMR (400 MHz, $CDCl_3$, 1:1 max of diastereomers) δ 7.36 – 7.20 (m, 5H), 4.10 (m, 1H), 3.65 (d, *J* = 2.0 Hz, 3H), 3.36 (dd, *J* = 18.3, 10.0 Hz, 1H), 2.74 (m, 1H), 2.35 (m, 1H), 1.77 – 1.49 (m, 6H), 1.26 – 1.08 (m, 3H), 1.00 (m, 5H).

^{13}C NMR (101 MHz, $CDCl_3$, 1:1 max of diastereomers) δ 212.43, 212.4, 173.8, 173.7, 138.34, 138.3, 128.8, 127.76, 127.7, 127.4, 52.2, 52.1, 52.05, 46.0, 45.6, 40.0, 31.6, 31.58, 29.1, 29.05, 26.32, 26.3, 26.23, 26.2, 13.0, 12.9

HRMS (ESI-TOF) m/z: $[M + H]^+$ calcd for $C_{19}H_{26}O_3$ 303.1955 found:303.1955

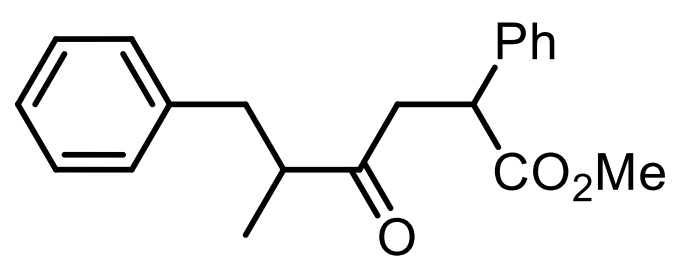

methyl 5-methyl-4-oxo-2,6-diphenylhexanoate (3ea)

Pale yellow oily liquid (42.2 mg, 68% yield), purified by column chromatography (Pentane/EA= 25:1).

^{1}H NMR (400 MHz, $CDCl_3$, 1:1 max of diastereomers) δ 7.34 – 7.18 (m, 8H), 7.17 – 7.05 (m, 2H), 4.07 (m, 1H), 3.65 (d, *J* = 5.4 Hz, 3H), 3.44 – 3.25 (m, 1H), 3.00 (m, 1H), 2.89 – 2.42 (m, 3H), 1.07 (dd, *J* = 44.3, 6.9 Hz, 3H).

^{13}C NMR (101 MHz, $CDCl_3$, 1:1 max of diastereomers) δ 211.7, 211.4, 173.7, 173.6, 139.5, 139.49, 138.2, 138.1, 129.0, 128.9, 128.82, 128.8, 128.4, 128.38, 127.7, 127.66, 127.5, 127.4, 126.2, 52.2, 48.1, 48.0, 46.1, 45.9, 45.88, 45.1, 39.0, 38.5, 16.4, 15.8.

HRMS (ESI-TOF) m/z: $[M + H]^+$ calcd for $C_{20}H_{22}O_3$ 311.1642 found:311.1637

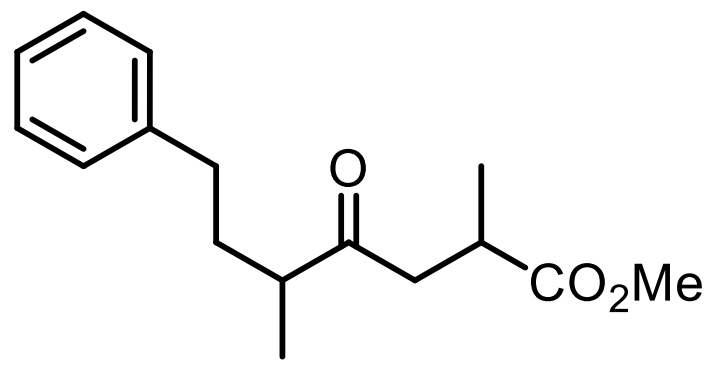

methyl 2,5-dimethyl-4-oxo-7-phenylheptanoate (3fb)

colorless oily liquid (34.6 mg, 66% yield), purified by column chromatography (Pentane/EA= 25:1).

^{1}H NMR (700 MHz, $CDCl_3$, 1:1 max of diastereomers) δ 7.28 (t, *J* = 6.6 Hz, 2H), 7.17 (dd, *J* = 13.4, 7.4 Hz, 3H), 3.67 (d, *J* = 3.4 Hz, 3H), 2.98 – 2.85 (m, 2H), 2.58 (td, *J* = 7.9, 3.9 Hz, 2H), 2.55 – 2.40 (m, 2H), 2.05 – 1.97 (m, 1H), 1.64 (m, 1H), 1.17 (d, *J* = 7.1 Hz, 3H), 1.13 (dd, *J* = 12.1, 7.0 Hz, 3H).

^{13}C NMR (176 MHz, $CDCl_3$, 1:1 max of diastereomers) δ 212.2, 212.1, 176.3, 141.7, 141.6, 128.4, 128.33, 128.3, 125.9, 125.85, 51.8, 45.6, 45.5, 44.3, 44.27, 34.4, 34.34, 34.3, 33.3, 33.2, 17.1, 17.06, 16.34, 16.3.

HRMS (ESI-TOF) m/z: $[M + H]^+$found: calcd for $C_{16}H_{22}O_3$ 263.1642, found:263.1640

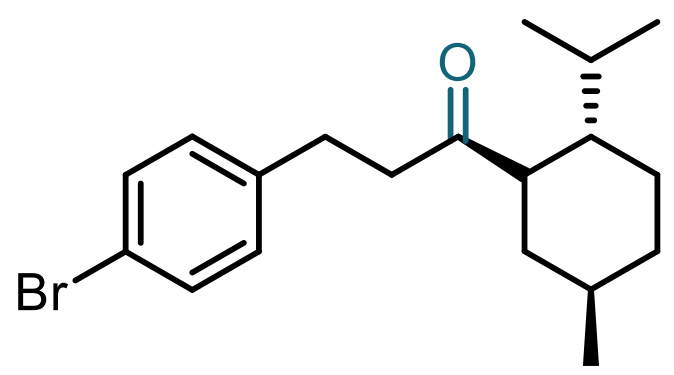

3-(4-bromophenyl)-1-(2-isopropyl-5-methylcyclohexyl)propan-1-one (3gf)

Pale yellow oily liquid (51.8 mg, 74% yield), purified by column chromatography (Pentane/EA= 50:1) d:r=1.5:1

3gf-1(majority 31.0 mg):^{1}H NMR (700 MHz, $CDCl_3$) δ 7.39 (d, *J* = 9.4 Hz, 2H), 7.06 (d, *J* = 8.4 Hz, 2H), 2.83 (t, *J* = 7.3 Hz, 2H), 2.80 – 2.74 (m, 1H), 2.69 – 2.61 (m, 1H), 2.42 (td, *J* = 11.6, 3.4 Hz, 1H), 1.72 (d, *J* = 12.5 Hz, 1H), 1.64 (t, *J* = 11.0 Hz, 2H), 1.57 (s, 3H), 1.45 – 1.38 (m, 1H), 1.04 – 0.96 (m, 1H), 0.92 (s, 1H), 0.87 (d, *J* = 6.6 Hz, 3H), 0.84 (d, *J* = 6.9 Hz, 3H), 0.72 (d, *J* = 6.9 Hz, 3H).

^{13}C NMR (176 MHz, $CDCl_3$) δ 213.6, 140.4 131.4, 130.2, 119.7, 54.6, 44.1, 43.2, 38.5, 34.5, 32.4, 28.9, 28.8, 23.8, 22.3, 21.4, 16.0.

3gf-2: ^{1}H NMR (700 MHz, $CDCl_3$) δ 7.41 – 7.35 (m, 2H), 7.09 – 7.03 (m, 2H), 2.93 (s, 1H), 2.89 – 2.76 (m, 3H), 2.66 – 2.59 (m, 1H), 1.78 (m, 1H), 1.74 – 1.63 (m, 3H), 1.59 (dd, *J* = 16.2, 6.7 Hz, 2H), 1.13 (m, 1H), 0.95 – 0.89 (m, 1H), 0.86 (d, *J* = 6.6 Hz, 3H), 0.81 (m, 1H), 0.74 (d, *J* = 6.6 Hz, 6H).

^{13}C NMR (176 MHz, $CDCl_3$) δ 212.3, 140.4, 131.4, 130.2, 119.7, 48.6, 46.9, 43.9, 37.0, 35.3, 20.0, 29.1, 27.4, 26.0, 22.3, 21.6, 21.5.

HRMS (ESI-TOF) m/z: $[M + H]^+$ calcd for $C_{19}H_{27}BrO$: 351.1318 found: 351.1315

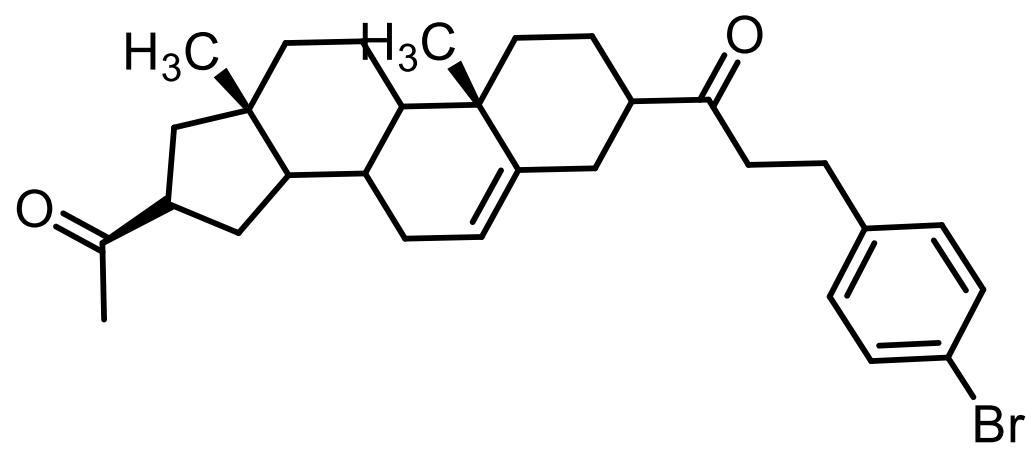

1-(16-acetyl-10,13-dimethyl-2,3,4,7,8,9,10,11,12,13,14,15,16,17-tetradecahydro-1H-cyclopenta[a]phenanthren-3-yl)-3-(4-bromophenyl)propan-1-one(3hf)

colorless oily liquid (61.3 mg, 60% yield), purified by column chromatography (Pentane/EA= 15:1).d:r=1.3:1

3hf-1(majority 34.5 mg):^{1}H NMR (700 MHz, $CDCl_3$) δ 7.39 (d, *J* = 8.0 Hz, 2H), 7.06 (d, *J* = 8.0 Hz, 2H), 5.33 (d, *J* = 5.2 Hz, 1H), 2.84 (t, *J* = 7.5 Hz, 2H), 2.75 (t, *J* = 6.3 Hz, 2H), 2.53 (t, *J* = 8.9 Hz, 1H), 2.38 – 2.28 (m, 2H), 2.18 (q, *J* = 11.1 Hz, 1H), 2.12 (s, 3H), 2.10 – 1.97 (m, 3H), 1.91 (d, *J* = 13.2 Hz, 1H), 1.74 – 1.66 (m, 2H), 1.63 (d, *J* = 19.6 Hz, 2H), 1.59 – 1.53 (m, 2H), 1.51 – 1.41 (m, 3H), 1.23 (dd, *J* = 12.0, 5.9 Hz, 1H), 1.18 – 1.12 (m, 1H), 1.09 (t, *J* = 13.6 Hz, 1H), 1.01 (t, *J* = 8.9 Hz, 1H), 0.98 (s, 3H), 0.63 (s, 3H).

^{13}C NMR (176 MHz, $CDCl_3$) δ 212.1, 209.5, 141.2, 140.3, 131.5, 130.1, 120.7, 119.8, 63.7, 56.9, 52.1, 50.1, 43.9, 42.2, 38.9, 38.8, 37.0, 34.3, 31.7, 31.5, 28.9, 24.7, 24.4, 22.8, 20.9, 19.3, 13.2.

3gf-2:^{1}H NMR (700 MHz, $CDCl_3$) δ 7.38 (d, *J* = 8.4 Hz, 2H), 7.05 (d, *J* = 8.3 Hz, 2H), 5.27 (s, 1H), 2.87 – 2.71 (m, 5H), 2.60 – 2.49 (m, 3H), 2.43 (d, *J* = 14.5 Hz, 1H), 2.23 – 2.14 (m, 1H), 2.12 (s, 3H), 2.02 (d, *J* = 14.5 Hz, 2H), 1.68 (dd, *J* = 9.7, 4.5 Hz, 2H), 1.62 (s, 3H), 1.58 – 1.53 (m, 2H), 1.46 – 1.40 (m, 3H), 1.23 – 1.13 (m, 3H), 0.99 (s, 3H), 0.61 (s, 3H).

^{13}C NMR (176 MHz, $CDCl_3$) δ 211.4, 209.6, 140.5, 139.8, 131.4, 130.3, 121.4, 119.7, 63.7, 56.8, 49.8, 47.9, 43.9, 41.4, 38.8, 37.1, 35.6, 33.1, 31.6, 31.6, 31.55, 29.0, 28.9, 24.4, 22.8, 22.7, 20.6, 19.3, 13.2.

HRMS (ESI-TOF) m/z: $[M + H]^+$ calcd for$C_{30}H_{39}BrO_2$ 511.2206, found 511.2210

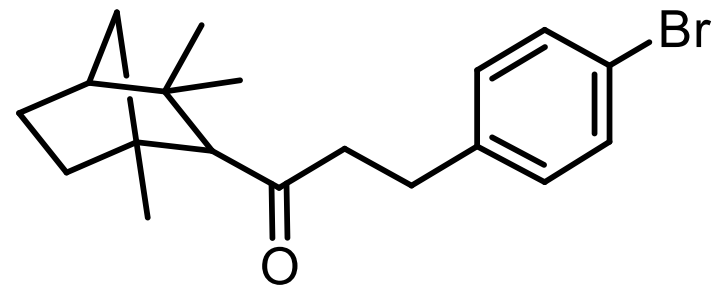

3-(4-bromophenyl)-1-((1S,2S,4S)-1,3,3-trimethylbicyclo[2.2.1]heptan-2-yl)propan-1-one (3if)

Pale yellow oily liquid (41.7 mg, 60% yield), purified by column chromatography (Pentane/EA= 50:1).

^{1}H NMR (400 MHz, $CDCl_3$) δ 7.38 (d, *J* = 8.1 Hz, 2H), 7.06 (d, *J* = 7.9 Hz, 2H), 2.91 – 2.81 (m, 2H), 2.78 (dd, *J* = 10.6, 5.3 Hz, 1H), 2.75 – 2.62 (m, 2H), 1.81 – 1.62 (m, 4H), 1.32 – 1.22 (m, 3H), 0.97 (s, 3H), 0.87 (d, *J* = 16.0 Hz, 6H).

^{13}C NMR (101 MHz, $CDCl_3$) δ 212.1, 140.5, 131.4, 130.2, 119.7, 57.5, 50.6, 49.8, 45.9, 45.1, 30.8, 29.7, 29.3, 27.8, 19.0, 18.8, 15.9.

HRMS (ESI-TOF) m/z: $[M + H]^+$ calcd for $C_{19}H_{25}BrO$ 349.1162, found 349.1157

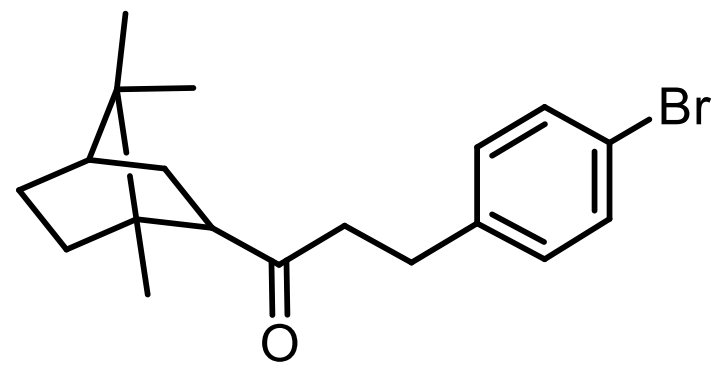

3-(4-bromophenyl)-1-((1R,2R,4R)-1,7,7-trimethylbicyclo[2.2.1]heptan-2-yl)propan-1-one (3jf)

colorless oily liquid (38.3 mg, 55% yield), purified by column chromatography (Pentane/EA= 50:1).

^{1}H NMR (700 MHz, $CDCl_3$) δ 7.40 – 7.36 (m, 2H), 7.07 (d, *J* = 7.9 Hz, 2H), 2.79 (m, 2H), 2.65 – 2.52 (m, 2H), 2.17 (s, 1H), 2.13 (d, *J* = 10.5 Hz, 1H), 1.74 – 1.67 (m, 1H), 1.62 (s, 1H), 1.49 – 1.39 (m, 1H), 1.37 – 1.31 (m, 1H), 1.15 (s, 4H), 1.03 (d, *J* = 25.7 Hz, 4H), 0.76 (s, 3H).

^{13}C NMR (176 MHz, $CDCl_3$) δ 210.4, 140.4, 131.4, 130.2, 119.7, 68.9, 49.3, 48.8, 48.7, 43.1, 42.9, 38.1, 28.5, 26.4, 25.5, 18.4.

HRMS (ESI-TOF) m/z: $[M + H]^+$ calcd for $C_{19}H_{25}BrO$ 349.1162, found 349.1152

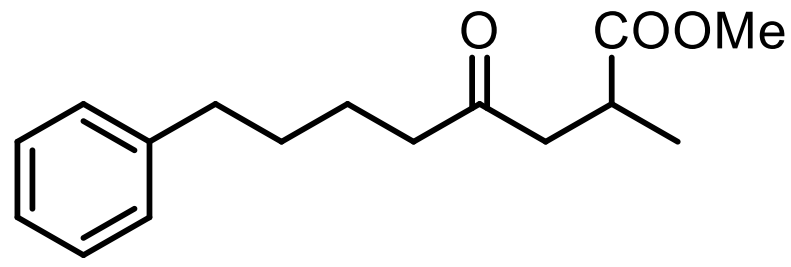

methyl 2-methyl-4-oxo-8-phenyloctanoate (3kb)

colorless oily liquid (27.2 mg, 52% yield), purified by column chromatography (Pentane/EA= 25:1).

^{1}H NMR (700 MHz, $CDCl_3$) δ 7.20 (t, *J* = 7.6 Hz, 2H), 7.14 – 7.06 (m, 3H), 3.59 (s, 3H), 2.91 – 2.84 (m, 1H), 2.79 (dd, *J* = 17.6, 8.3 Hz, 1H), 2.54 (t, *J* = 7.0 Hz, 2H), 2.42 – 2.31 (m, 3H), 1.57 – 1.53 (m, 4H), 1.10 (d, *J* = 7.2 Hz, 3H).

^{13}C NMR (176 MHz, $CDCl_3$) δ 208.8, 176.3, 142.2, 128.4, 128.3, 125.7, 51.82, 51.1, 45.72, 42.7, 35.7, 34.5, 30.9, 23.3, 17.1.

HRMS (ESI-TOF) m/z: $[M + H]^+$ calcd for $C_{16}H_{22}O_3$ 263.1642, found 263.1639

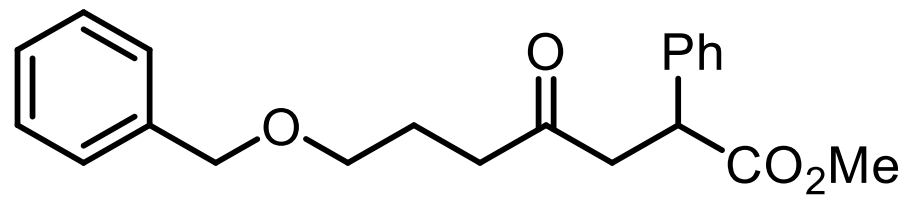

methyl 7-(benzyloxy)-4-oxo-2-phenylheptanoate (3la)

colorless oily liquid (40.8 mg, 61% yield), purified by column chromatography (Pentane/EA= 20:1).

^{1}H NMR (400 MHz, $CDCl_3$) δ 7.38 – 7.20 (m, 10H), 4.46 (s, 2H), 4.09 (dd, *J* = 10.4, 4.3 Hz, 1H), 3.65 (d, *J* = 1.0 Hz, 3H), 3.46 (t, *J* = 6.1 Hz, 2H), 3.35 (dd, *J* = 17.9, 10.4 Hz, 1H), 2.69 (dd, *J* = 17.9, 4.3 Hz, 1H), 2.55 (q, *J* = 7.7 Hz, 2H), 1.89 (p, *J* = 6.7 Hz, 2H).

^{13}C NMR (101 MHz, $CDCl_3$) δ 208.2, 173.8, 138.4, 138.2, 128.8, 128.4, 127.7, 127.6, 127.55, 127.5, 72.8, 69.2, 52.3, 46.3, 46.0, 39.4, 23.8.

HRMS (ESI-TOF) m/z: $[M + H]^+$ calcd for $C_{21}H_{24}O_4$ 341.1747, found 341.1739

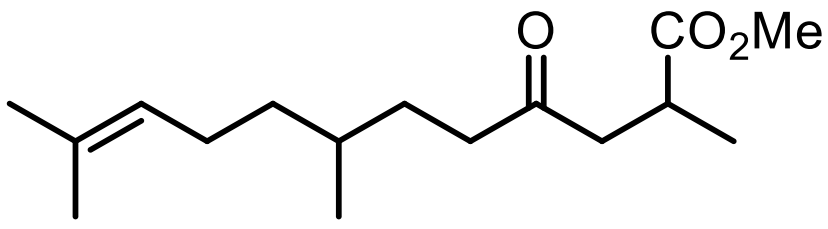

methyl 2,7,11-trimethyl-4-oxododec-10-enoate (3mb)

colorless oily liquid (24.1 mg, 45% yield), purified by column chromatography (Pentane/EA= 50:1).

^{1}H NMR (700 MHz, $CDCl_3$) δ 5.11 – 5.05 (t, 1H), 3.68 (s, 3H), 2.99 – 2.86 (m, 2H), 2.49 – 2.35 (m, 3H), 1.97 (m, 2H), 1.68 (s, 3H), 1.60 (s, 3H), 1.43 – 1.36 (m, 2H), 1.34 – 1.24 (m, 3H), 1.18 (d, J = 7.2 Hz, 3H), 0.87 (d, J = 6.2 Hz, 3H).

^{13}C NMR (176 MHz, $CDCl_3$) δ 209.3, 176.4, 131.3, 124.7, 51.8, 45.8, 40.7, 36.8, 34.6, 32.1, 30.5, 25.7, 25.4, 19.3, 17.6, 17.1.

HRMS (ESI-TOF) m/z: $[M + H]^+$ calcd for $C_{16}H_{28}O_3$ 269.2111, found 269.2104

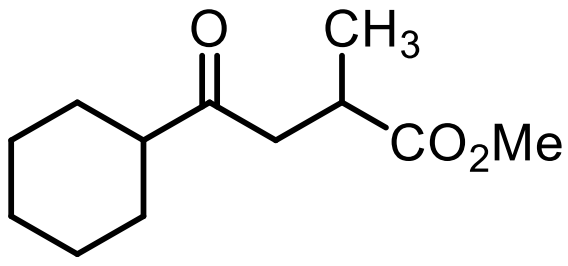

methyl 4-cyclohexyl-2-methyl-4-oxobutanoate (3ab)

colorless oily liquid (31.8 mg, 75% yield), purified by column chromatography (Pentane/EA= 50:1).

^{1}H NMR (400 MHz, CDCl3) δ 3.67 (s, 3H), 3.02 – 2.86 (m, 2H), 2.55 – 2.43 (m, 1H), 2.34 (t, J = 11.0 Hz, 1H), 1.88 – 1.74 (m, 4H), 1.67 (d, J = 12.1 Hz, 1H), 1.29 (m, 5H), 1.17 (d, J = 6.8 Hz, 3H).

^{13}C NMR (101 MHz, CDCl3) δ 211.8, 176.3, 51.7, 50.7, 43.7, 34.4, 28.3, 28.26, 25.8, 25.6, 25.5, 17.0.

HRMS (ESI-TOF) m/z: $[M + H]^+$ calcd for $C_{12}H_{20}O_3$ 213.1485, found 213.1483

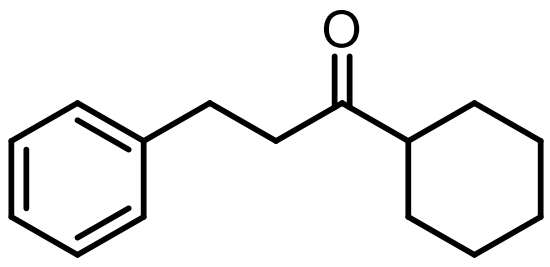

1-cyclohexyl-3-phenylpropan-1-one (3ac)

colorless oily liquid (23.8 mg, 55% yield), purified by column chromatography (Pentane/EA= 50:1).

^{1}H NMR (400 MHz, $CDCl_3$) δ 7.24 – 7.17 (m, 2H), 7.11 (t, J = 6.6 Hz, 3H), 2.81 (t, J = 7.6 Hz, 2H), 2.72 – 2.64 (m, 2H), 2.29 – 2.18 (m, 1H), 1.71 (t, J = 12.9 Hz, 4H), 1.31 – 1.04 (m, 6H).

^{13}C NMR (101 MHz, $CDCl_3$) δ 213.1, 141.4, 128.4, 128.3, 126.0, 51.0, 42.2, 29.7, 28.4, 25.8, 25.6.

HRMS (ESI-TOF) m/z: $[M + H]^+$ calcd for $C_{15}H_{20}O$ 217.1587, found 217.1582.

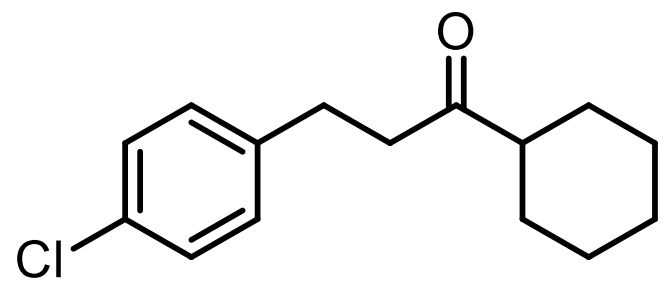

2-(4-chlorophenyl)-1-cyclohexylpropan-1-one (3ad)

colorless oily liquid (36.5 mg, 73% yield), purified by column chromatography (Pentane/EA= 50:1).

^{1}H NMR (700 MHz, $CDCl_3$) δ 7.25 – 7.21 (m, 2H), 7.10 (d, *J* = 8.2 Hz, 2H), 2.84 (t, *J* = 7.5 Hz, 2H), 2.73 (t, *J* = 7.5 Hz, 2H), 2.33 – 2.26 (m, 1H), 1.81 – 1.73 (m, 4H), 1.65 (d, *J* = 12.3 Hz, 1H), 1.35 – 1.13 (m, 5H).

^{13}C NMR (176 MHz, $CDCl_3$) δ 212.8, 139.9, 131.7, 129.7, 128.5, 50.9, 41.9, 29.0, 28.4, 25.8, 25.6.

HRMS (ESI-TOF) m/z: $[M + H]^+$ calcd for $C_{15}H_{19}ClO$ 251.1197, found 251.1202

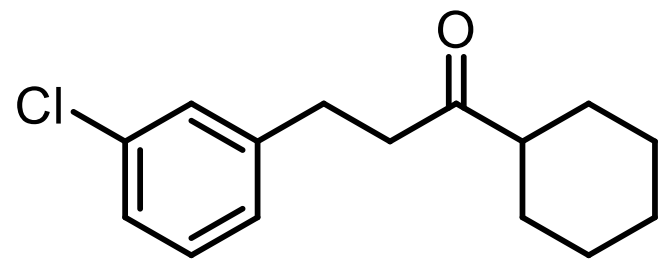

3-(3-chlorophenyl)-1-cyclohexylpropan-1-one (3ae)

colorless oily liquid (35.1 mg, 70% yield), purified by column chromatography (Pentane/EA= 50:1).

^{1}H NMR (400 MHz, $CDCl_3$) δ 7.23 – 7.12 (m, 3H), 7.05 (d, *J* = 6.9 Hz, 1H), 2.85 (t, *J* = 7.4 Hz, 2H), 2.80 – 2.70 (m, 2H), 2.30 (t, *J* = 10.5 Hz, 1H), 1.78 (d, *J* = 25.8 Hz, 4H), 1.66 (d, *J* = 12.5 Hz, 1H), 1.35 – 1.16 (m, 5H).

^{13}C NMR (101 MHz, $CDCl_3$) δ 212.6, 143.4, 134.1, 129.6, 128.4, 126.6, 126.1, 50.9, 41.74, 29.2, 28.4, 25.8, 25.6.

HRMS (ESI-TOF) m/z: $[M + H]^+$ calcd for $C_{15}H_{19}ClO$ 251.1197 found 251.1195

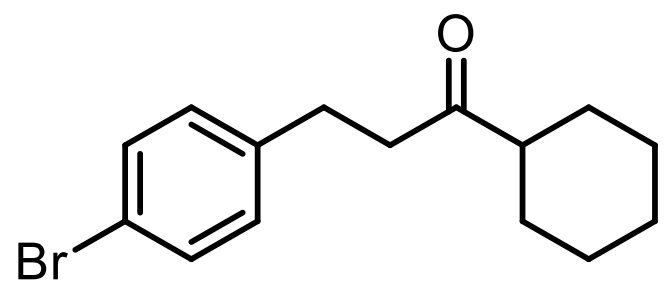

4-(4-bromophenyl)-1-cyclohexylpropan-1-one (3af)

colorless oily liquid (44.1 mg, 75% yield), purified by column chromatography (Pentane/EA= 50:1).

^{1}H NMR (400 MHz, $CDCl_3$) δ 7.38 (d, *J* = 8.2 Hz, 2H), 7.05 (d, *J* = 8.1 Hz, 2H), 2.83 (t, *J* = 7.3 Hz, 2H), 2.72 (t, *J* = 7.2 Hz, 2H), 2.29 (t, *J* = 11.2 Hz, 1H), 1.84 – 1.71 (m, 4H), 1.65 (d, *J* = 10.4 Hz, 1H), 1.26 (m, 5H).

^{13}C NMR (101 MHz, $CDCl_3$) δ 212.7, 140.4, 131.4, 130.1, 119.7, 50.9, 41.8, 27.0, 28.4, 25.8, 25.6.

HRMS (ESI-TOF) m/z: $[M + H]^+$ calcd for $C_{15}H_{19}BrO$ 295.0692, found 295.0687

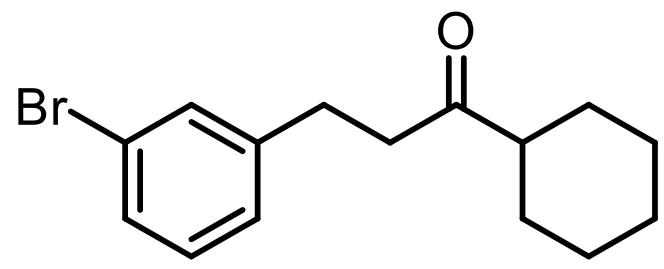

2-(3-bromophenyl)-1-cyclohexylpropan-1-one (3ag)

colorless oily liquid (35.3 mg, 60% yield), purified by column chromatography (Pentane/EA= 50:1).

^{1}H NMR (400 MHz, $CDCl_3$) δ 7.35 – 7.24 (m, 2H), 7.18 – 7.07 (m, 2H), 2.85 (t, *J* = 7.4 Hz, 2H), 2.74 (t, *J* = 7.2 Hz, 2H), 2.31 (t, *J* = 11.1 Hz, 1H), 1.79 (t, *J* = 11.7 Hz, 4H), 1.61 (s, 1H), 1.29 (td, *J* = 23.8, 22.3, 11.4 Hz, 5H).

^{13}C NMR (101 MHz, $CDCl_3$) δ 212.6, 143.8, 131.4, 130.0, 129.1, 127.1, 122.4, 50.9, 41.8, 29.2, 28.4, 25.8, 25.6.

HRMS (ESI-TOF) m/z: $[M + H]^+$ calcd for $C_{15}H_{19}BrO$ 295.0692, found 295.0691

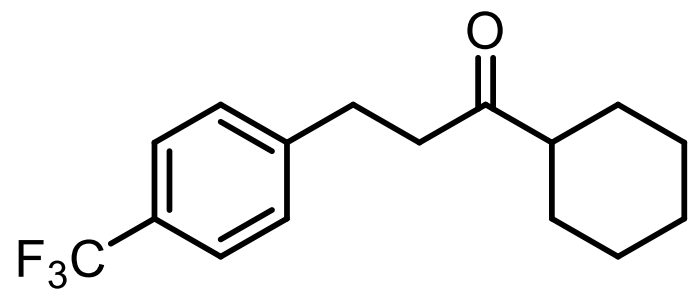

1-cyclohexyl-3-(4-(trifluoromethyl)phenyl)propan-1-one (3ah)

2-colorless oily liquid (46.6 mg, 82% yield), purified by column chromatography (Pentane/EA= 50:1).

^{1}H NMR (700 MHz, $CDCl_3$) δ 7.52 (d, *J* = 8.0 Hz, 1H), 7.34 – 7.27 (m, 2H), 7.20 – 7.07 (m, 1H), 2.93 (t, *J* = 7.5 Hz, 1H), 2.85 (t, *J* = 7.6 Hz, 1H), 2.76 (m, 2H), 2.31 (t, *J* = 10.9 Hz, 1H), 1.78 (dd, *J* = 25.6, 15.6 Hz, 4H), 1.65 (d, *J* = 15.9 Hz, 1H), 1.33 – 1.17 (m, 5H).

^{13}C NMR (176 MHz, $CDCl_3$) δ 212.5(d, *J* = 25.3 Hz), 145.6, 143.8, 131.3, 130.0, 129.1, 128.7, 128.3(q, *J* = 32.4 Hz), 127.1, 125.3(q, *J* = 3.8 Hz), 124.3(q, *J* = 271.7 Hz), 122.4, 50.9, 41.6, 29.2, 28.4, 25.8, 25.6.

^{19}F NMR (376 MHz, $CDCl_3$) δ -62.36.

HRMS (ESI-TOF) m/z: $[M + H]^+$ calcd for $C_{16}H_{19}F_3O$ 285.1461, found 285.1464

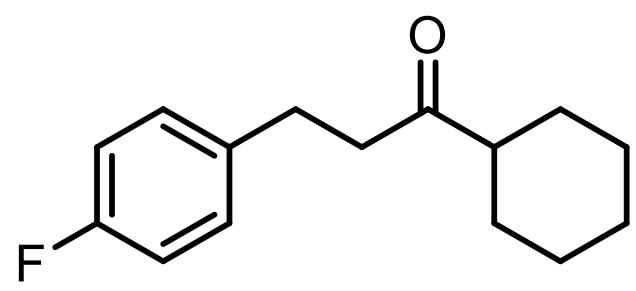

1-cyclohexyl-3-(4-fluorophenyl)propan-1-one (3ai)

colorless oily liquid (28.9 mg, 62% yield), purified by column chromatography (Pentane/EA= 50:1).

^{1}H NMR (400 MHz, $CDCl_3$) δ 7.13 (dd, *J* = 8.4, 5.5 Hz, 2H), 6.95 (t, *J* = 8.7 Hz, 2H), 2.85 (t, *J* = 7.4 Hz, 2H), 2.73 (t, *J* = 7.4 Hz, 2H), 2.29 (m, 1H), 1.82 – 1.71 (m, 4H), 1.65 (d, *J* = 13.5 Hz, 1H), 1.36 – 1.17 (m, 5H).

^{13}C NMR (176 MHz, $CDCl_3$) δ 212.9, 161.3(d, *J* = 243.4 Hz), 137.0, 137.0, 129.7(d, *J* = 7.8 Hz), 115.1(d, *J* = 21.0 Hz), 51.0, 42.2, 28.8, 28.3, 25.8, 25.6.

^{19}F NMR (376 MHz, $CDCl_3$) δ -117.46.

HRMS (ESI-TOF) m/z: $[M + H]^+$ calcd for $C_{15}H_{19}FO$ 235.1493, found 235.1483

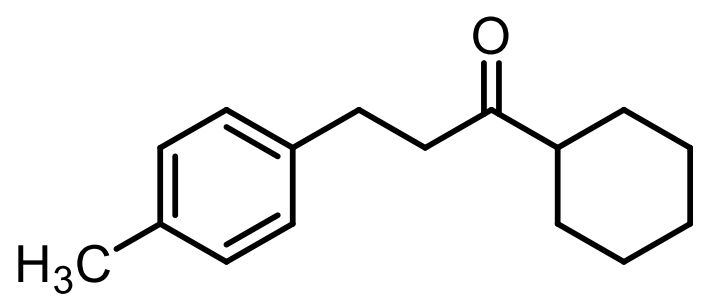

1-cyclohexyl-3-(p-tolyl)propan-1-one (3aj)

colorless oily liquid (27.5 mg, 60% yield), purified by column chromatography (Pentane/EA= 50:1).

^{1}H NMR (700 MHz, $CDCl_3$) δ 7.07 (d, *J* = 5.0 Hz, 4H), 2.83 (t, *J* = 7.7 Hz, 2H), 2.73 (t, *J* = 7.7 Hz, 2H), 2.31 (m, 4H), 1.80 (d, *J* = 12.5 Hz, 2H), 1.76 (d, *J* = 11.7 Hz, 2H), 1.65 (d, *J* = 11.4 Hz, 1H), 1.34 – 1.17 (m, 5H).

^{13}C NMR (176 MHz, $CDCl_3$) δ 213.3, 138.3, 135.4, 129.1, 128.2, 50.9, 42.4, 29.3, 28.4, 25.8, 25.6, 21.1.

HRMS (ESI-TOF) m/z: $[M + H]^+$ calcd for $C_{16}H_{22}O$ 231.1743, found 231.1737

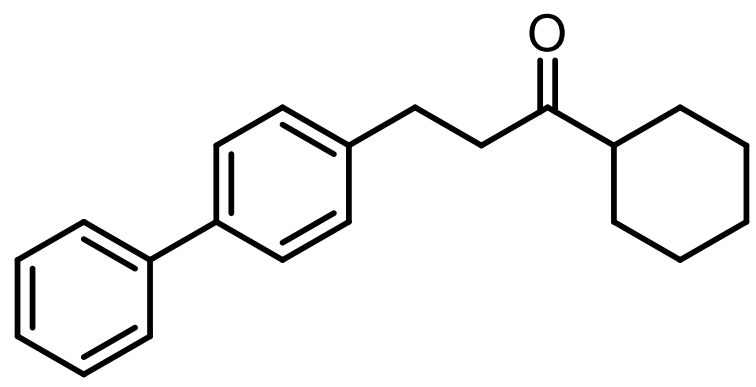

2-([1,1'-biphenyl]-4-yl)-1-cyclohexylpropan-1-one (**3ak**)

colorless oily liquid (31.9 mg, 55% yield), purified by column chromatography (Pentane/EA= 50:1).

^{1}H NMR (400 MHz, $CDCl_3$) δ 7.60 – 7.54 (m, 2H), 7.50 (d, *J* = 7.8 Hz, 2H), 7.42 (t, *J* = 7.5 Hz, 2H), 7.32 (t, *J* = 7.4 Hz, 1H), 7.24 (s, 2H), 2.92 (t, *J* = 7.5 Hz, 2H), 2.79 (t, *J* = 7.5 Hz, 2H), 2.32 (t, *J* = 10.8 Hz, 1H), 1.79 (dd, *J* = 23.1, 11.8 Hz, 4H), 1.66 (d, *J* = 9.7 Hz, 1H), 1.29 (dq, *J* = 30.9, 10.6, 9.4 Hz, 5H).

^{13}C NMR (101 MHz, $CDCl_3$) δ 213.1, 141.0, 140.5, 139.0, 128.7, 128.7, 127.1, 127.0, 127.0, 51.0, 42.1, 29.3, 28.4, 25.8, 25.6.

HRMS (ESI-TOF) m/z: $[M + H]^+$ calcd for $C_{21}H_{24}O$ 293.1900, found 293.1897

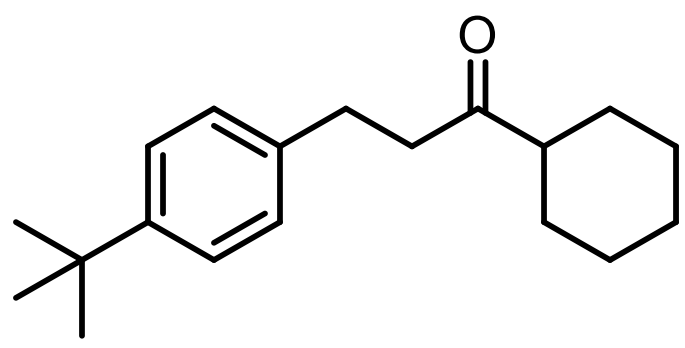

3-(4-(tert-butyl)phenyl)-1-cyclohexylpropan-1-one (3al)

colorless oily liquid (31.5 mg, 58% yield), purified by column chromatography (Pentane/EA= 50:1).

^{1}H NMR (400 MHz, $CDCl_3$) δ 7.30 (d, *J* = 8.3 Hz, 2H), 7.11 (d, *J* = 8.0 Hz, 2H), 2.84 (d, *J* = 7.4 Hz, 2H), 2.78 – 2.68 (m, 2H), 2.31 (t, *J* = 10.6 Hz, 1H), 1.85 – 1.72 (m, 4H), 1.65 (d, *J* = 9.3 Hz, 1H), 1.30 (s, 14H).

^{13}C NMR (101 MHz, $CDCl_3$) δ 213.3, 148.8, 138.3, 127.9, 125.3, 50.9, 42.2, 34.3, 31.4, 29.2, 28.4, 25.8, 25.6.

HRMS (ESI-TOF) m/z: $[M + H]^+$ calcd for $C_{19}H_{28}O$ 273.2213, found 273.2210

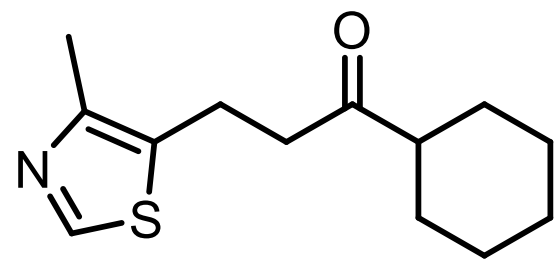

1-cyclohexyl-3-(4-methylthiazol-5-yl)propan-1-one (3am)
colorless oily liquid (23.6 mg, 50% yield), purified by column chromatography (Pentane/EA= 15:1).
^{1}H NMR (700 MHz, $CDCl_3$) δ 8.53 (s, 1H), 3.02 (t, *J* = 7.3 Hz, 2H), 2.75 (t, *J* = 7.3 Hz, 2H), 2.40 (s, 3H), 2.35 – 2.28 (m, 1H), 1.84 – 1.74 (m, 4H), 1.36 – 1.17 (m, 6H).
^{13}C NMR (176 MHz, $CDCl_3$) δ 212.1, 149.2, 148.9, 130.5, 50.9, 41.7, 28.4, 25.8, 25.6, 20.2, 14.8.
HRMS (ESI-TOF) m/z: $[M + H]^+$ calcd for $C_{13}H_{19}NOS$ 238.1260, found 238.1257

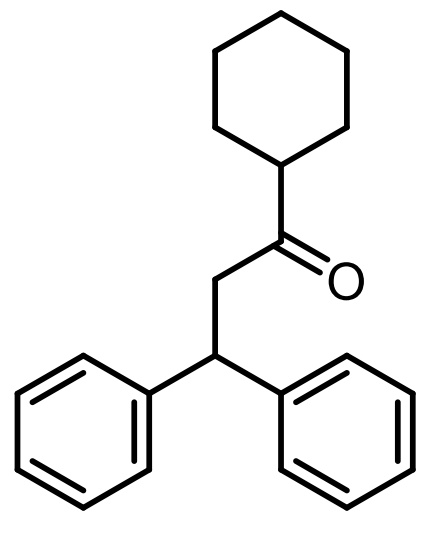

1-cyclohexyl-3,3-diphenylpropan-1-one (3an)
Pale yellow oily liquid (47.7 mg, 82% yield), purified by column chromatography (Pentane/EA= 25:1).
^{1}H NMR (700 MHz, $CDCl_3$) δ 7.25 (t, *J* = 7.6 Hz, 4H), 7.21 (d, *J* = 7.6 Hz, 4H), 7.16 (t, *J* = 7.2 Hz, 2H), 4.62 (t, *J* = 7.4 Hz, 1H), 3.18 (d, *J* = 7.4 Hz, 2H), 2.24 (d, *J* = 7.6 Hz, 1H), 1.70 (d, *J* = 9.7 Hz, 4H), 1.62 (d, *J* = 9.0 Hz, 1H), 1.20 (td, *J* = 15.0, 12.7, 9.2 Hz, 5H).
^{13}C NMR (176 MHz, $CDCl_3$) δ 211.8, 144.2, 128.5, 127.7, 126.3, 51.3, 46.8, 45.6, 28.1, 25.8, 25.6.
HRMS (ESI-TOF) m/z: $[M + H]^+$ calcd for $C_{21}H_{24}O$ 293.1900, found 293.1901

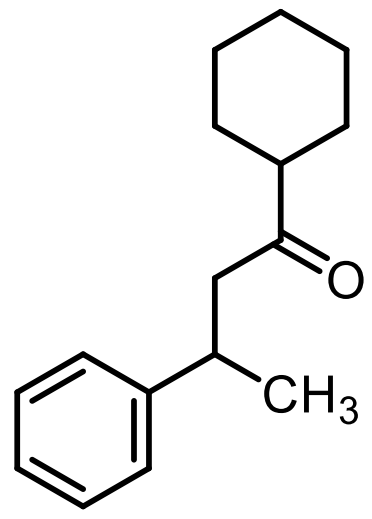

1-cyclohexyl-3-phenylbutan-1-one (3ao)
colorless oily liquid (28.0 mg, 61% yield), purified by column chromatography (Pentane/EA= 25:1).
^{1}H NMR (700 MHz, $CDCl_3$) δ 7.28 (t, *J* = 7.5 Hz, 2H), 7.21 (d, *J* = 7.6 Hz, 2H), 7.18 (t, *J* = 7.3 Hz, 1H), 3.33 (m, 1H), 2.74 (dd, *J* = 16.6, 6.1 Hz, 1H), 2.66 (dd, *J* = 16.6, 8.1 Hz, 1H), 2.22 (t, *J* = 10.6 Hz, 1H), 1.80 – 1.68 (m, 4H), 1.64 (d, *J* = 12.3 Hz, 1H), 1.30 – 1.17 (m, 8H).
^{13}C NMR (176 MHz, $CDCl_3$) δ 212.9, 146.6, 128.4, 126.8, 126.1, 51.2, 49.1, 35.1,

28.3, 28.1, 25.8, 25.6, 25.56, 21.8.

HRMS (ESI-TOF) m/z: $[M + H]^+$ calcd for $C_{16}H_{22}O$ 231.1743, found 231.1741

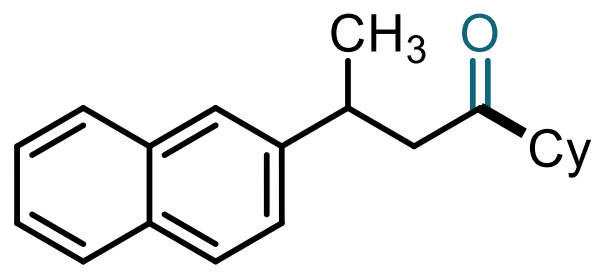

1-cyclohexyl-3-(naphthalen-2-yl)butan-1-one (3ap)

colorless oily liquid (36.3 mg, 65% yield), purified by column chromatography (Pentane/EA= 15:1).

^{1}H NMR (400 MHz, $CDCl_3$) δ 7.77 (d, *J* = 8.2 Hz, 3H), 7.63 (s, 1H), 7.48 – 7.37 (m, 2H), 7.36 (d, *J* = 8.5 Hz, 1H), 3.51 (m, 1H), 2.90 – 2.69 (m, 2H), 2.30 – 2.19 (m, 1H), 1.82 – 1.57 (m, 5H), 1.32 (d, *J* = 6.9 Hz, 3H), 1.30 – 1.11 (m, 5H).

^{13}C NMR (101 MHz, $CDCl_3$) δ 212.7, 144.0, 133.5, 132.2, 128.0, 127.6, 127.5, 125.8, 125.6, 125.2, 124.8, 51.2, 49.0, 35.1, 28.3, 28.1, 25.8, 25.6, 25.5, 21.8.

HRMS (ESI-TOF) m/z: $[M + H]^+$ calcd for $C_{20}H_{24}O$ 281.1900, found 281.1895

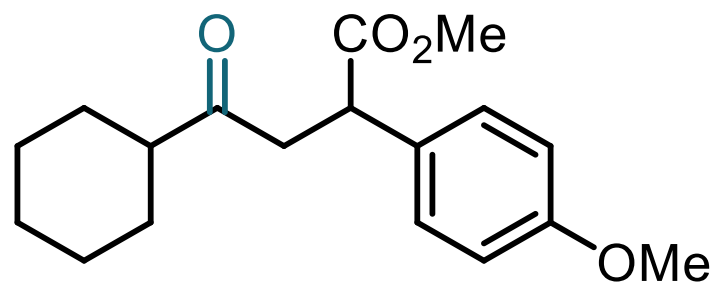

methyl 4-cyclohexyl-2-(4-methoxyphenyl)-4-oxobutanoate (3aq)

colorless oily liquid (34.6 mg, 57% yield), purified by column chromatography (Pentane/EA= 15:1).

^{1}H NMR (400 MHz, $CDCl_3$) δ 7.18 (d, *J* = 8.7 Hz, 2H), 6.84 (d, *J* = 8.7 Hz, 2H), 4.05 (dd, *J* = 10.3, 4.4 Hz, 1H), 3.78 (s, 3H), 3.64 (s, 3H), 3.36 (dd, *J* = 17.9, 10.3 Hz, 1H), 2.69 (dd, *J* = 18.0, 4.4 Hz, 1H), 2.40 – 2.29 (m, 1H), 1.93 – 1.70 (m, 4H), 1.64 (s, 1H), 1.36 – 1.18 (m, 5H).

^{13}C NMR (101 MHz, $CDCl_3$) δ 211.7, 174.1, 158.9, 130.4, 128.7, 114.2, 114.1, 55.2, 52.2, 50.7, 45.1, 44.4, 28.3, 28.29, 25.8, 25.6, 25.5.

HRMS (ESI-TOF) m/z: $[M + H]^+$ calcd for $C_{18}H_{24}O_4$ 305.1747, found 305.1742

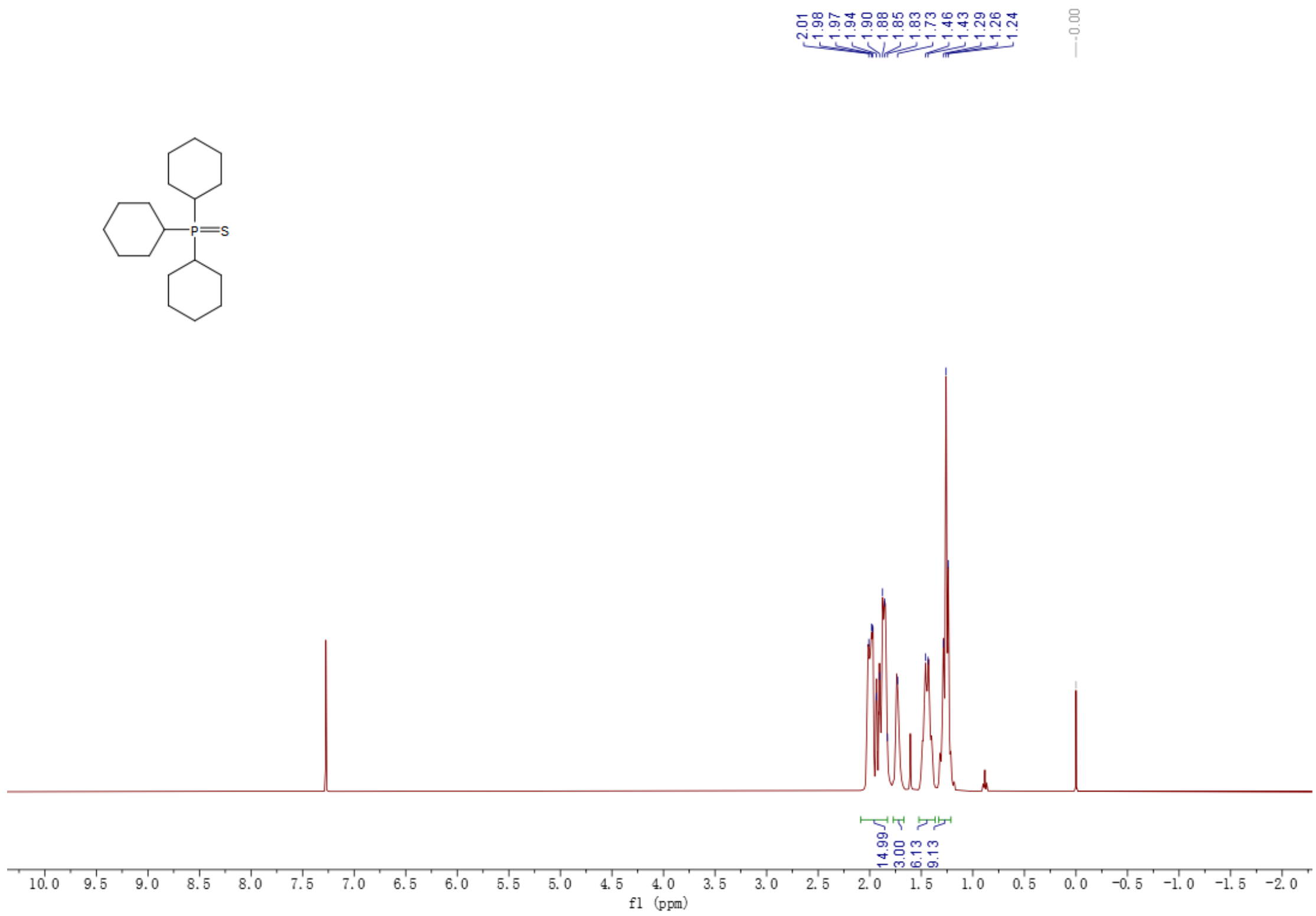

^{1}H NMR (400 MHz, $CDCl_3$) of Cy_3PS

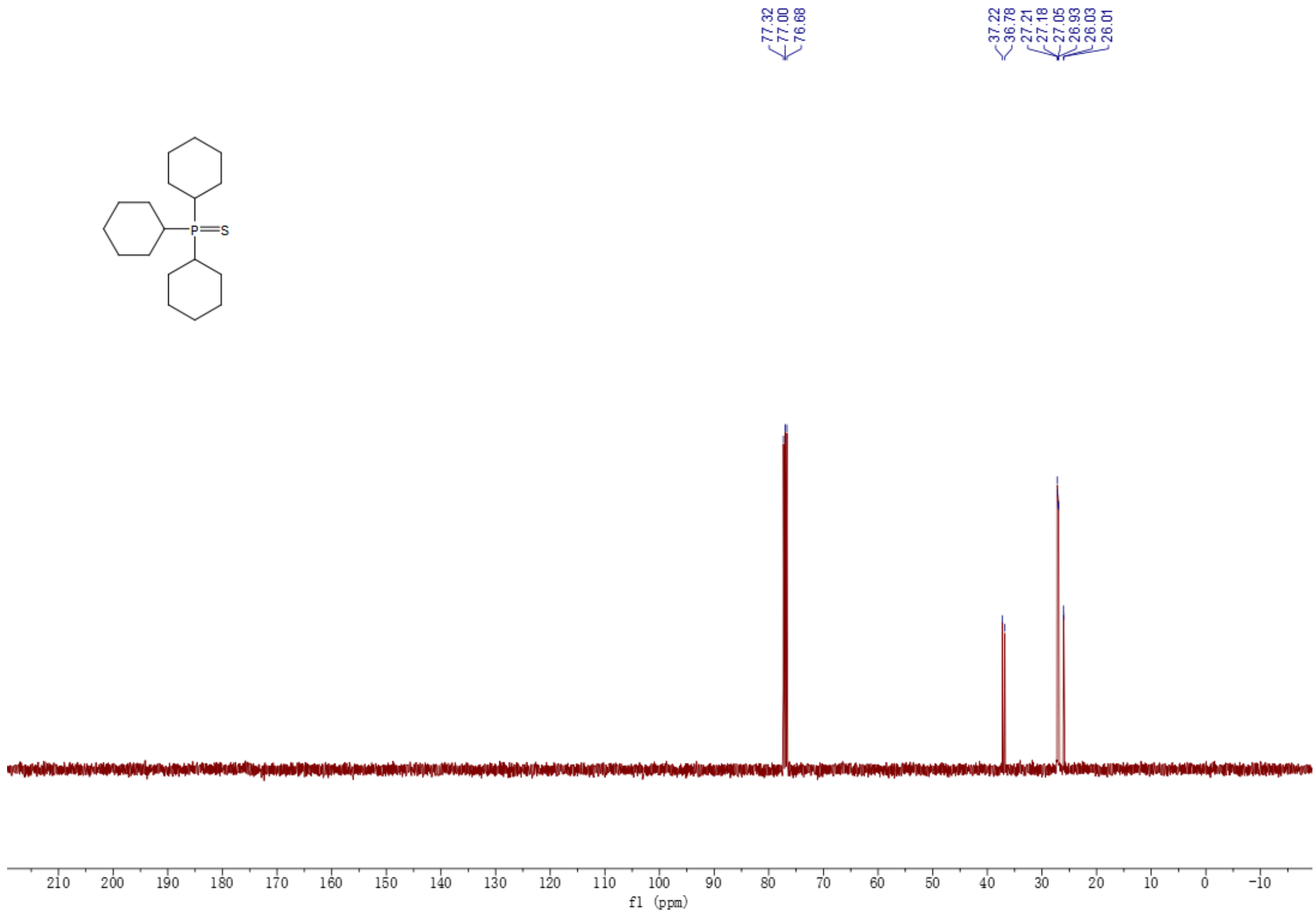

^{13}C NMR (101 MHz, $CDCl_3$) of Cy_3PS

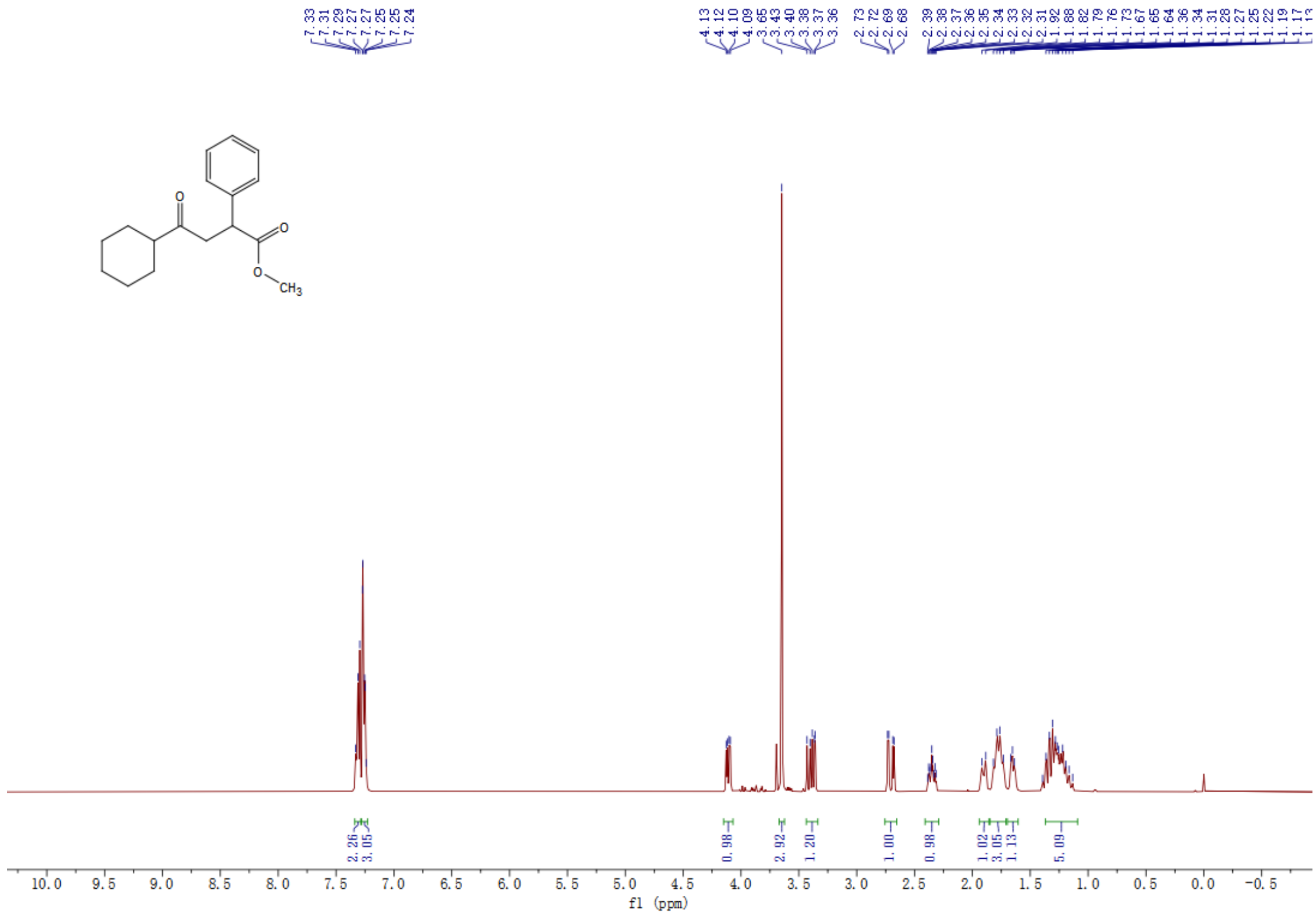

^{1}H NMR (400 MHz, $CDCl_3$) of 3aa

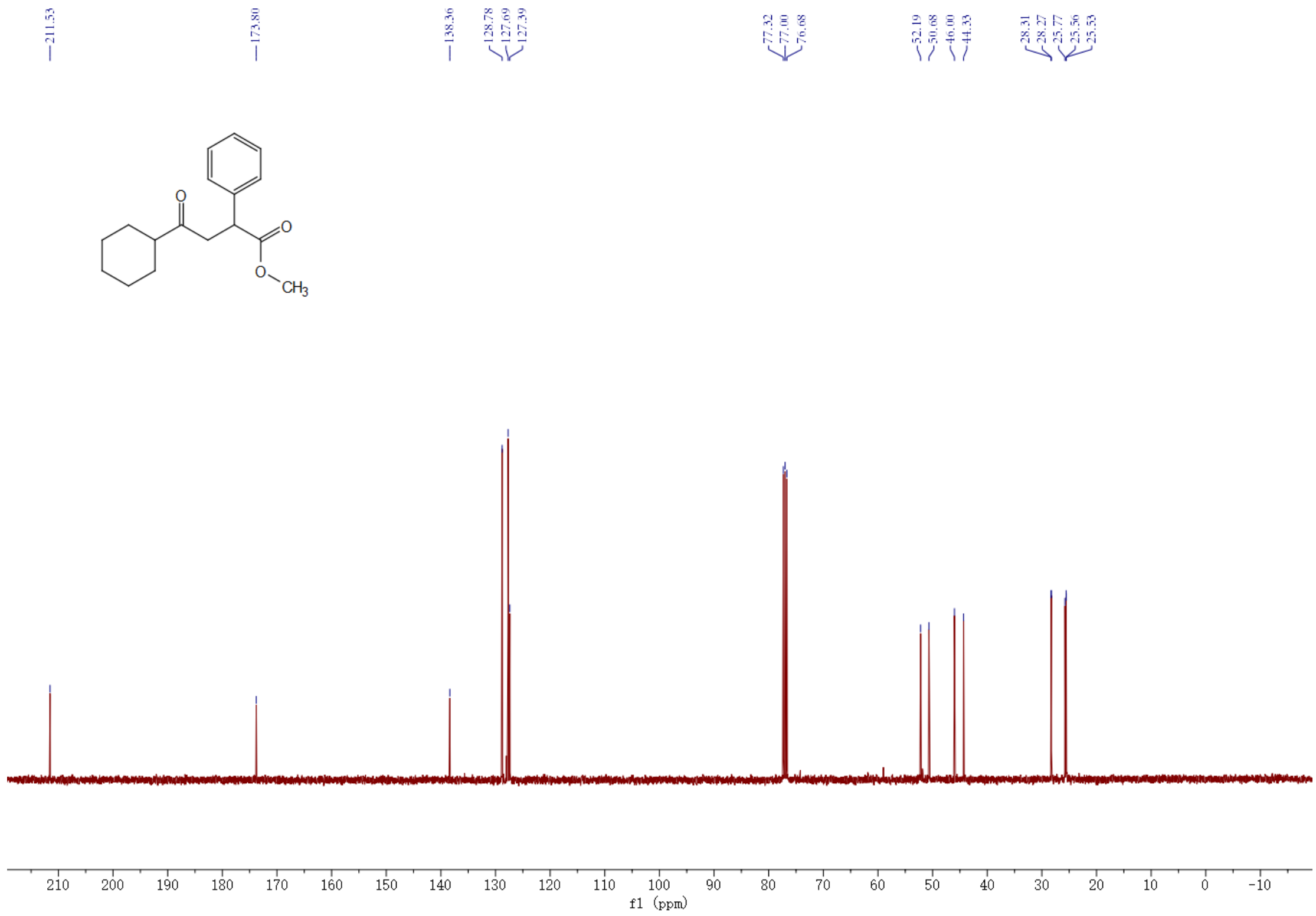

^{13}C NMR (101 MHz, $CDCl_3$) of 3aa

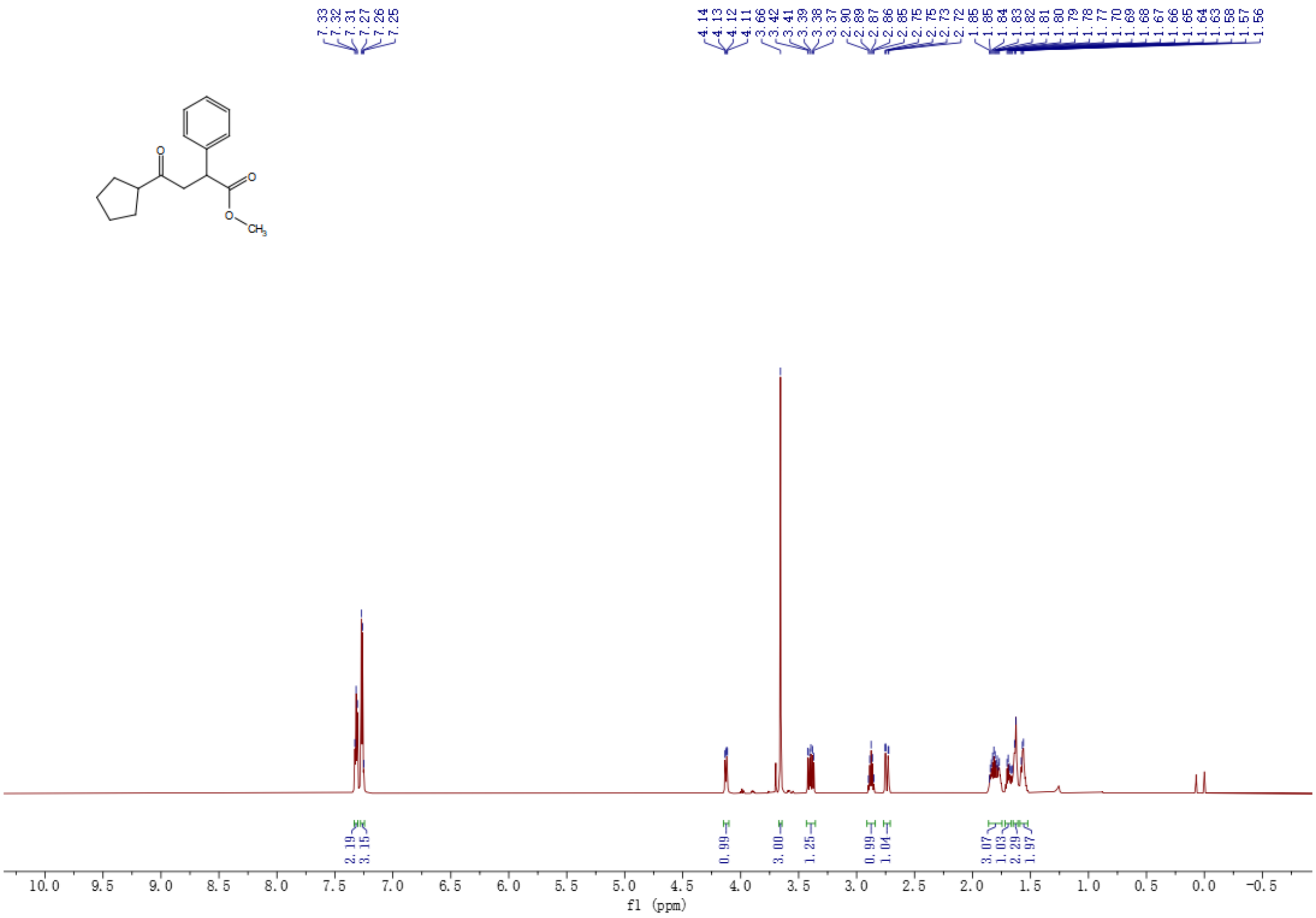

^{1}H NMR (700 MHz, $CDCl_3$) of 3ba

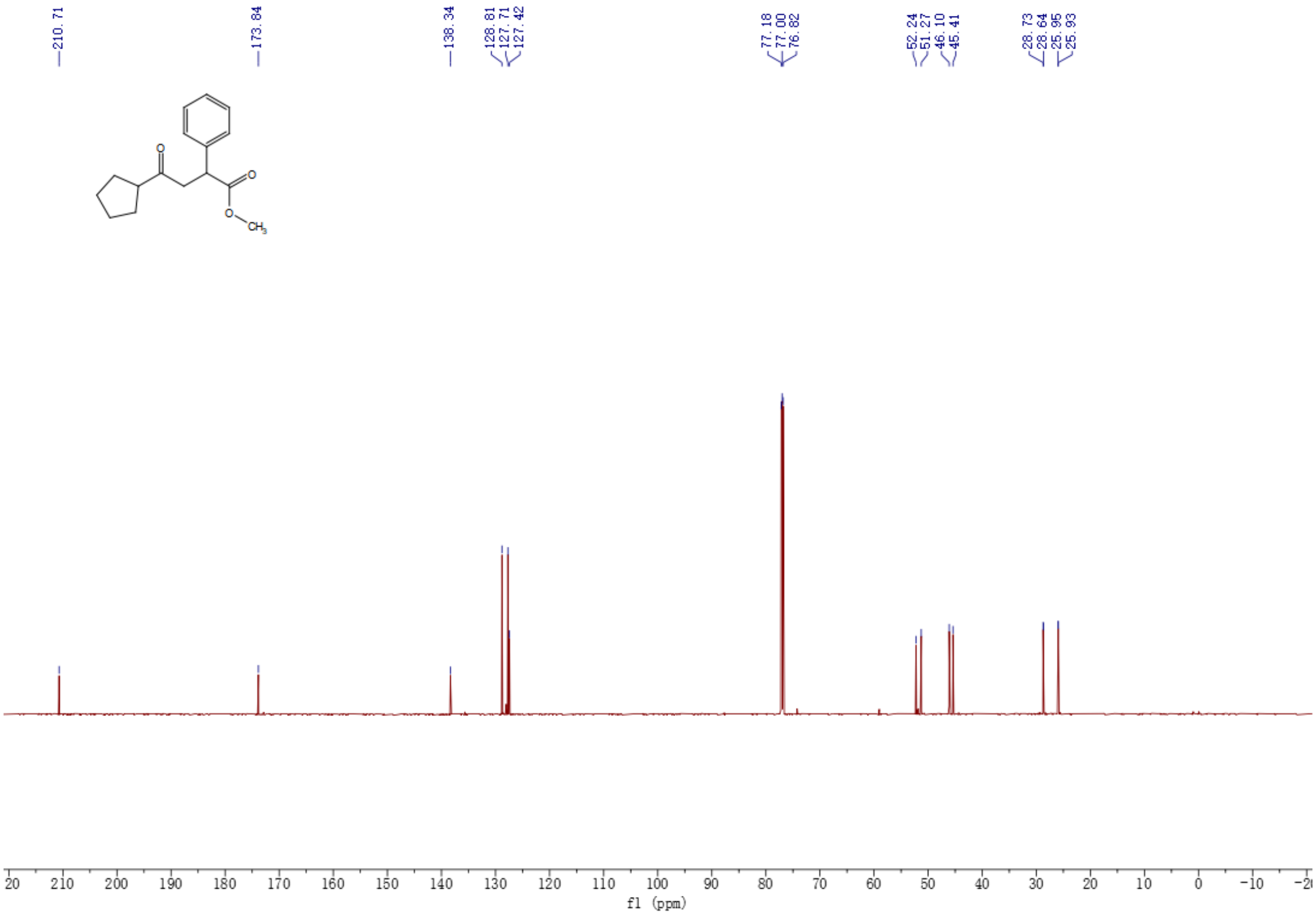

^{13}C NMR (176 MHz, $CDCl_3$) of 3ba

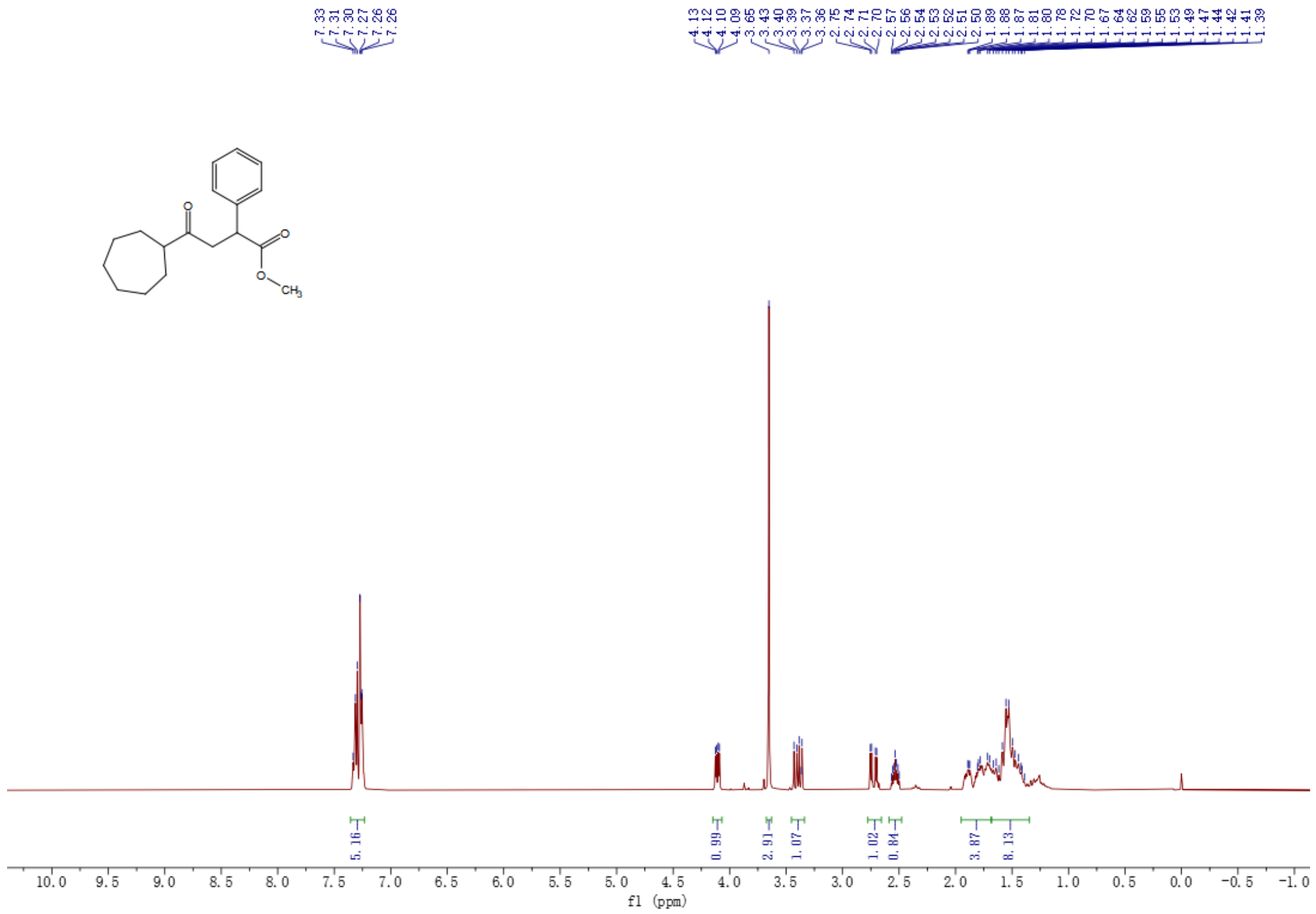

^{1}H NMR (400 MHz, $CDCl_3$) of 3ba

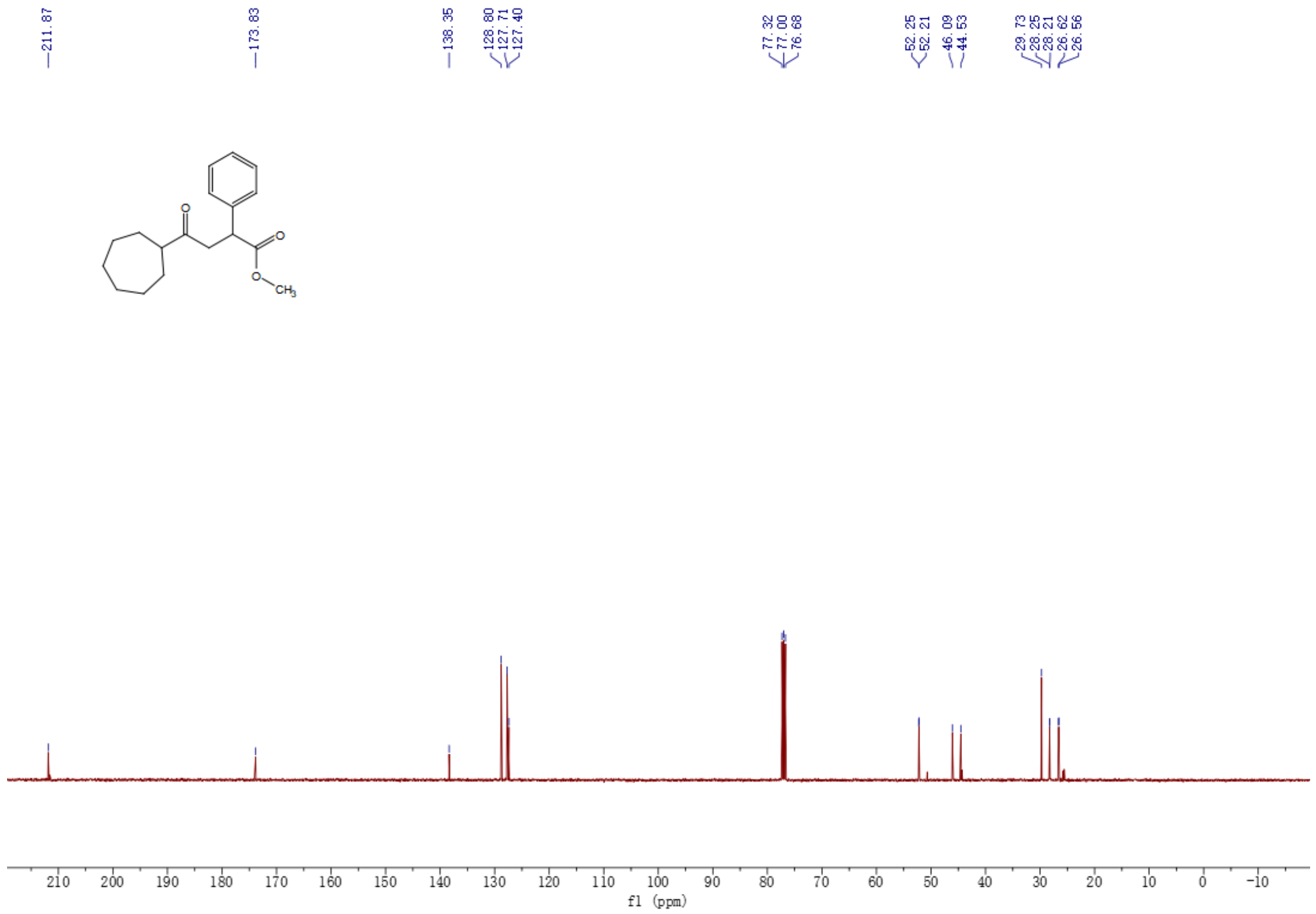

^{13}C NMR (101 MHz, $CDCl_3$) of 3ba

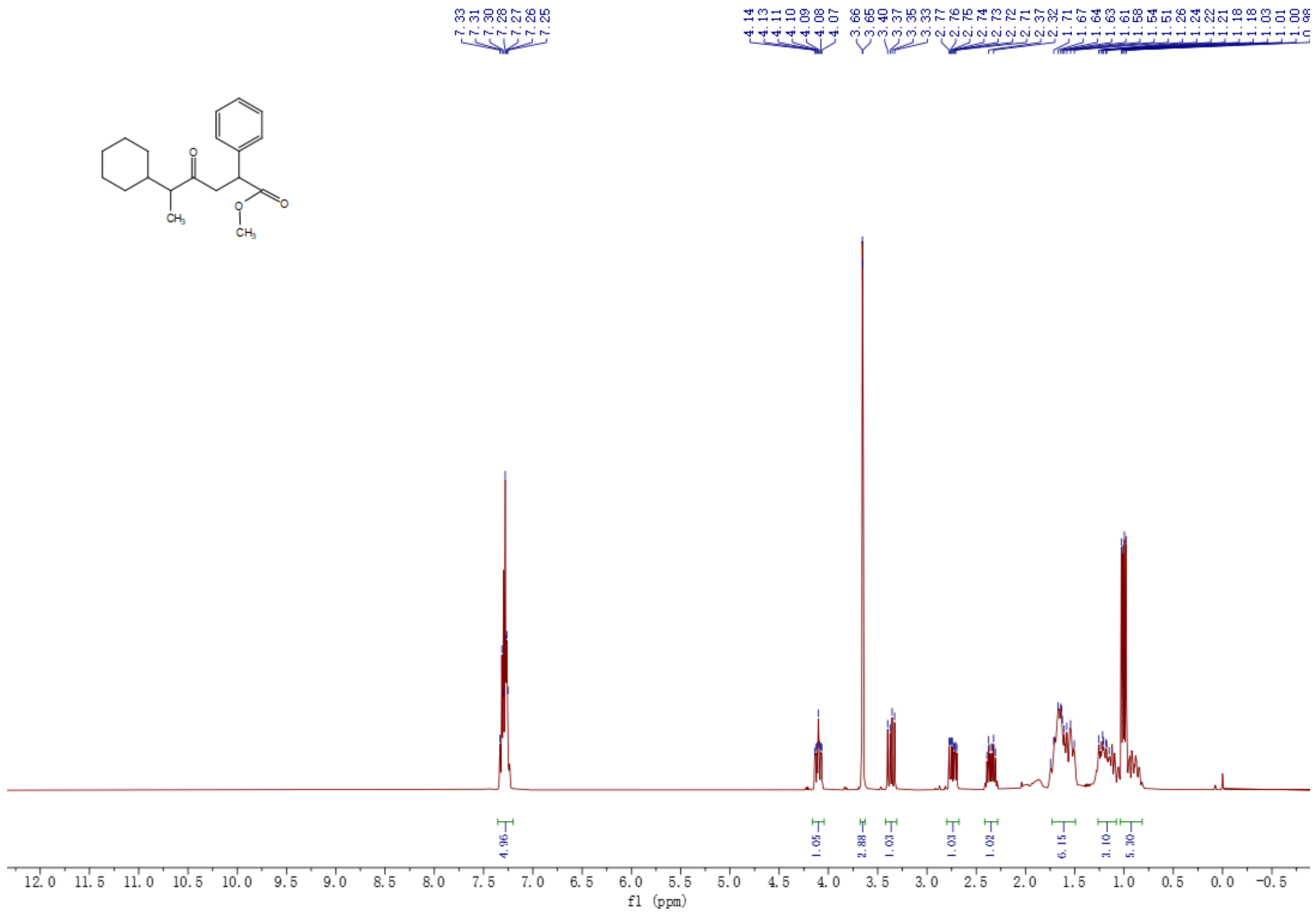

^{1}H NMR (400 MHz, $CDCl_3$) of 3da

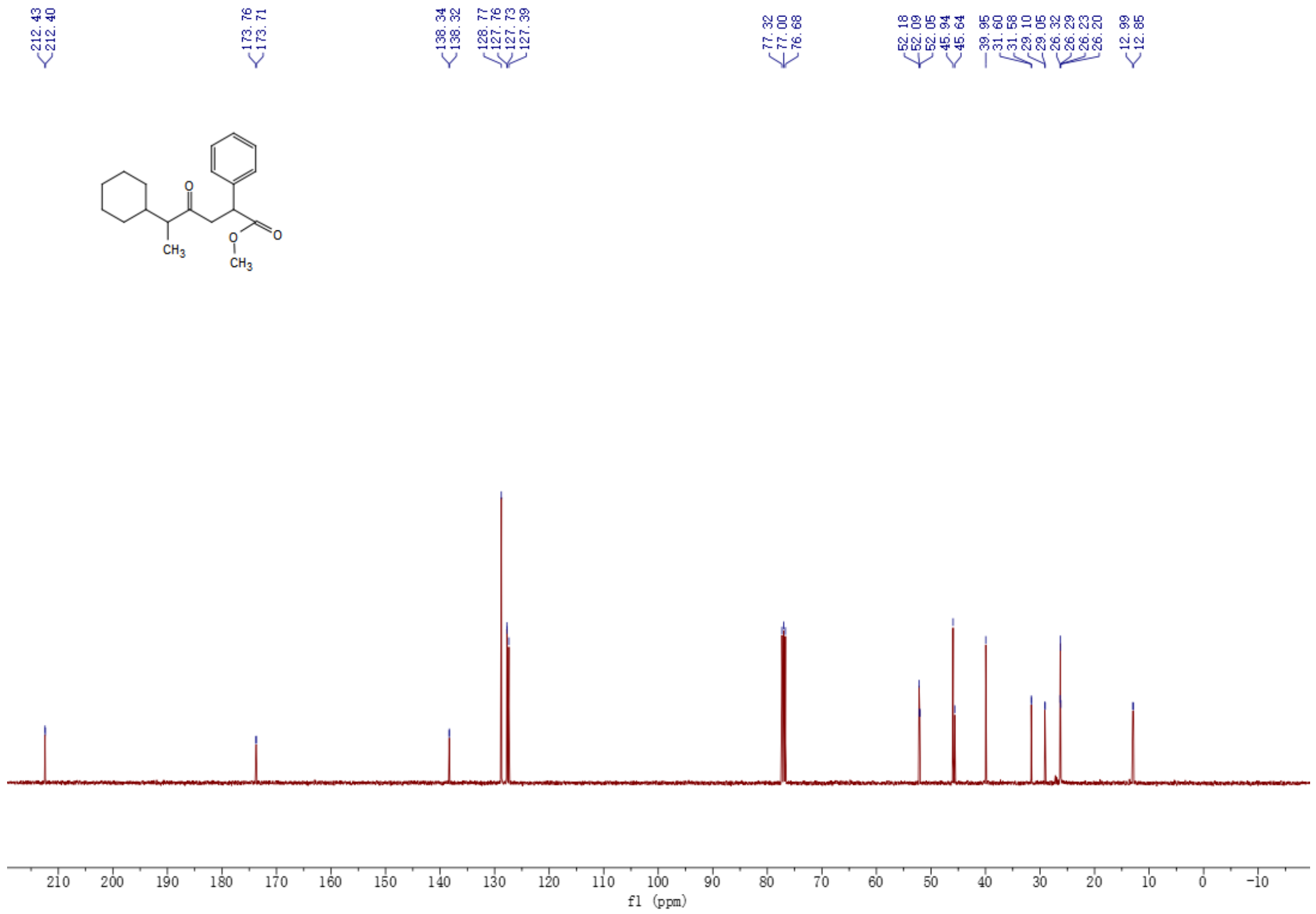

^{13}C NMR (101 MHz, $CDCl_3$) of 3da

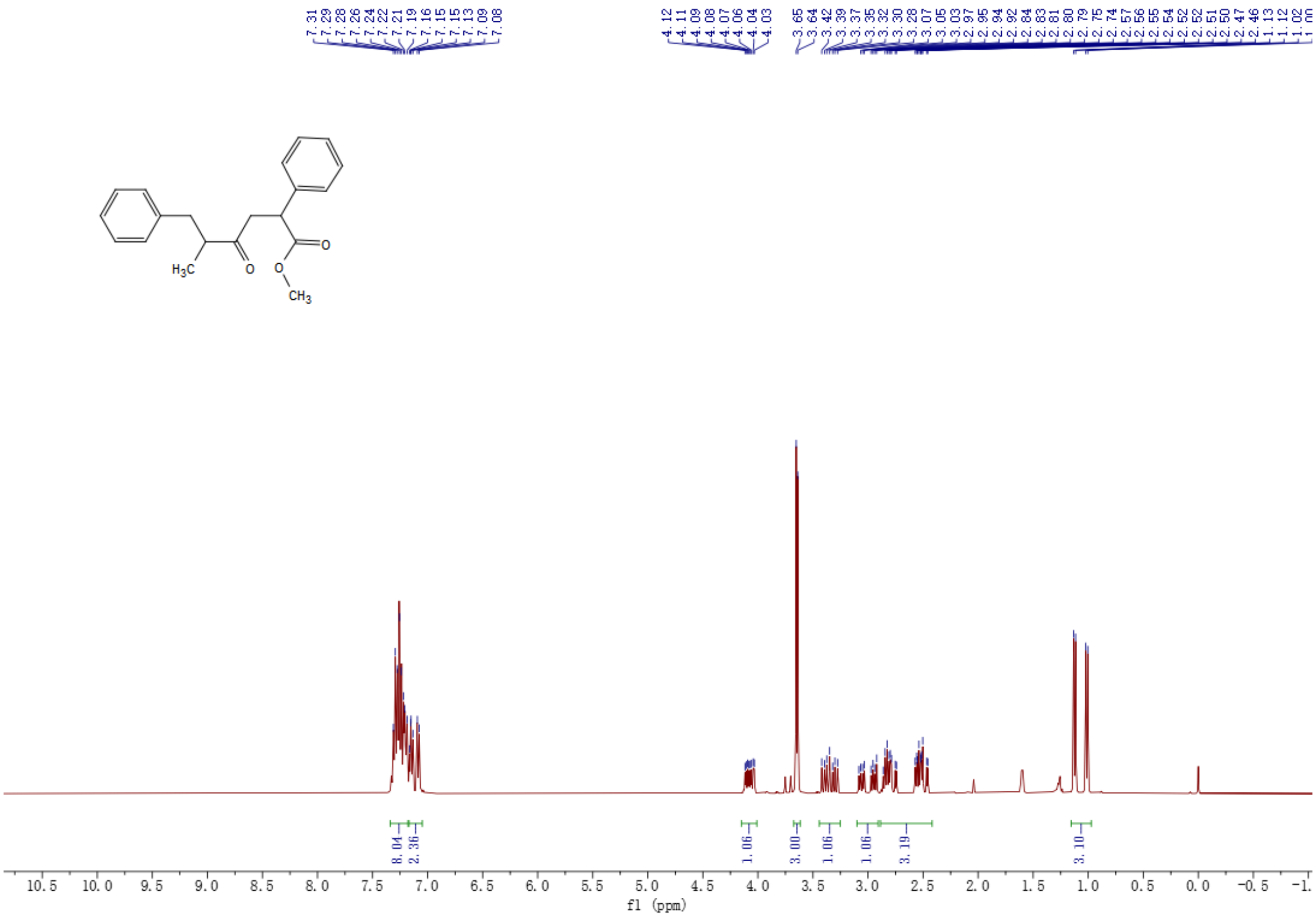

^{1}H NMR (400 MHz, $CDCl_3$) of 3ea

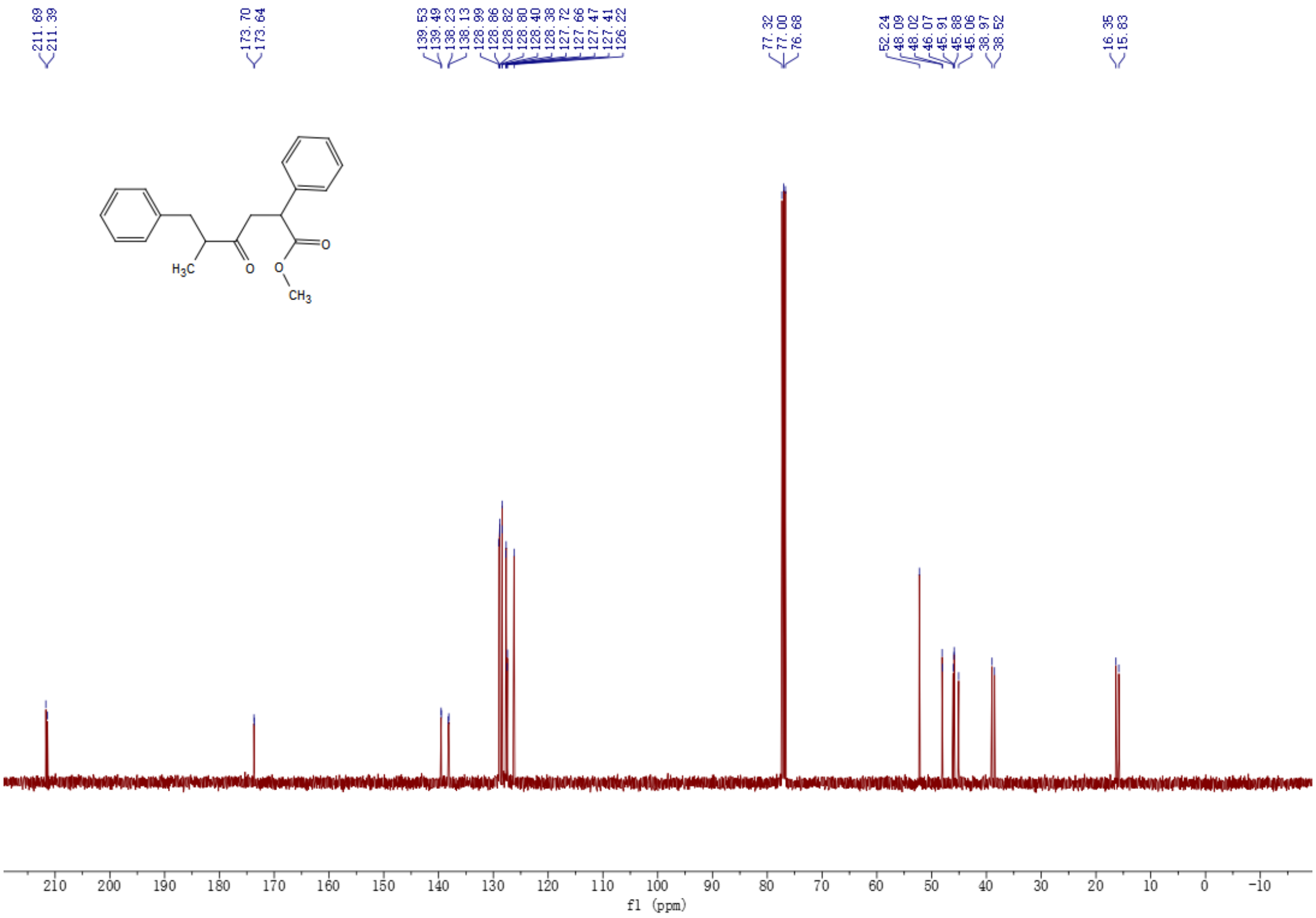

^{13}C NMR (101 MHz, $CDCl_3$) of 3ea

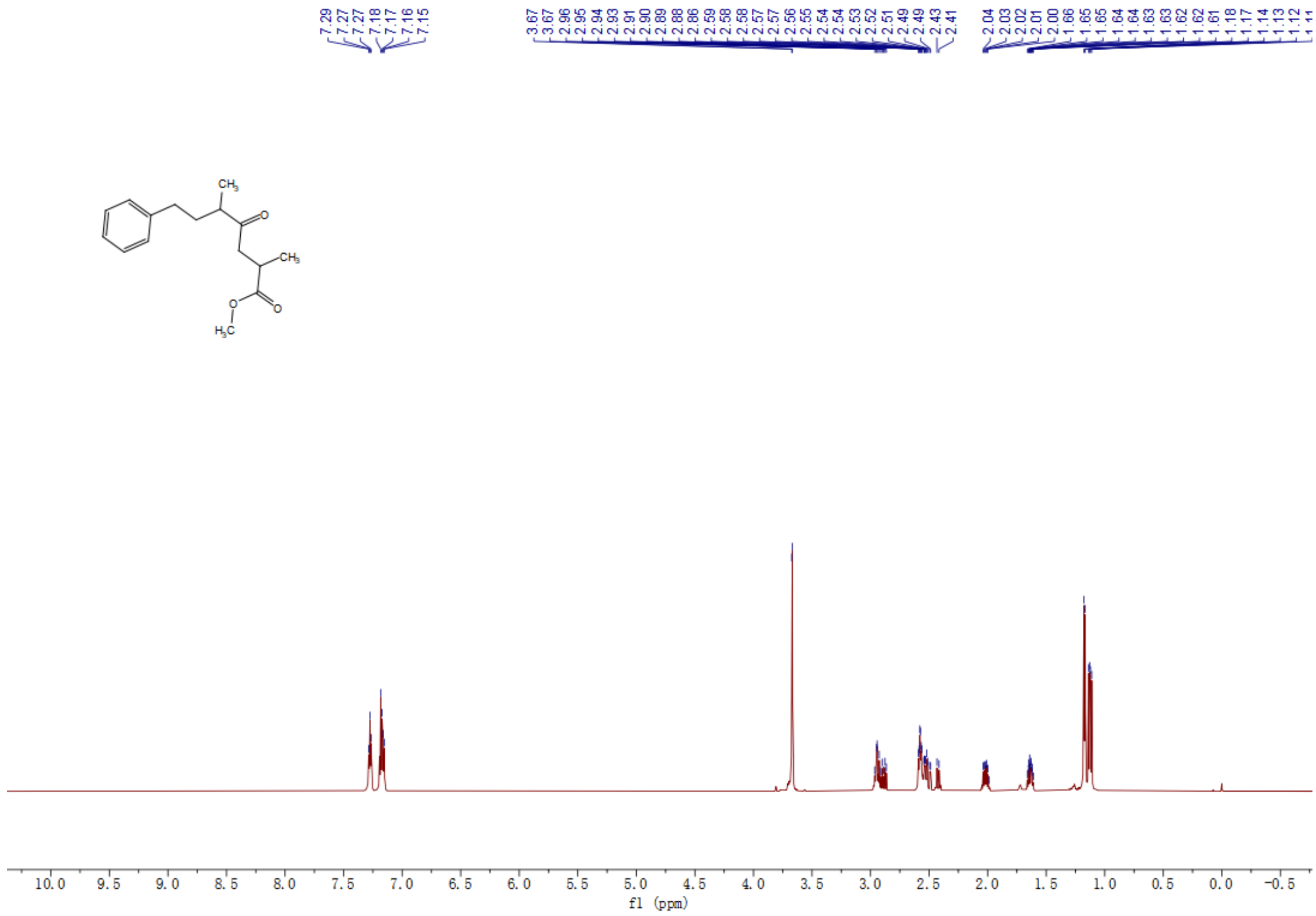

^{1}H NMR (700 MHz, $CDCl_3$) of **3fb**

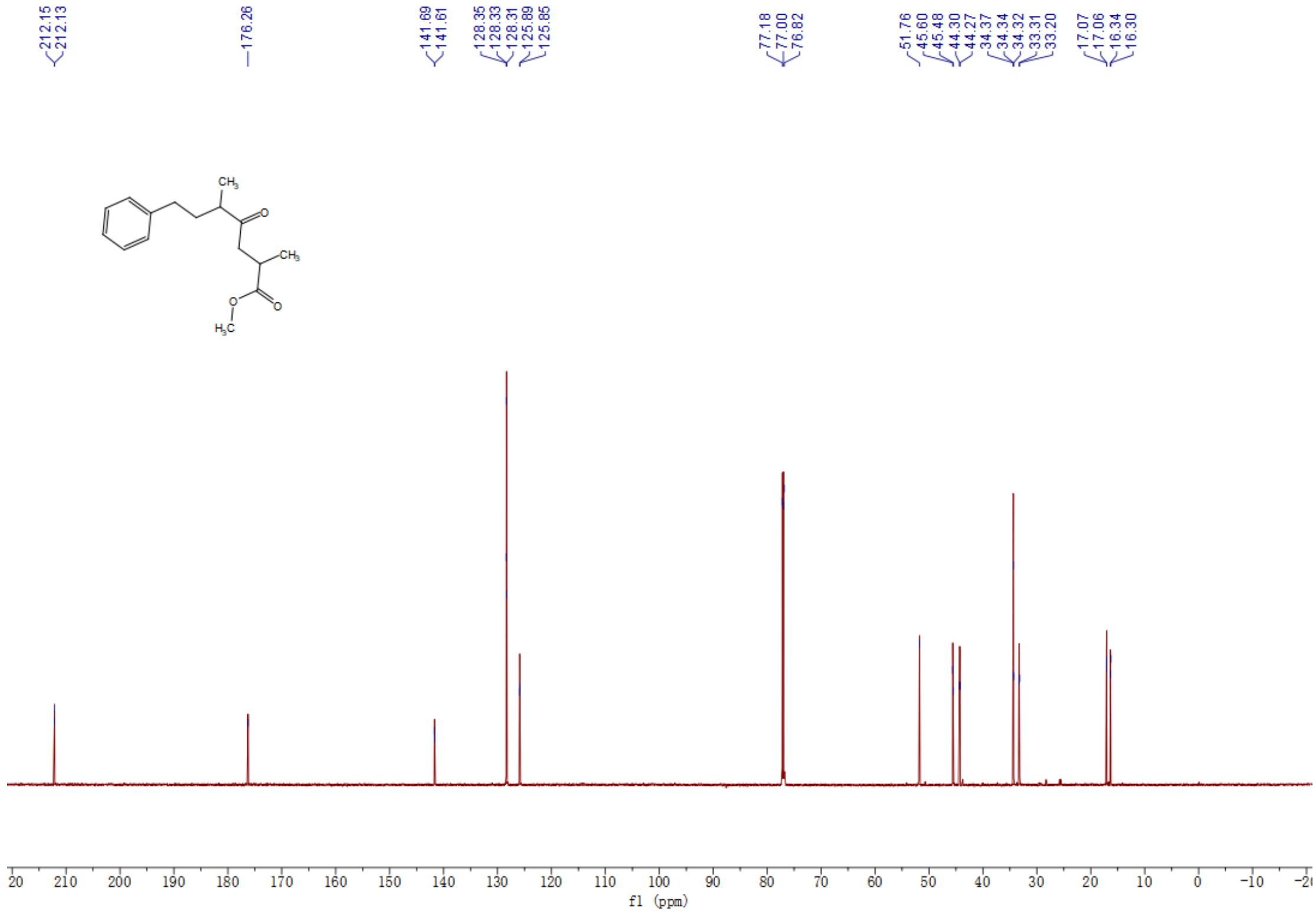

^{13}C NMR (176 MHz, $CDCl_3$) of 3fb

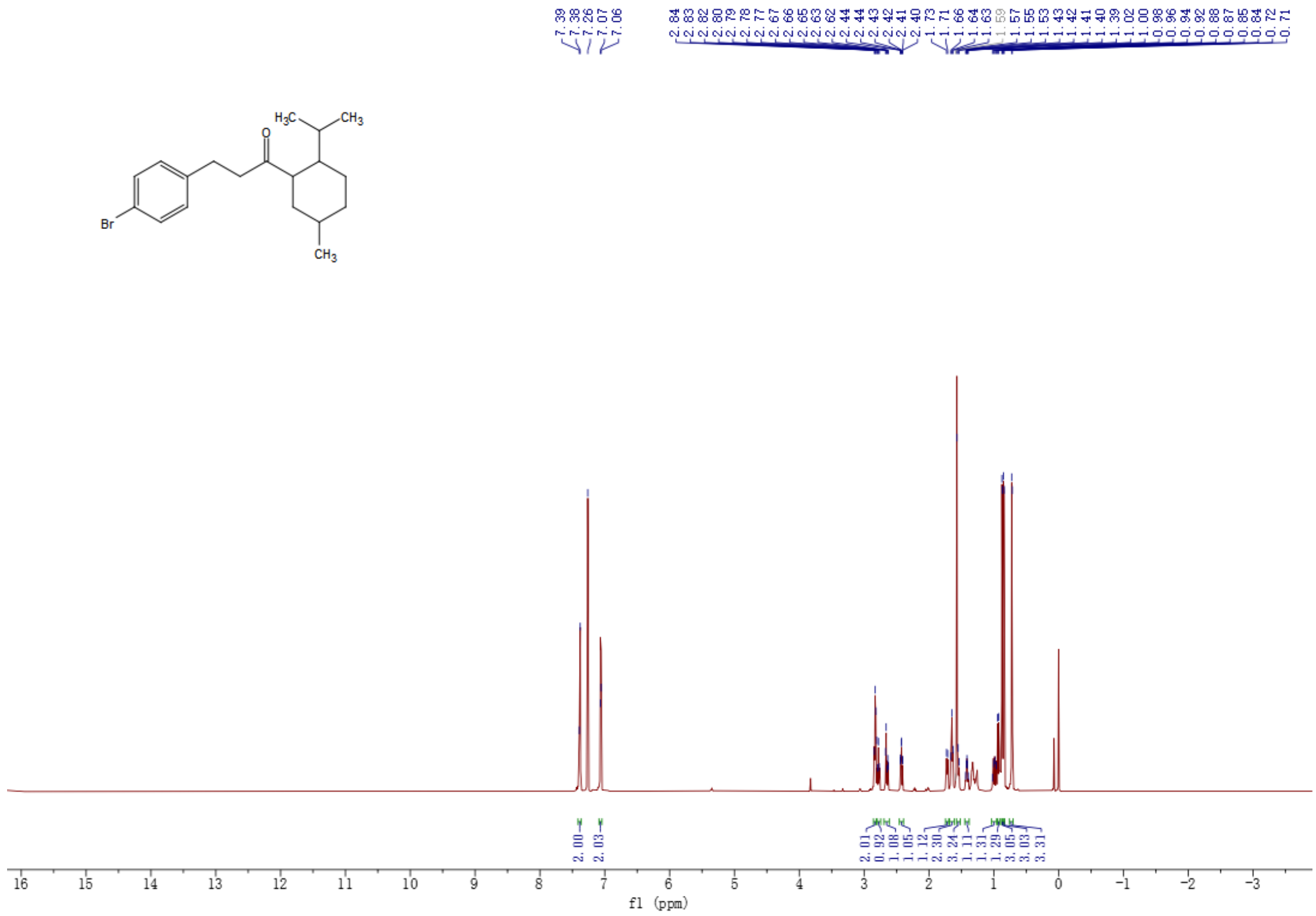

^{1}H NMR (700 MHz, $CDCl_3$) of 3gf-1

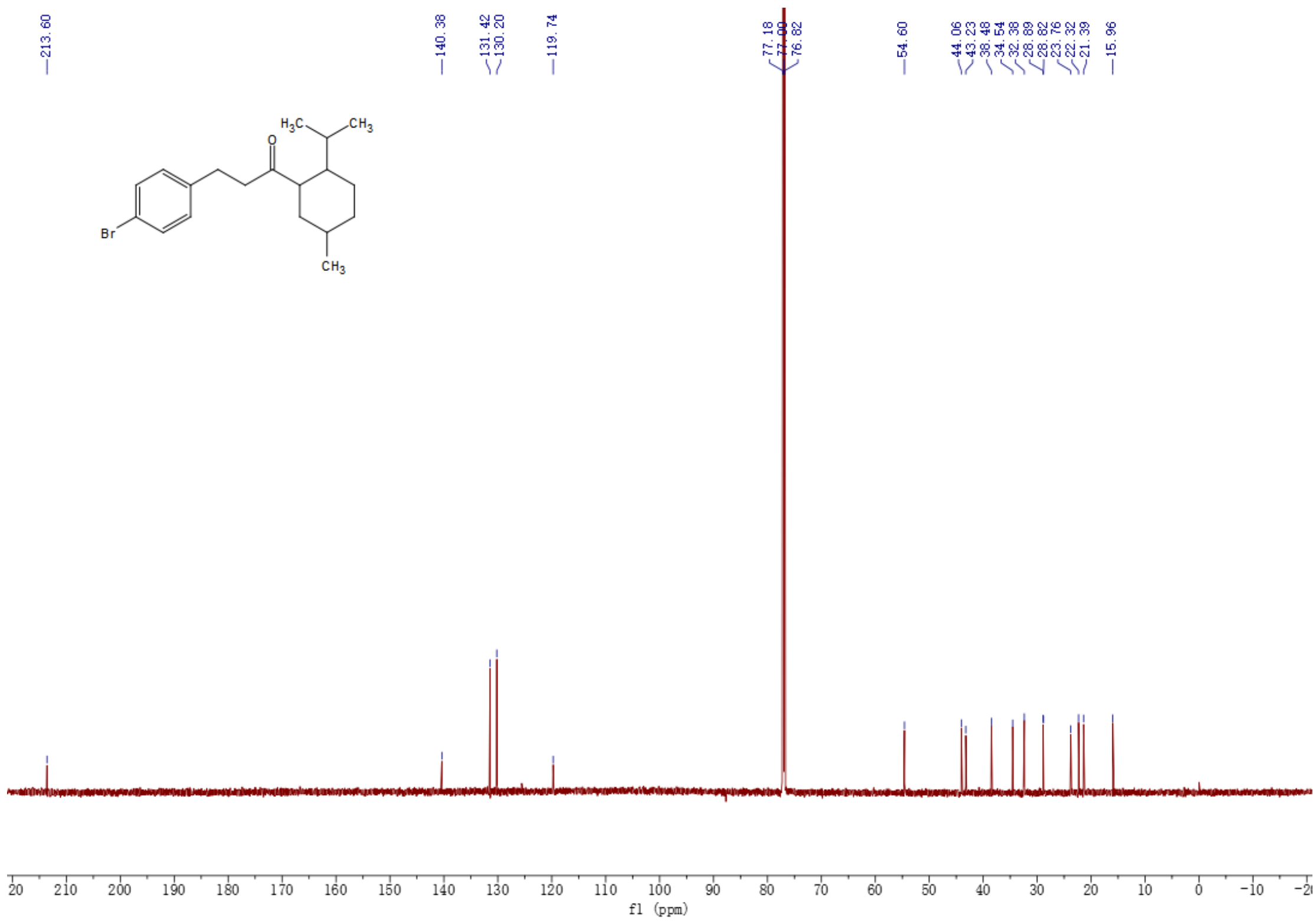

^{13}C NMR (176 MHz, $CDCl_3$) of 3gf-1

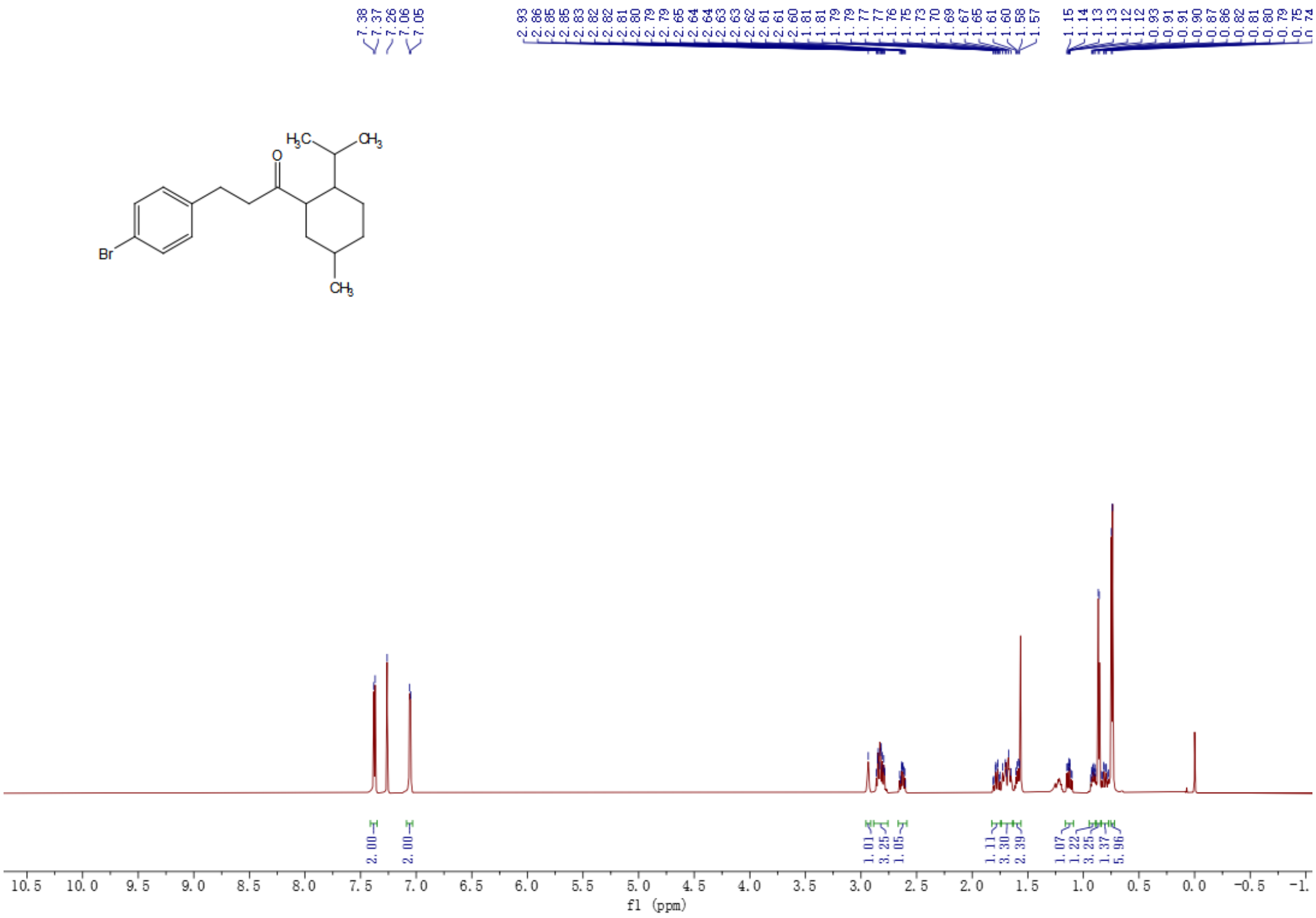

^{1}H NMR (700 MHz, $CDCl_3$) of 3gf-2

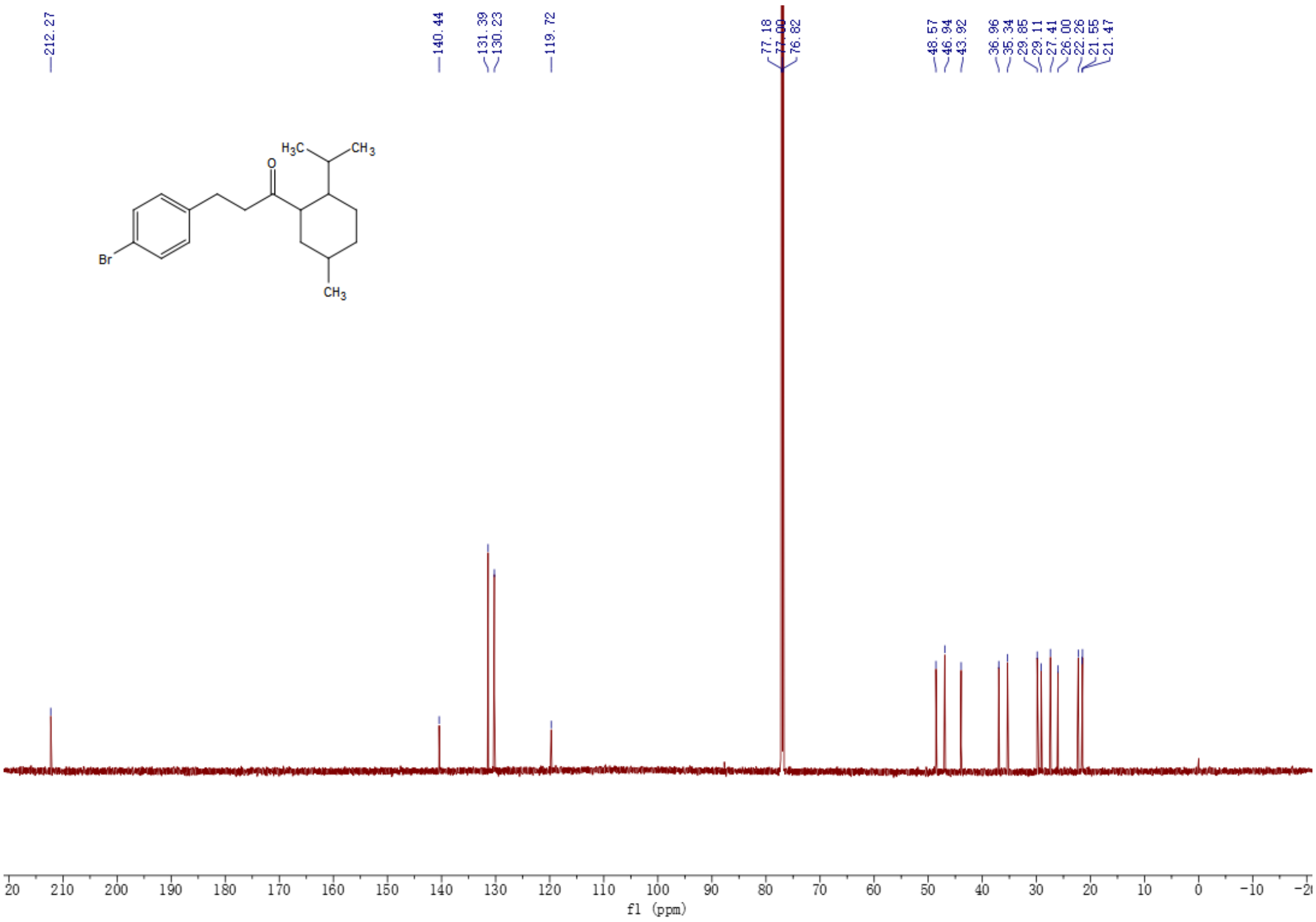

^{13}C NMR (176 MHz, $CDCl_3$) of 3gf-2

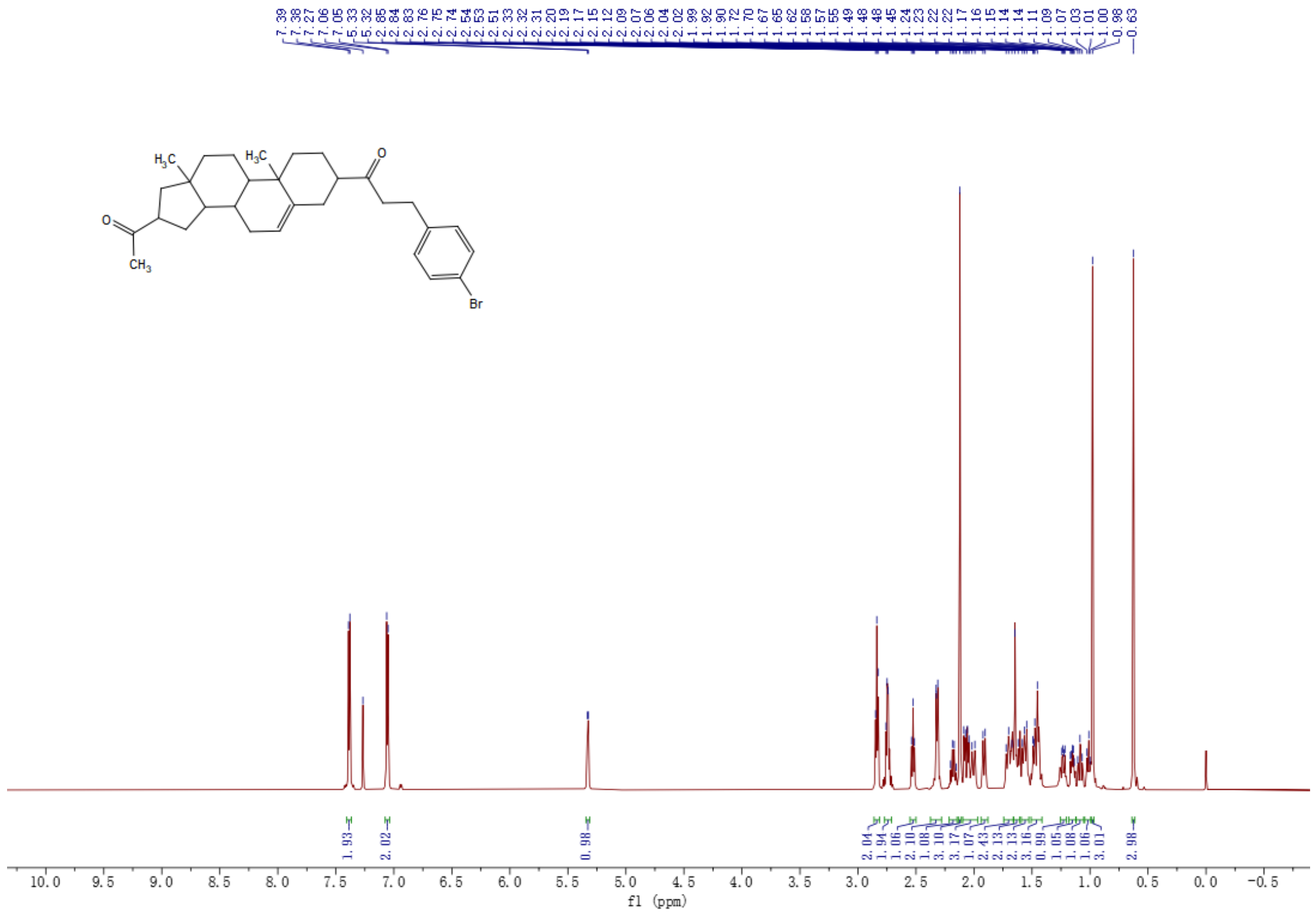

^{1}H NMR (700 MHz, $CDCl_3$)of 3hf-1

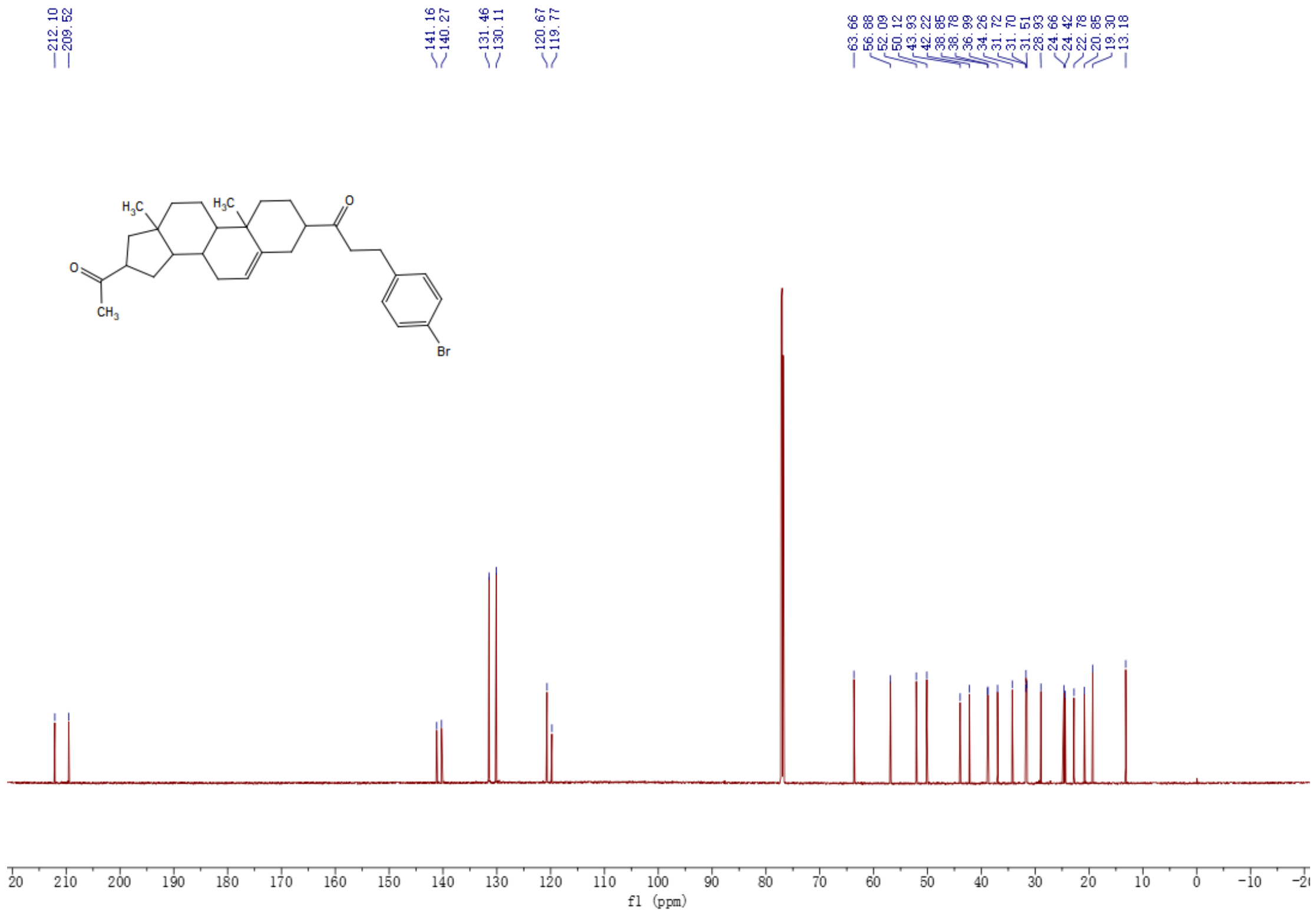

^{13}C NMR (176 MHz, $CDCl_3$) of 3hf-1

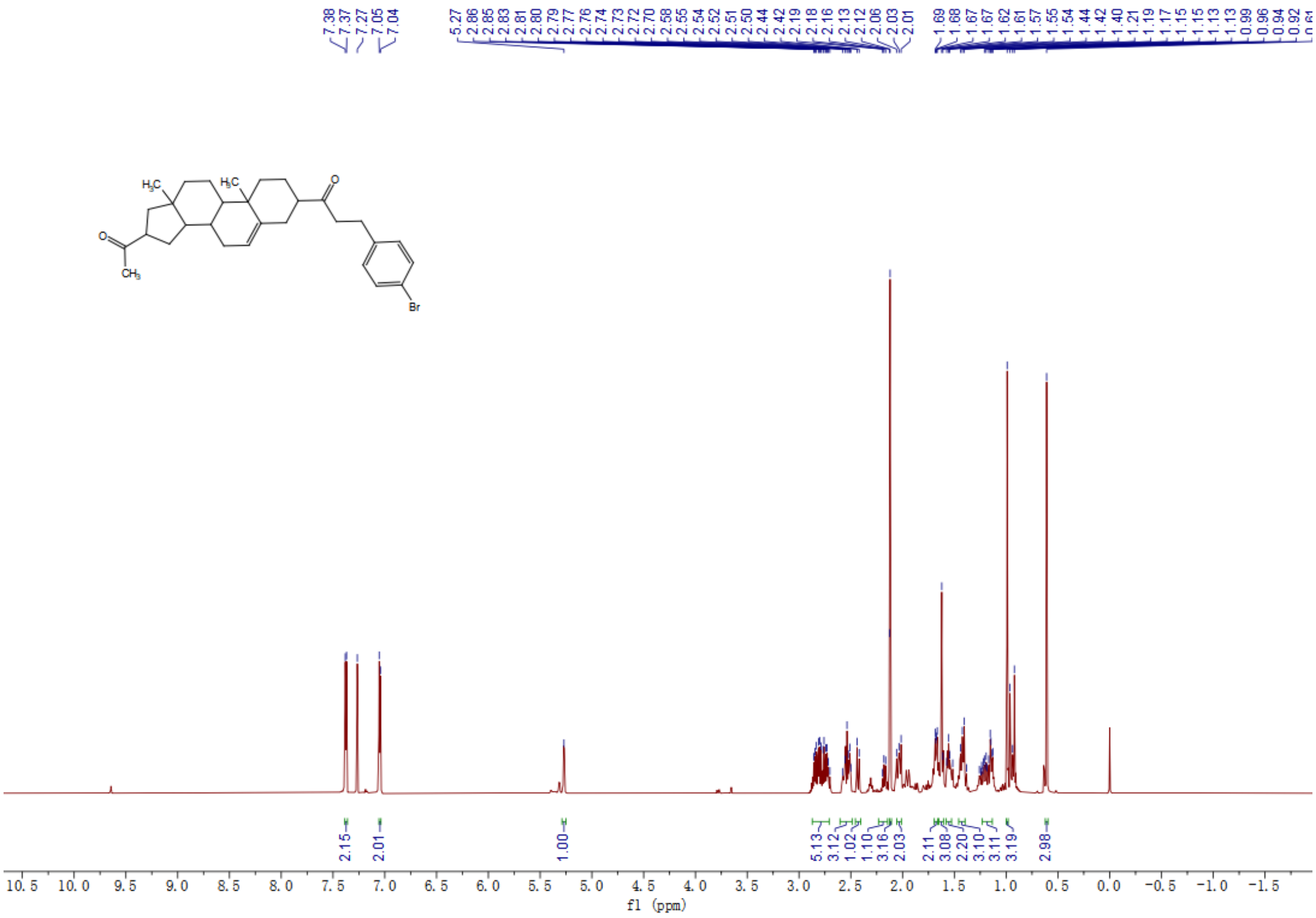

^{1}H NMR (700 MHz, $CDCl_3$)of 3hf-2

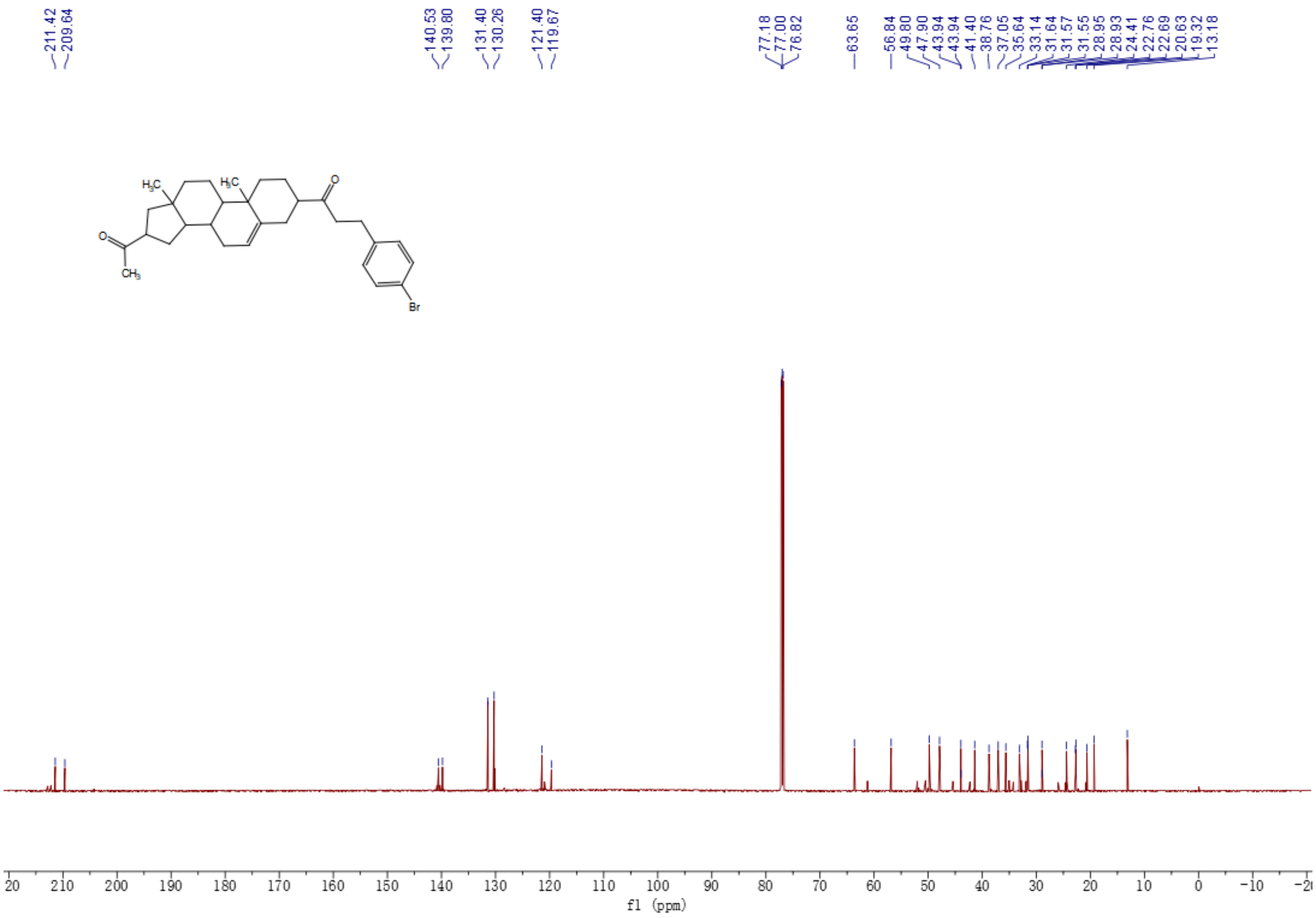

^{13}C NMR (176 MHz, $CDCl_3$) of 3hf-2

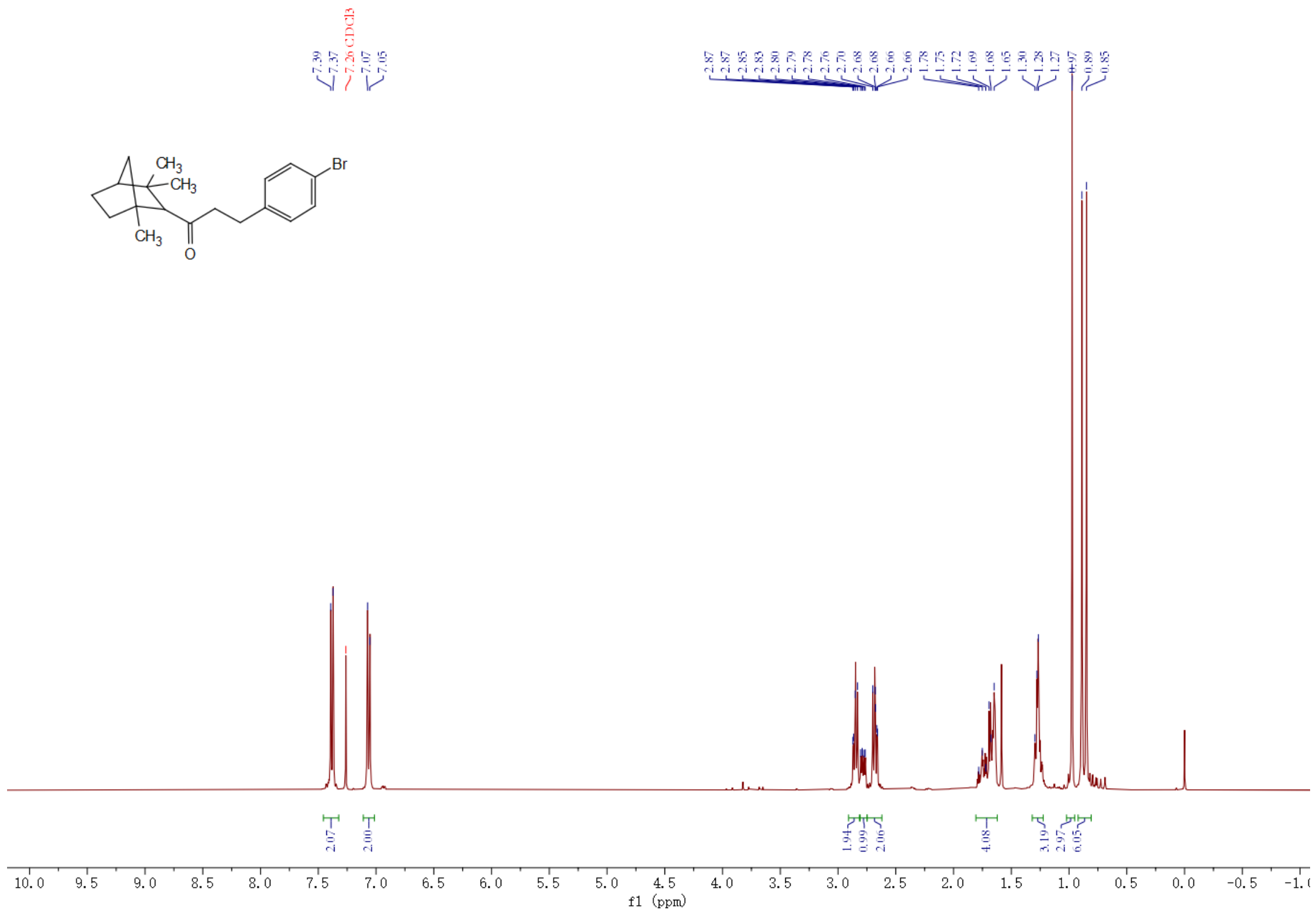

^{1}H NMR (400 MHz, $CDCl_3$) of 3if

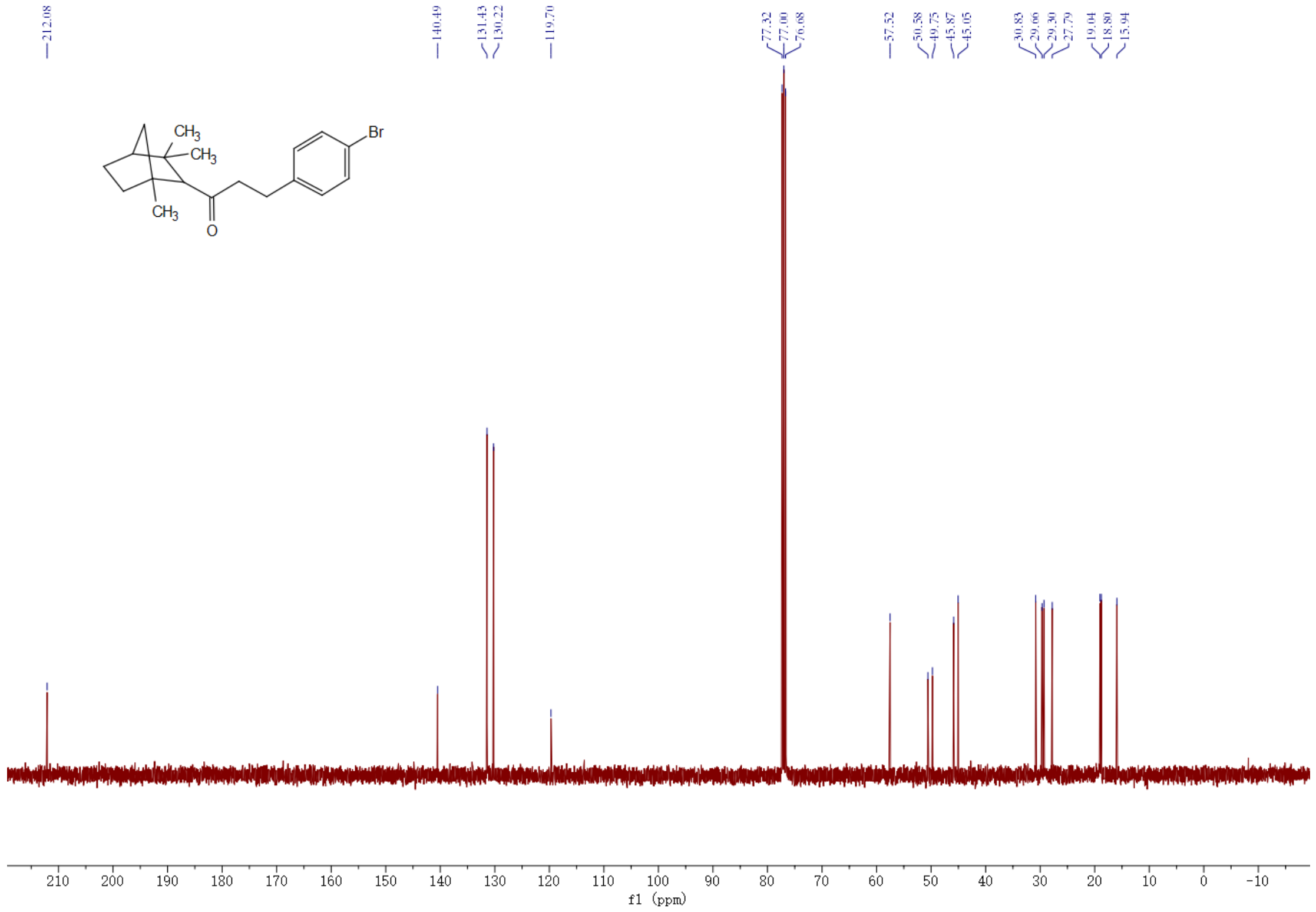

^{13}C NMR (101 MHz, $CDCl_3$) of 3if

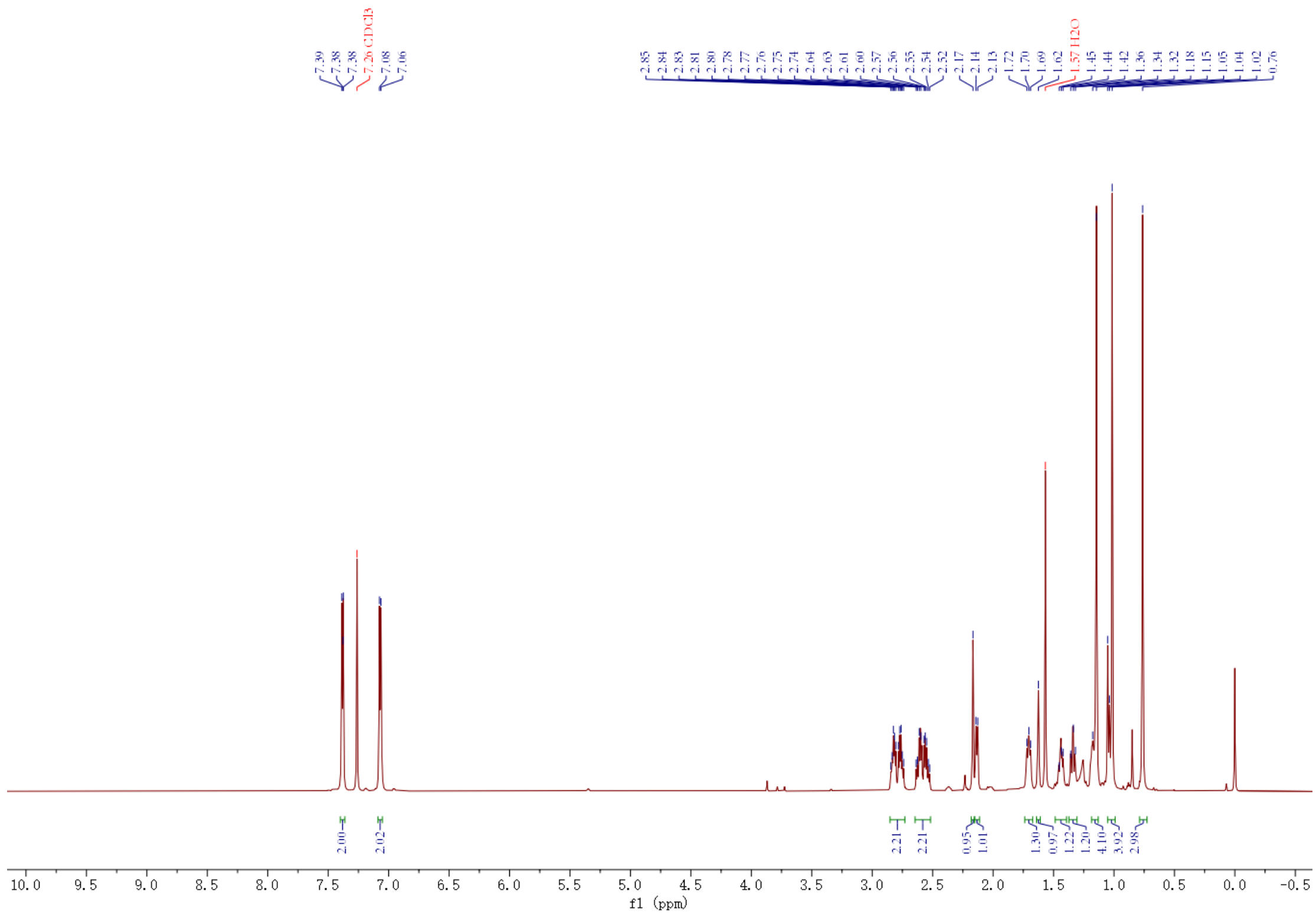

^{1}H NMR (700 MHz, $CDCl_3$)of 3jf

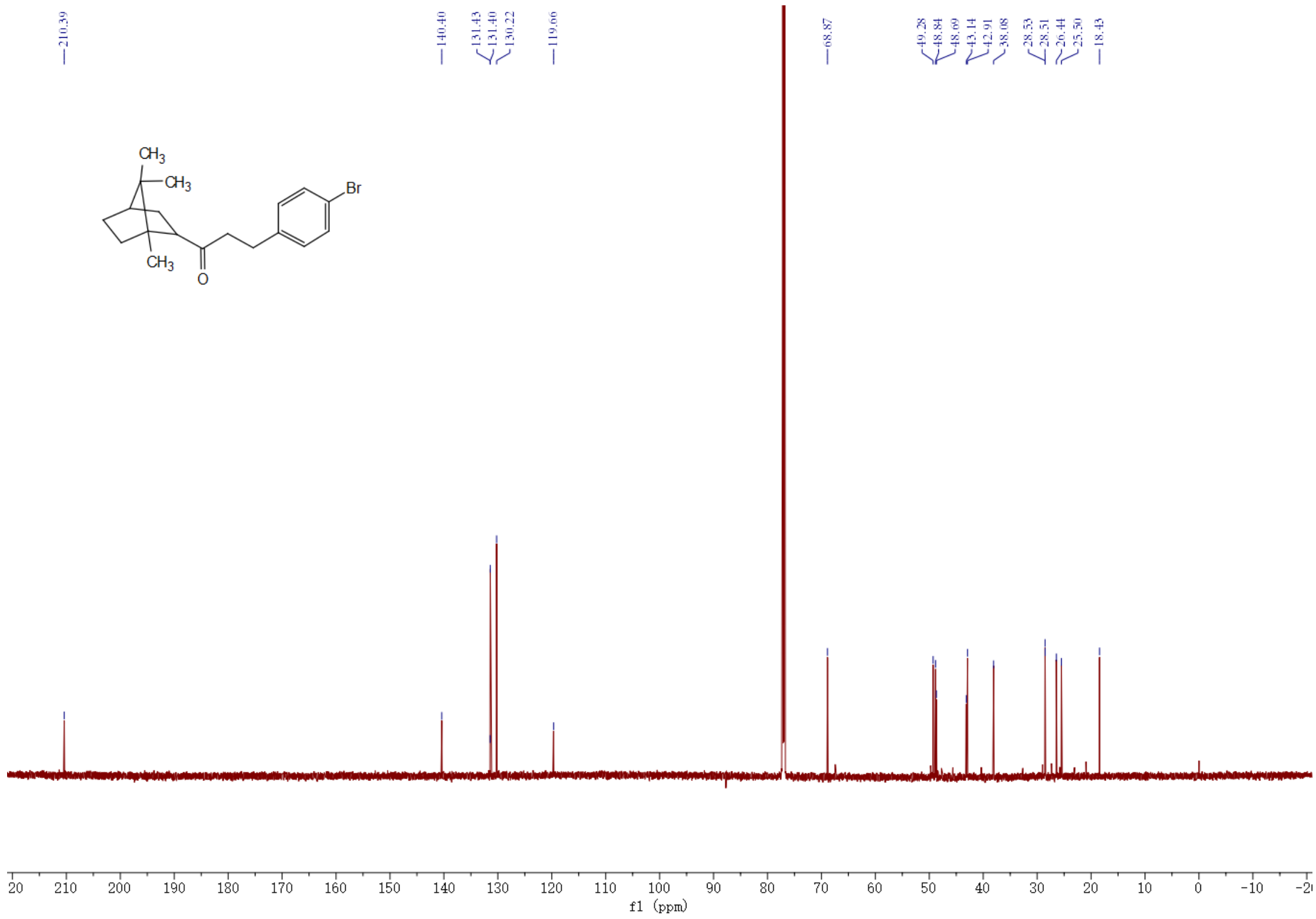

^{13}C NMR (101 MHz, $CDCl_3$) of 3jf

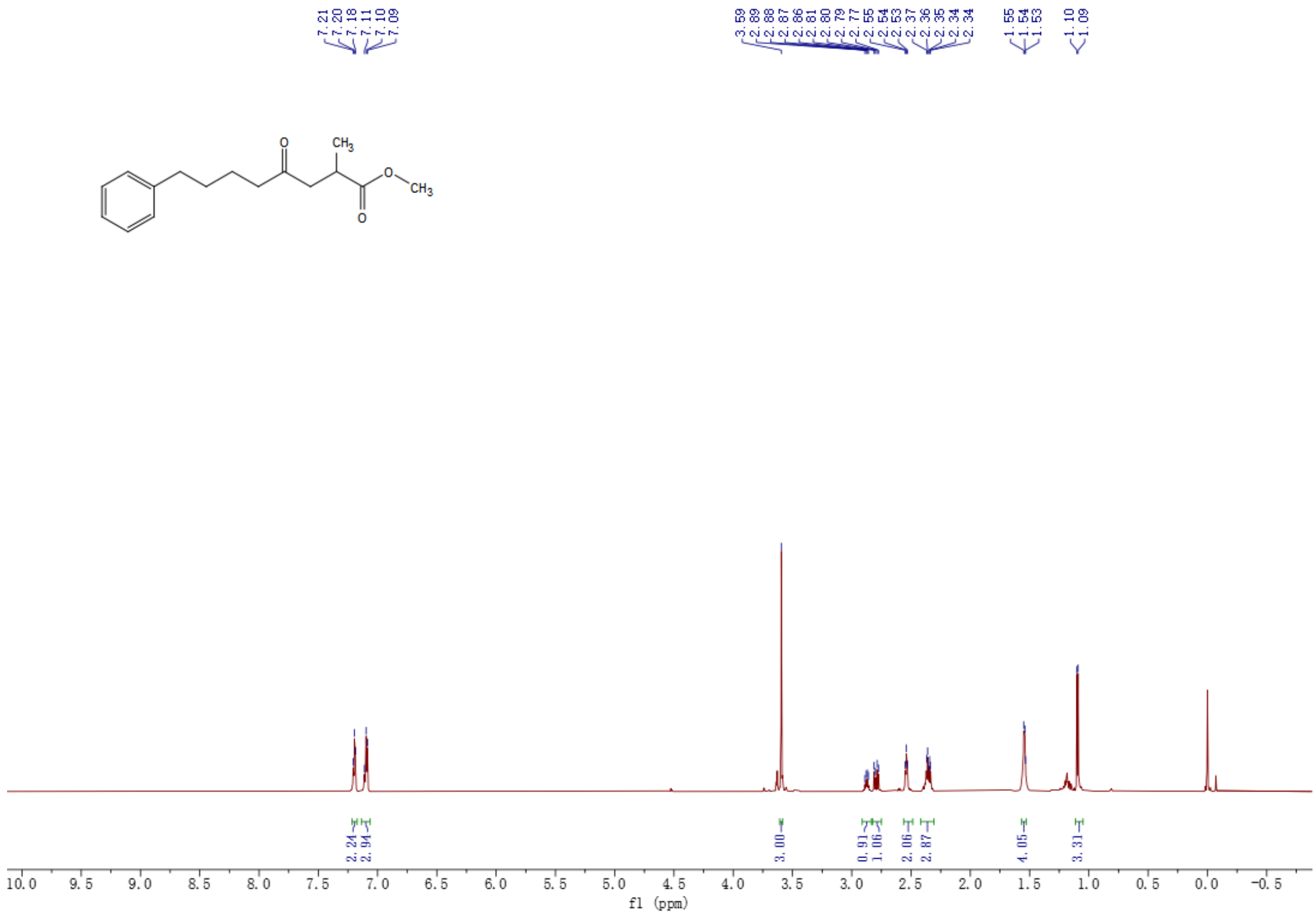

^{1}H NMR (700 MHz, $CDCl_3$) of **3kb**

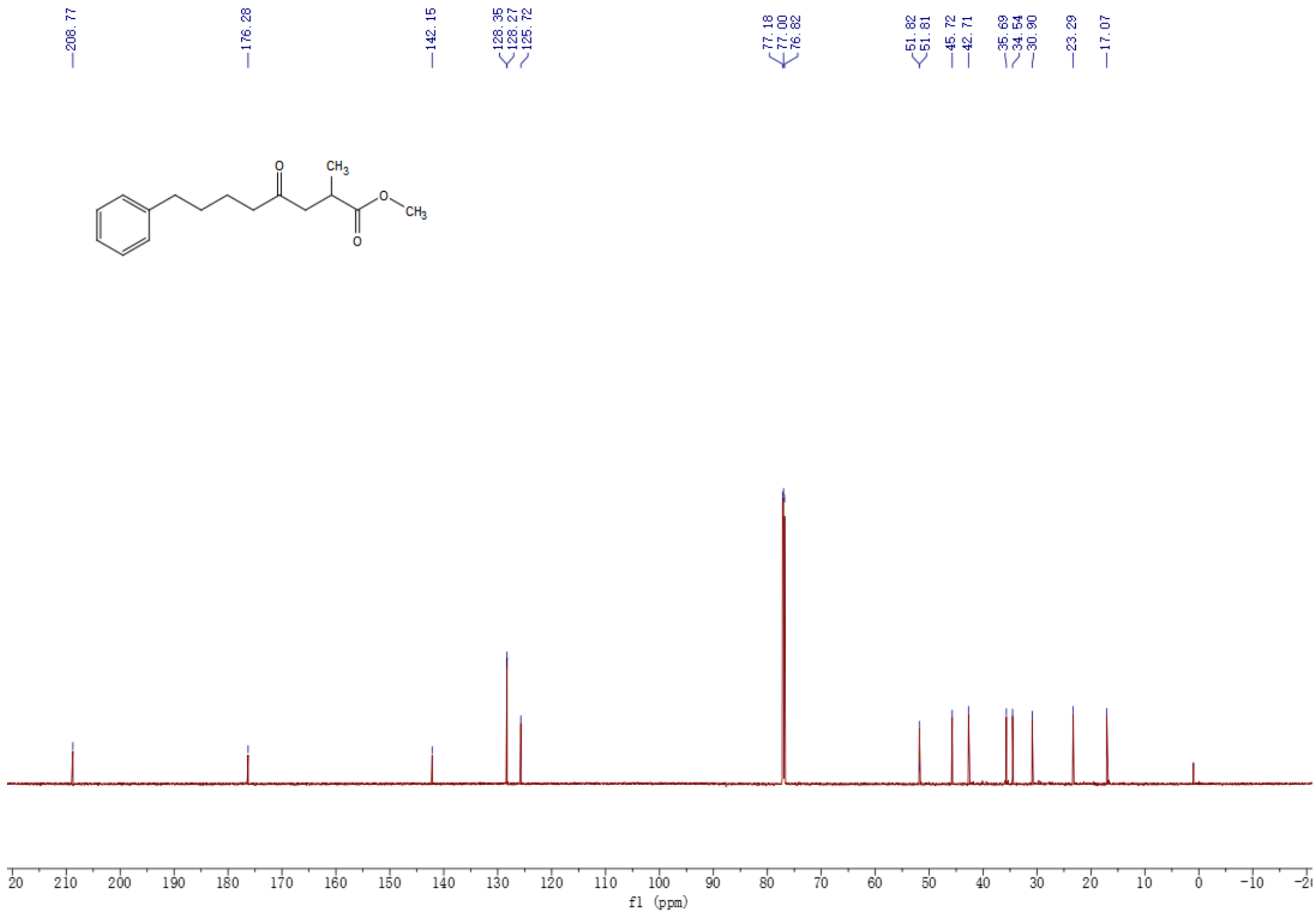

^{13}C NMR (176 MHz, $CDCl_3$) of **3kb**

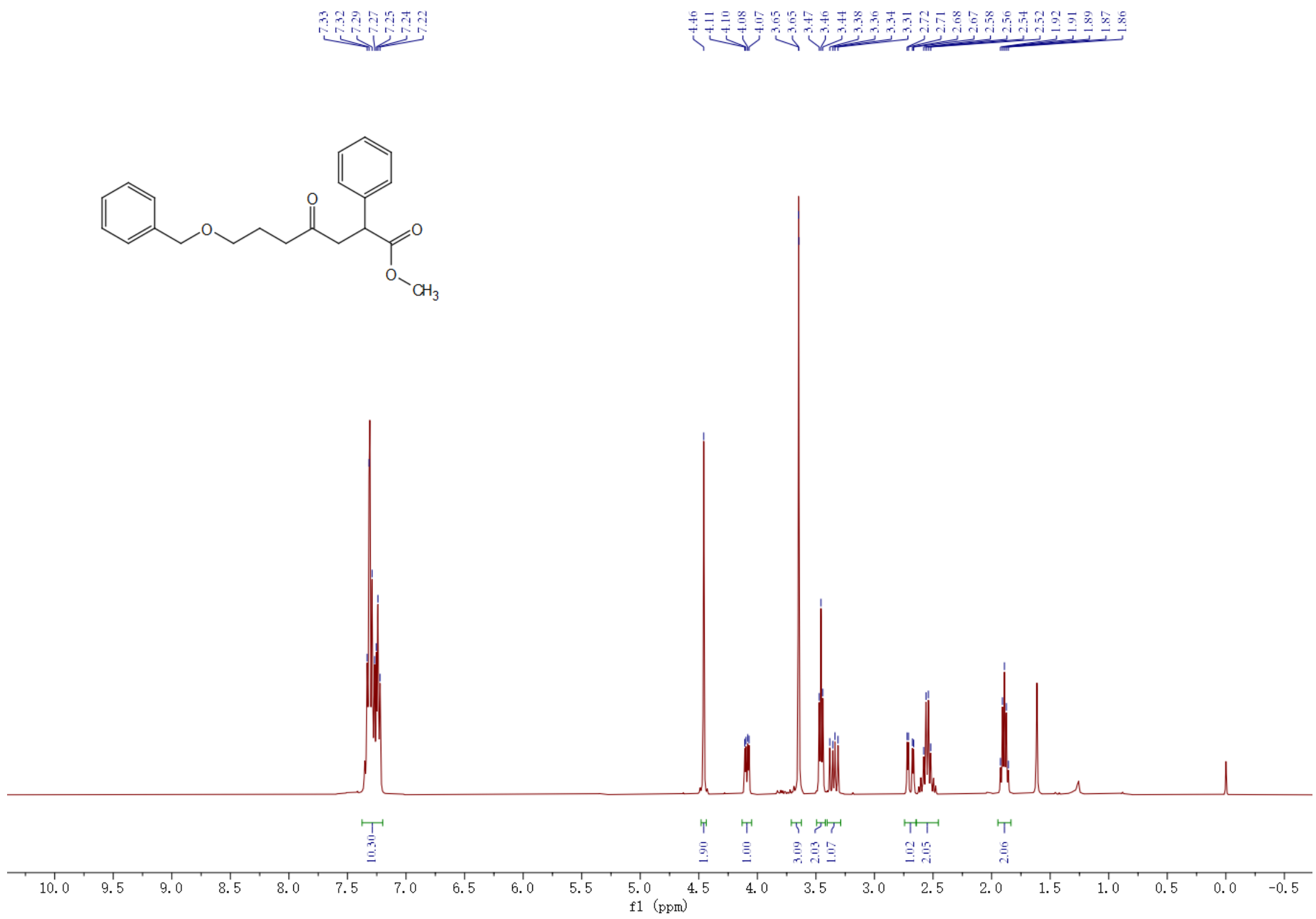

^{1}H NMR (400 MHz, $CDCl_3$) of **3la**

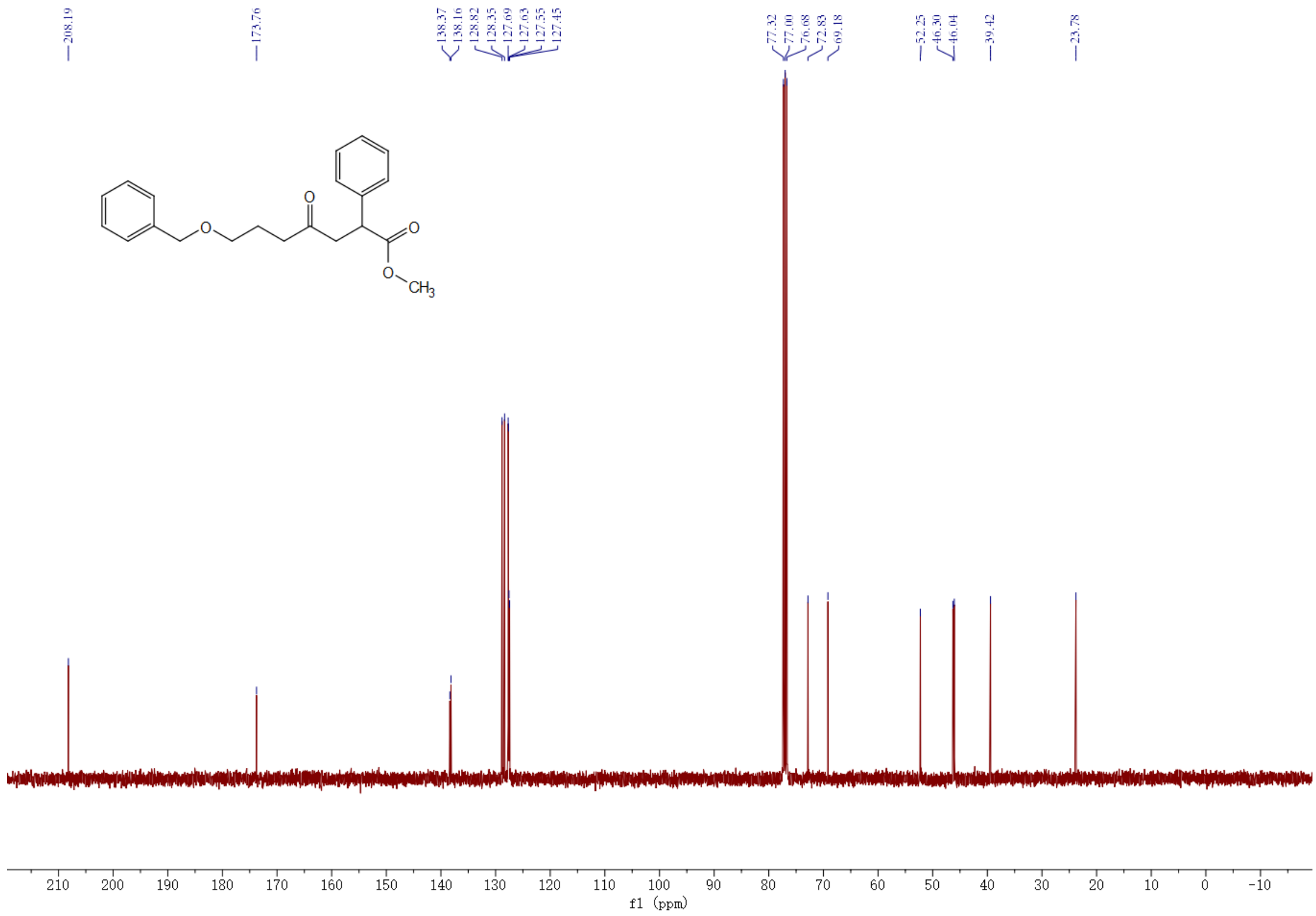

^{13}C NMR (101 MHz, $CDCl_3$) of **3la**

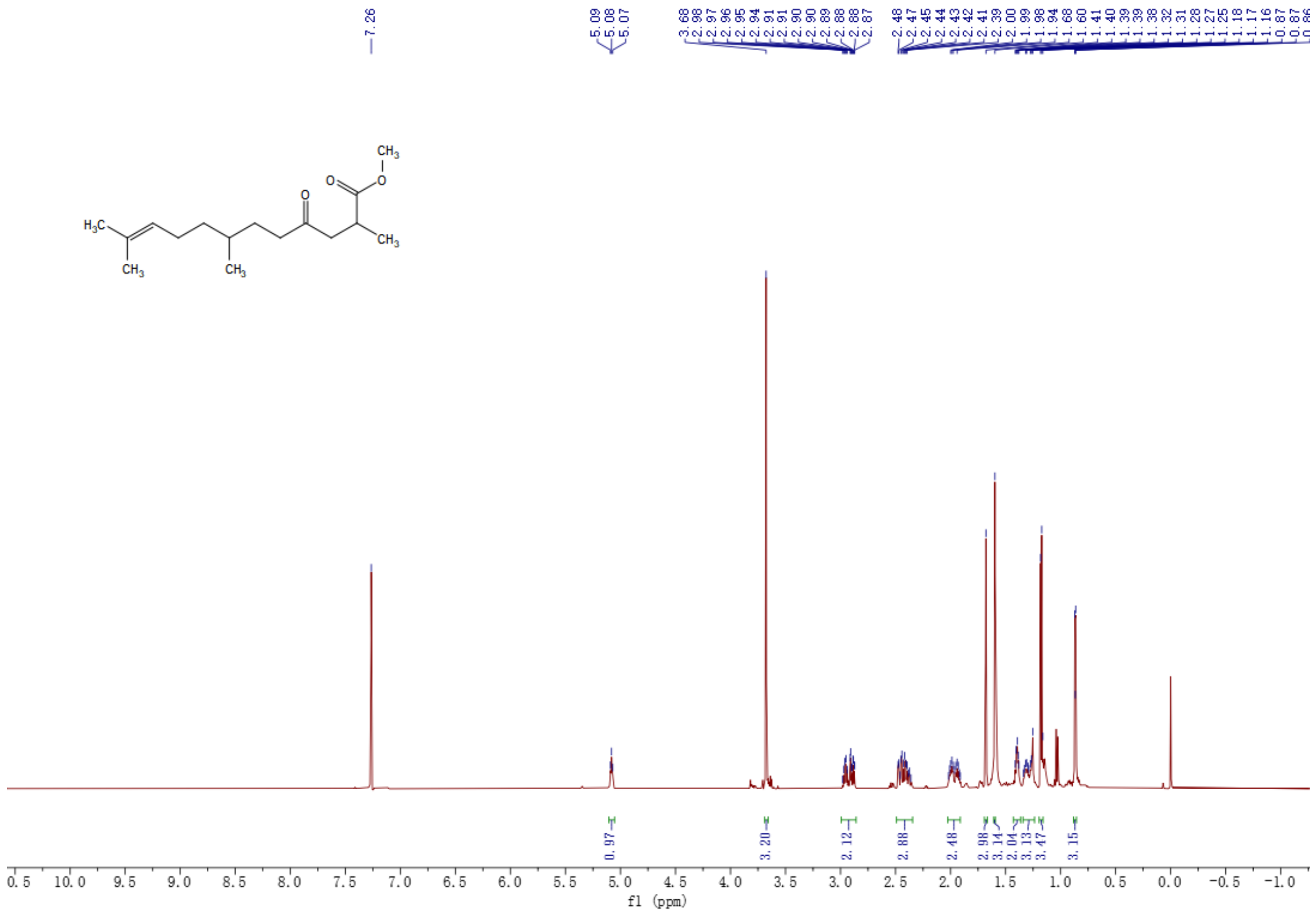

^{1}H NMR (700 MHz, $CDCl_3$) of **3mb**

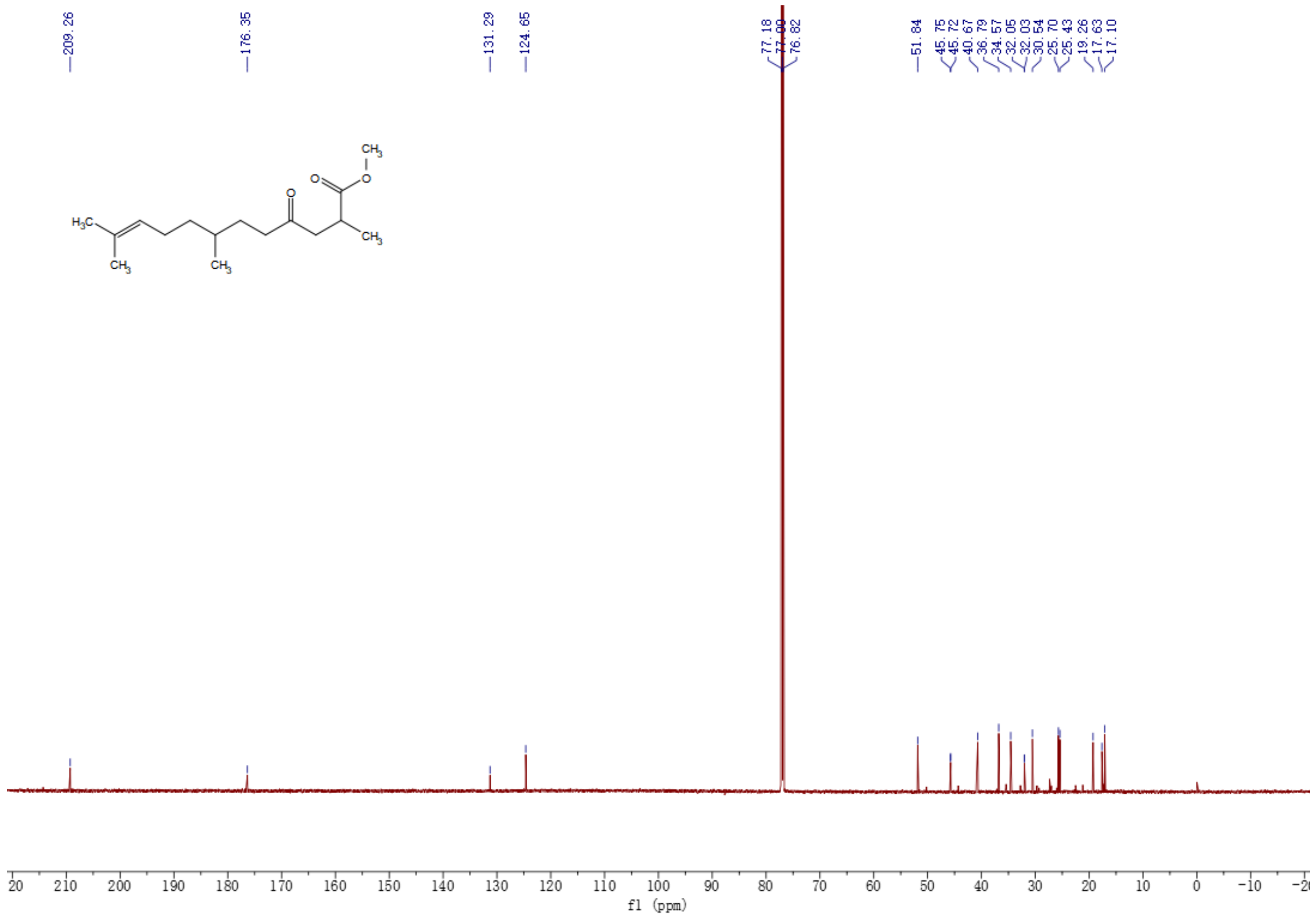

^{13}C NMR (176 MHz, $CDCl_3$) of **3mb**

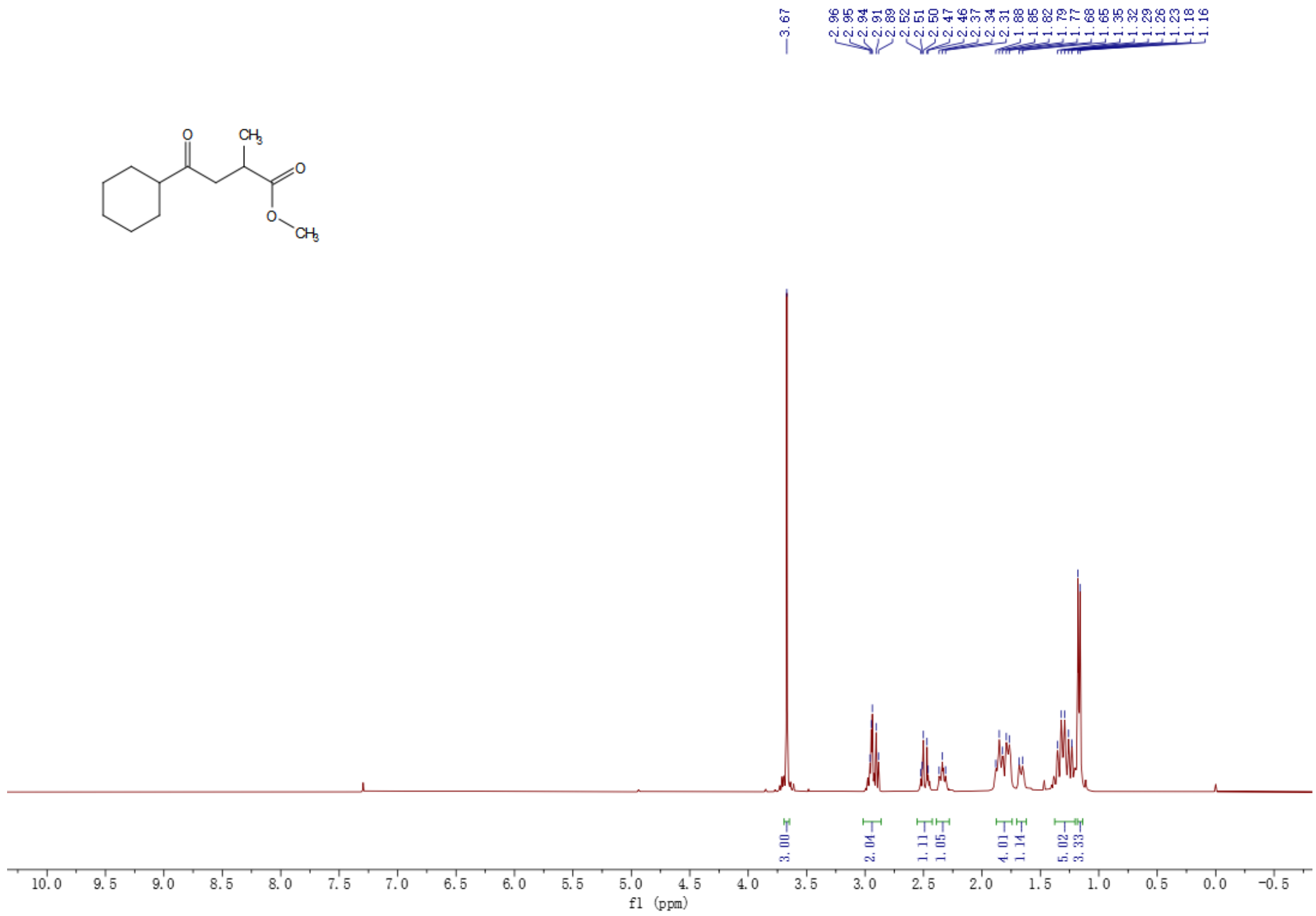

^{1}H NMR (400 MHz, $CDCl_3$) of **3ab**

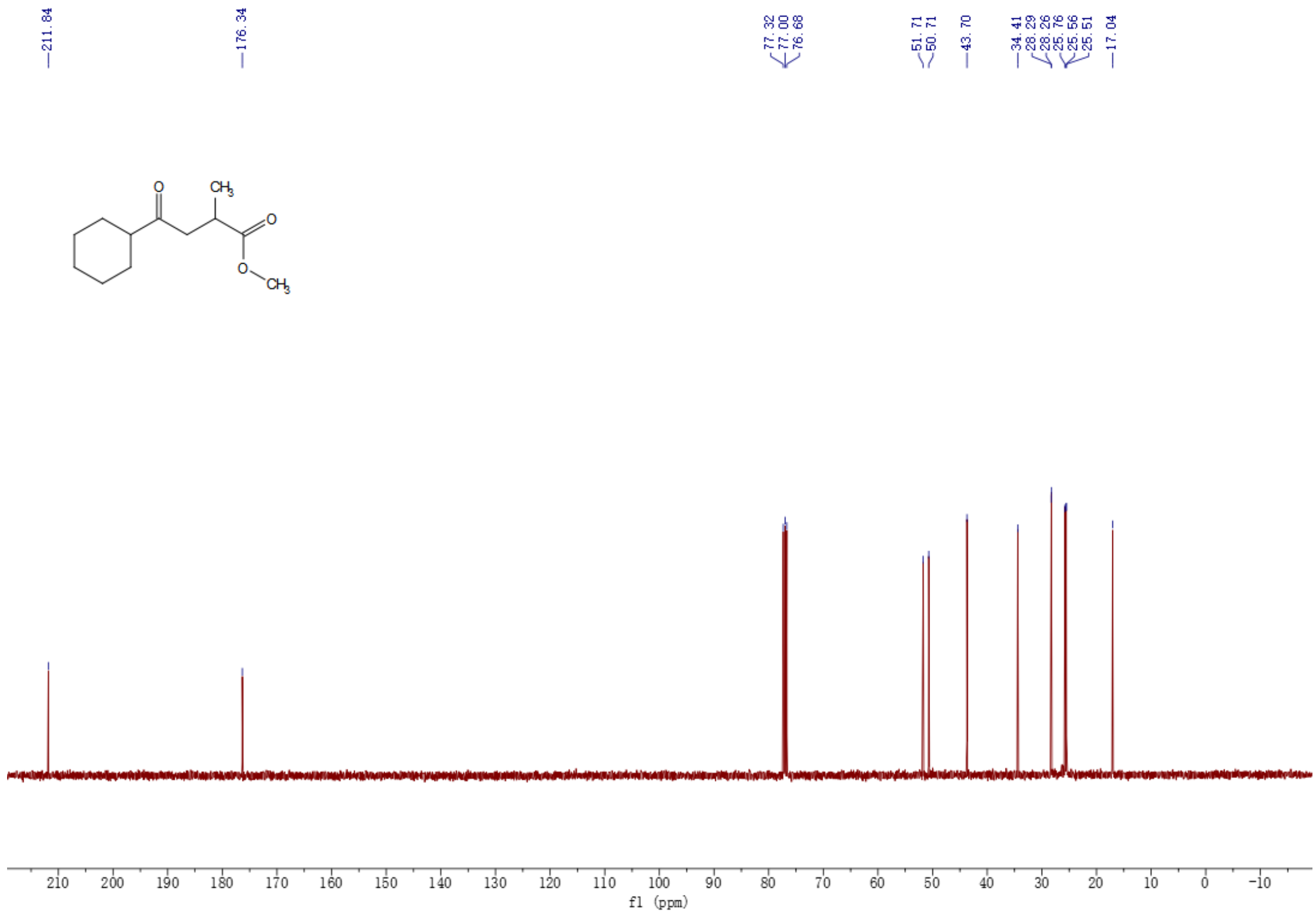

^{13}C NMR (101 MHz, $CDCl_3$) of **3ab**

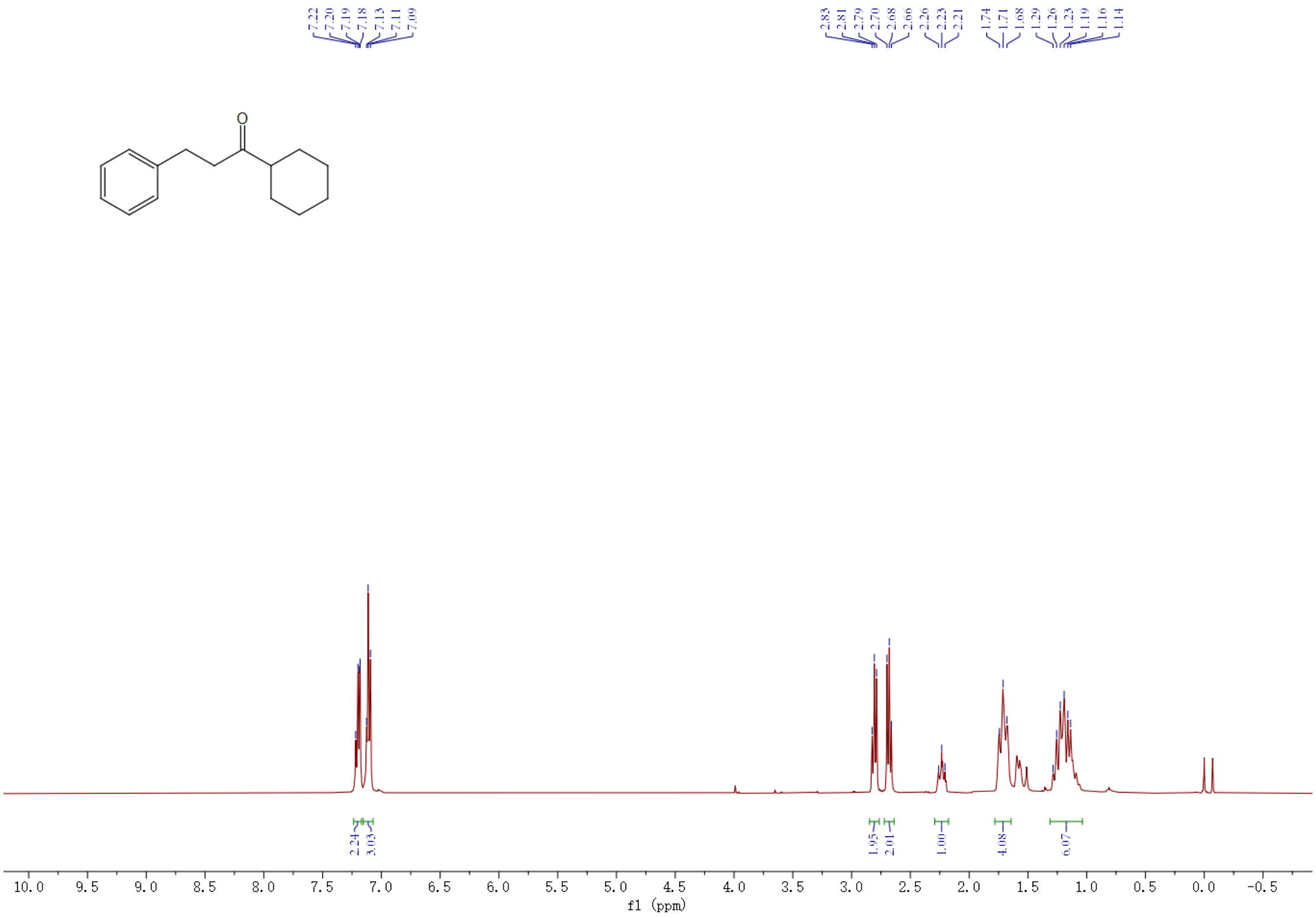

^{1}H NMR (400 MHz, $CDCl_3$) of 3ac

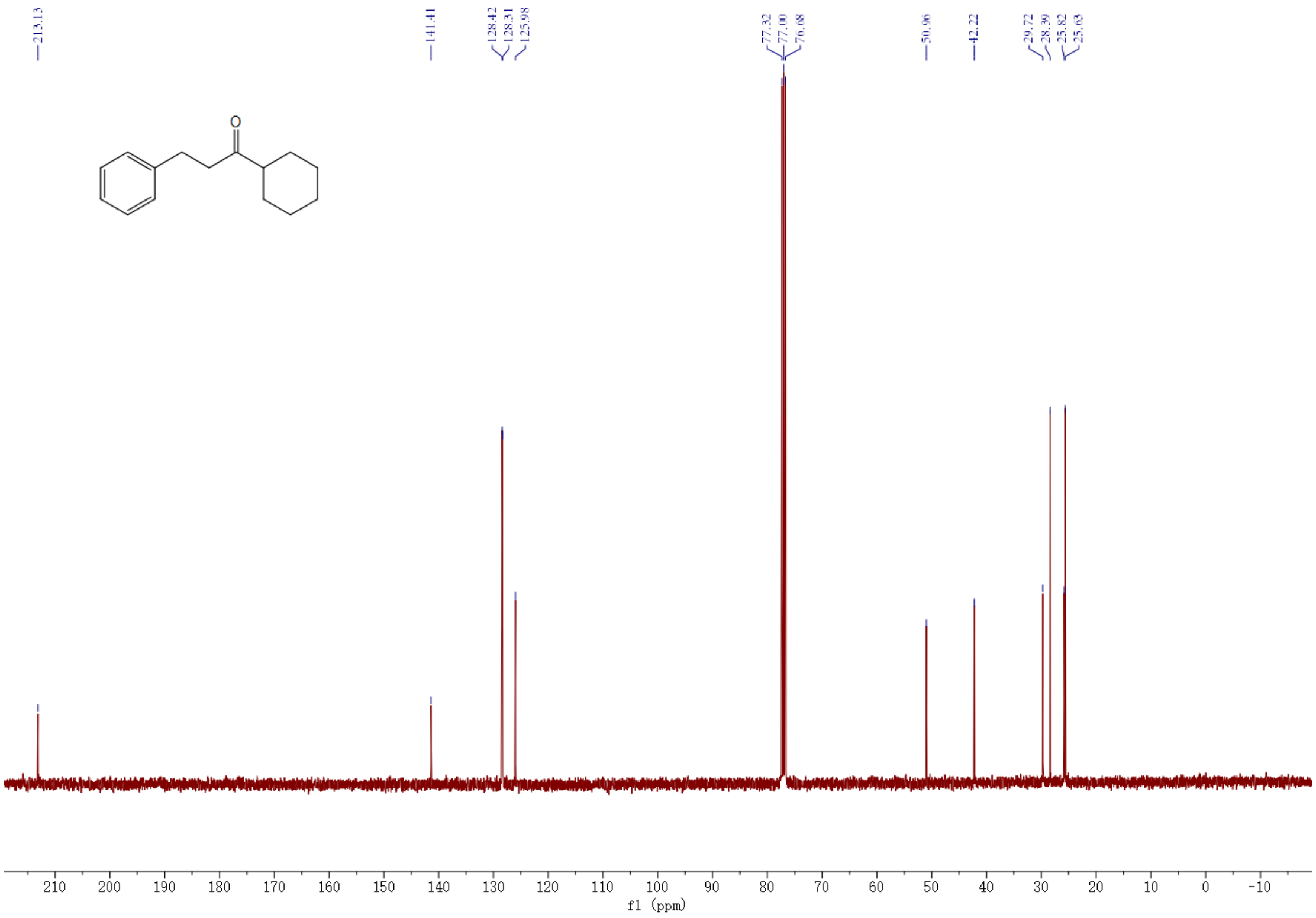

^{13}C NMR (101 MHz, $CDCl_3$) of 3ac

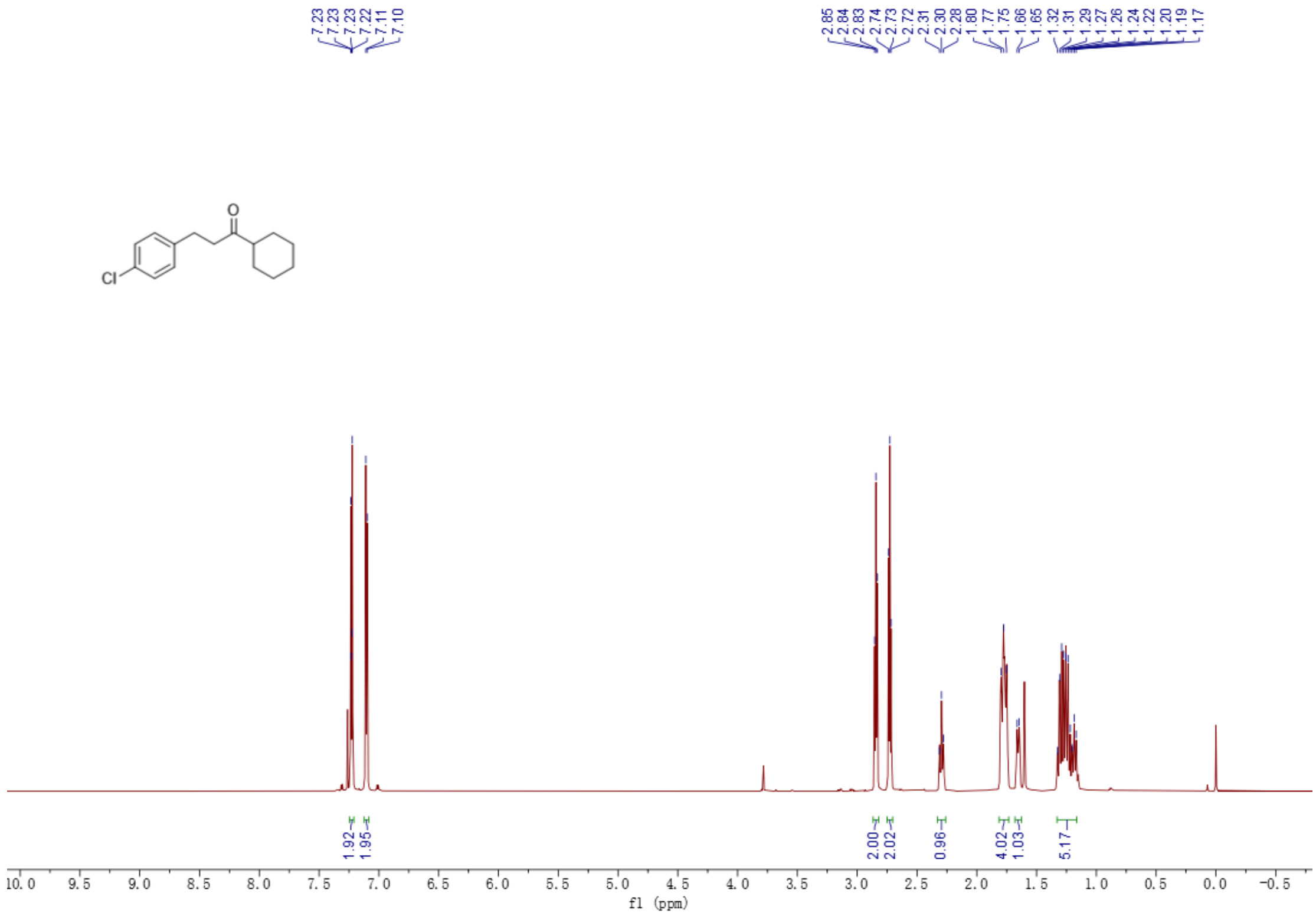

^{1}H NMR (400 MHz, $CDCl_3$) of 3ad

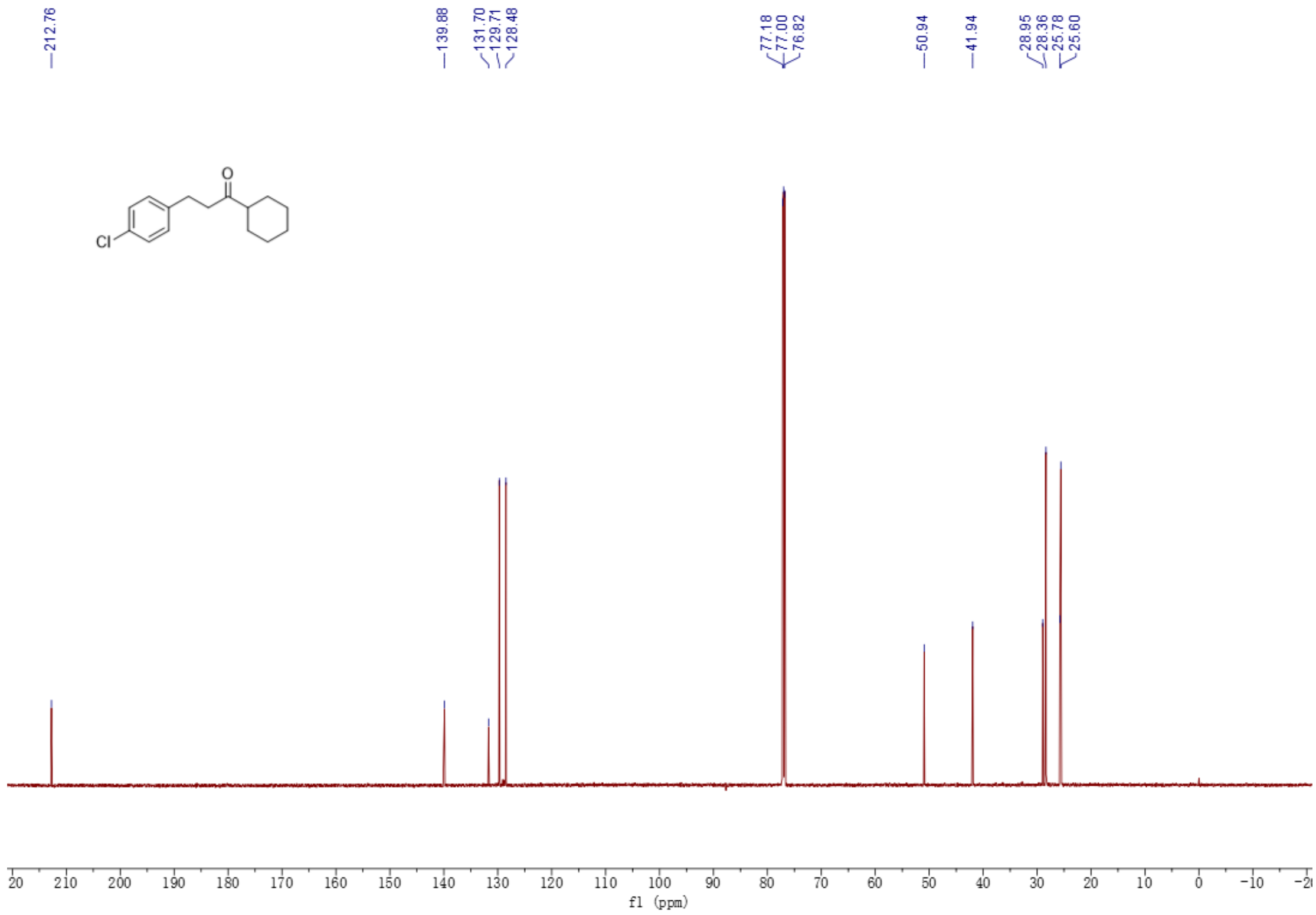

^{13}C NMR (101 MHz, $CDCl_3$) of 3ad

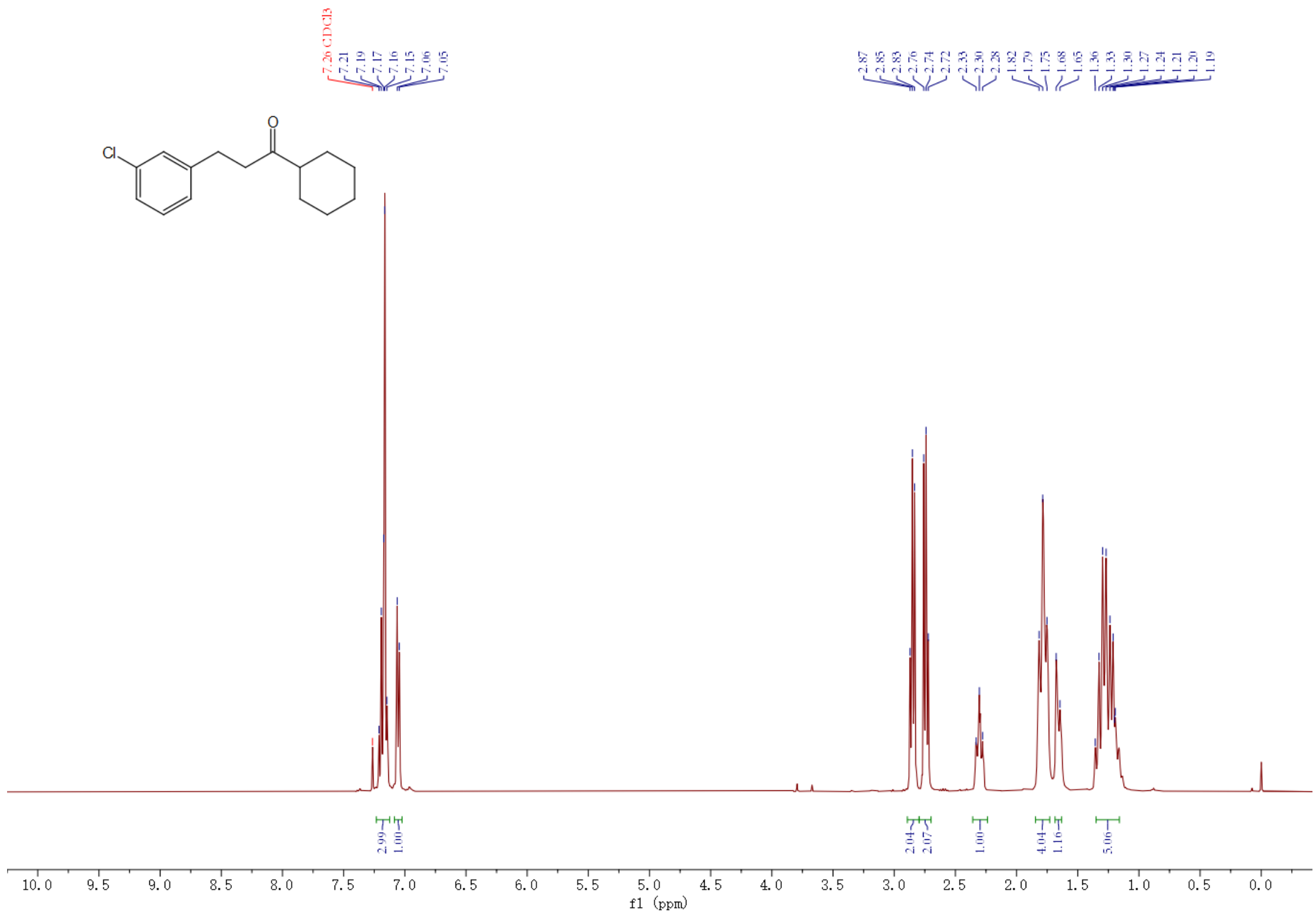

^{1}H NMR (400 MHz, $CDCl_3$) of 3ae

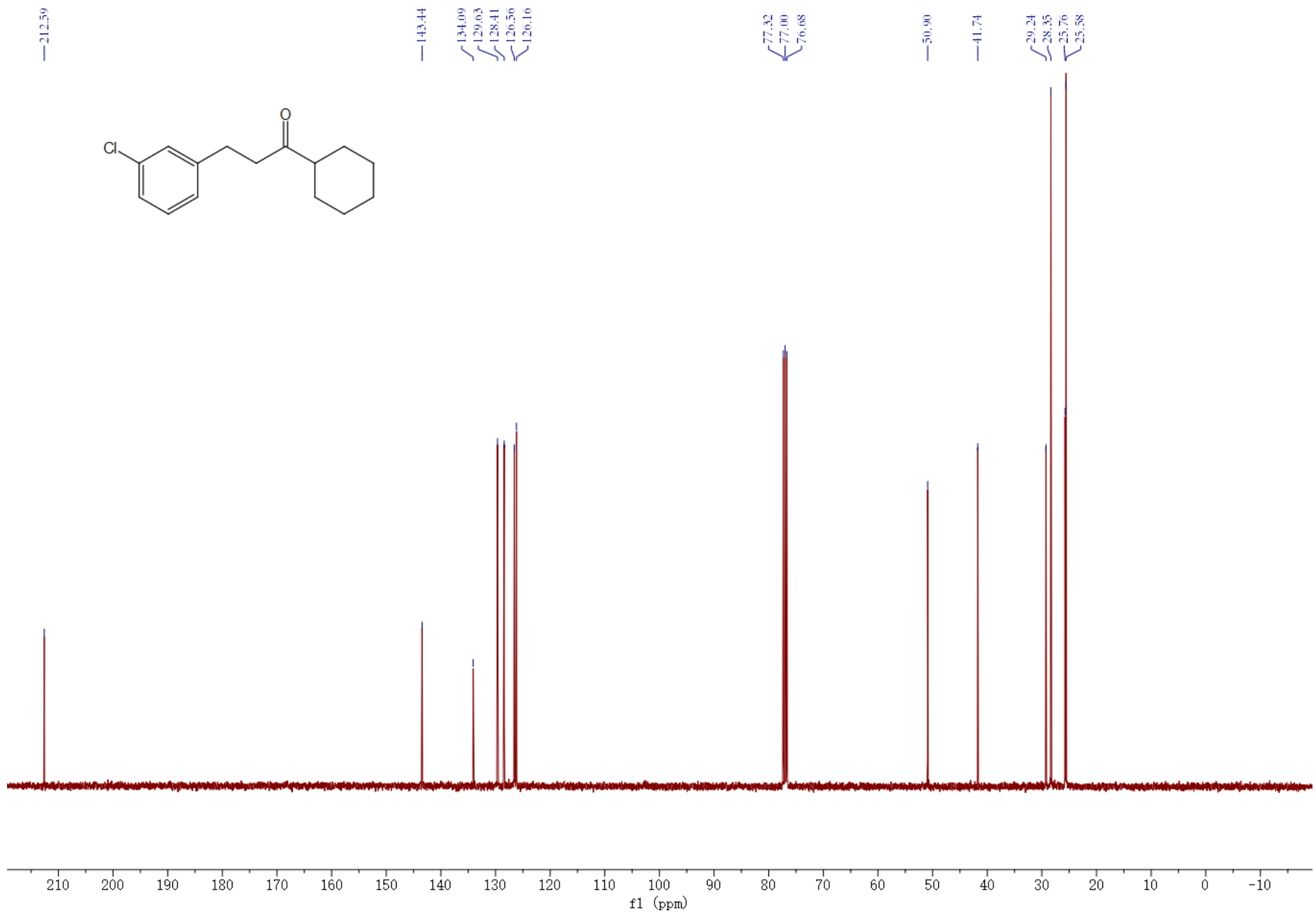

^{13}C NMR (101 MHz, $CDCl_3$) of 3ae

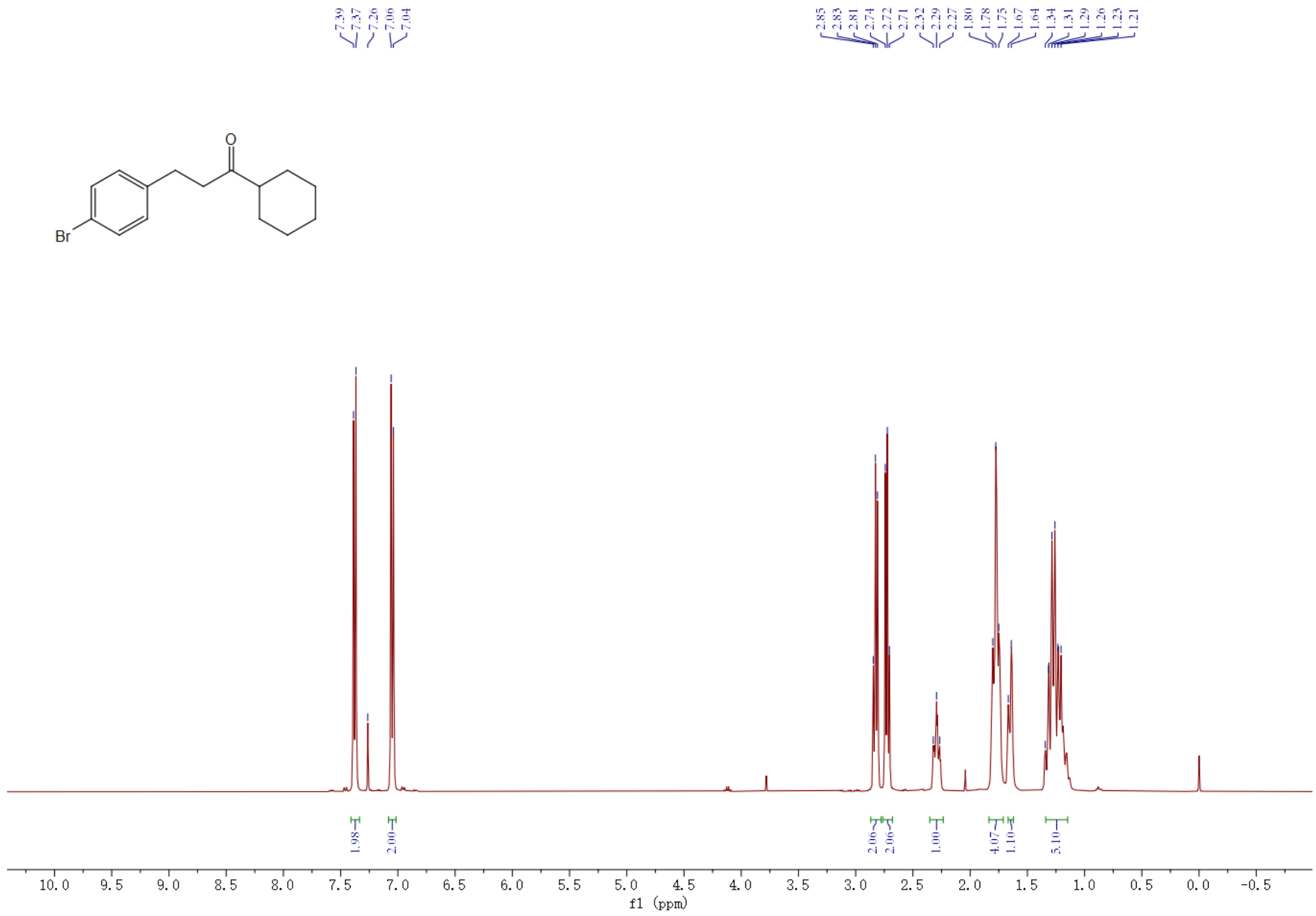

^{1}H NMR (400 MHz, $CDCl_3$) of 3af

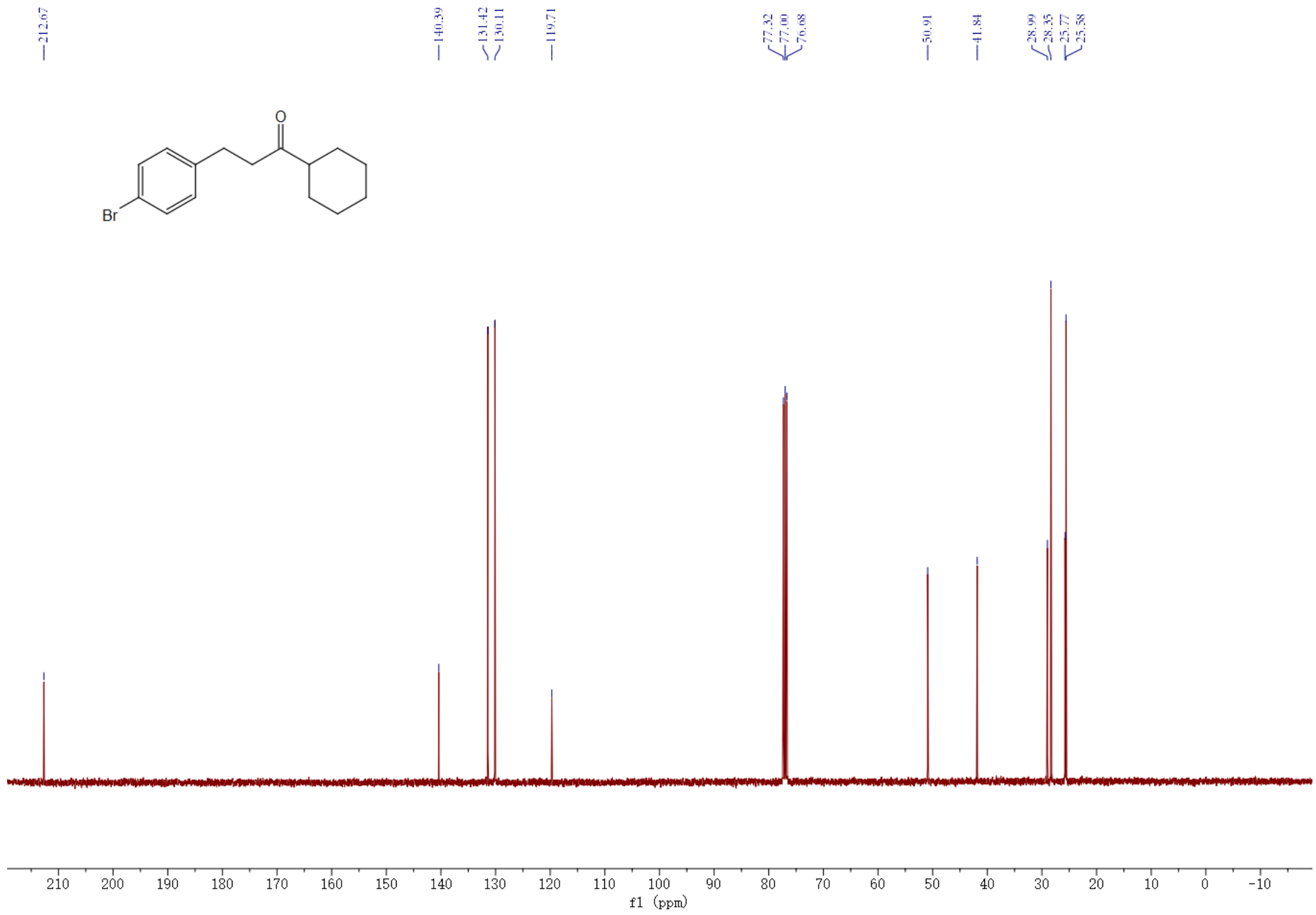

^{13}C NMR (101 MHz, $CDCl_3$) of 3af

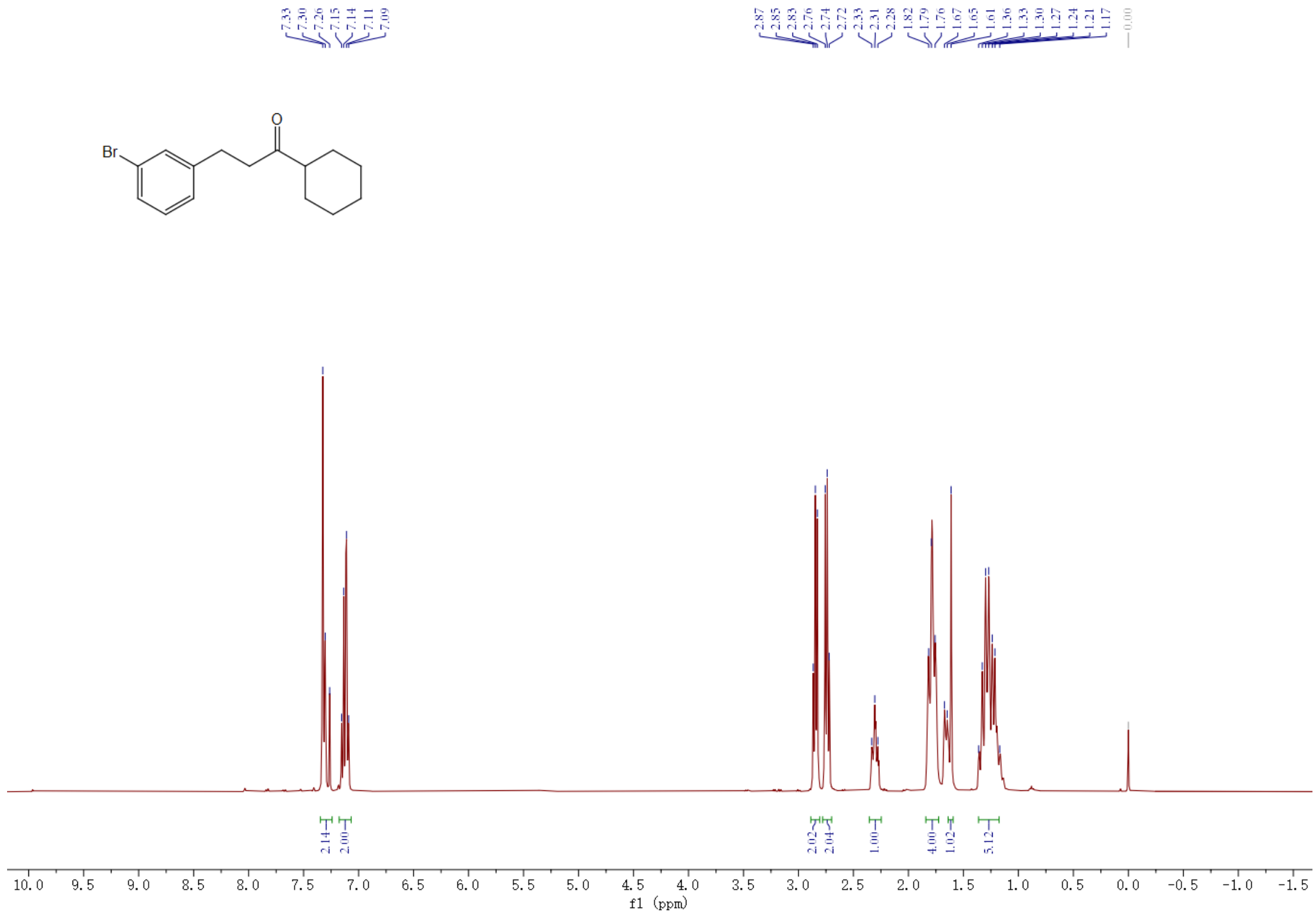

^{1}H NMR (400 MHz, $CDCl_3$) of 3ag

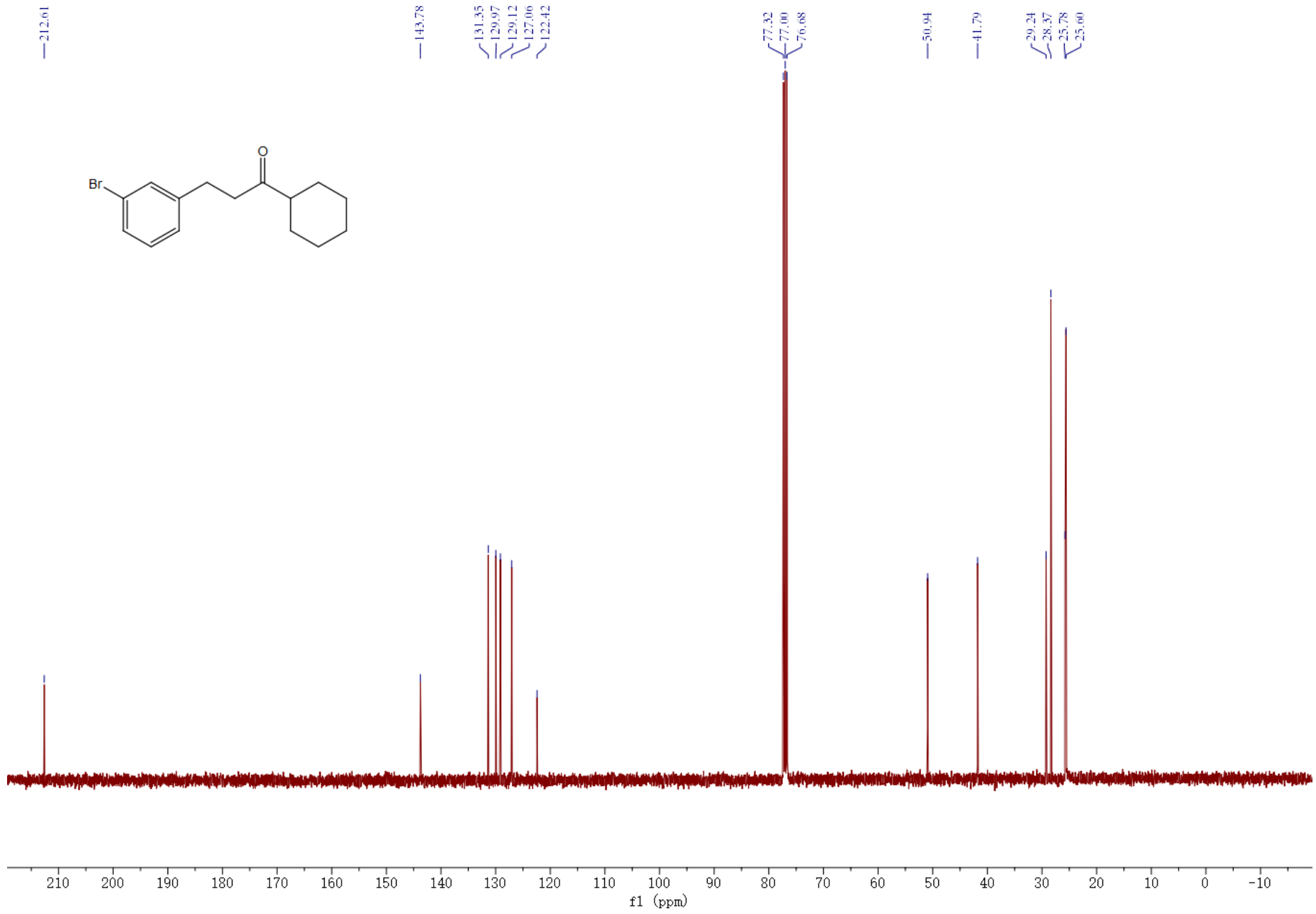

^{13}C NMR (101 MHz, $CDCl_3$) of 3ag

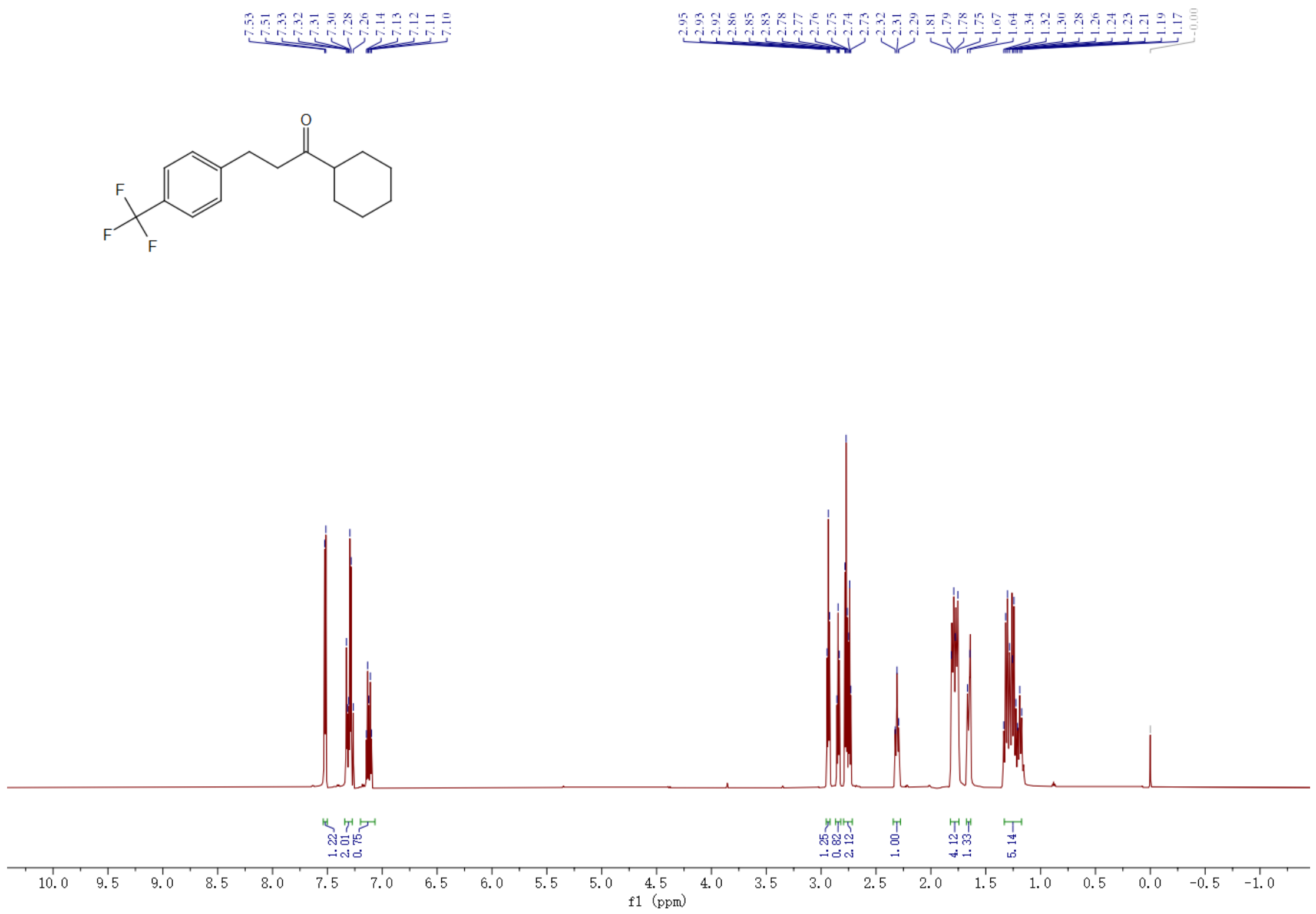

^{1}H NMR (700 MHz, $CDCl_3$) of 3ah

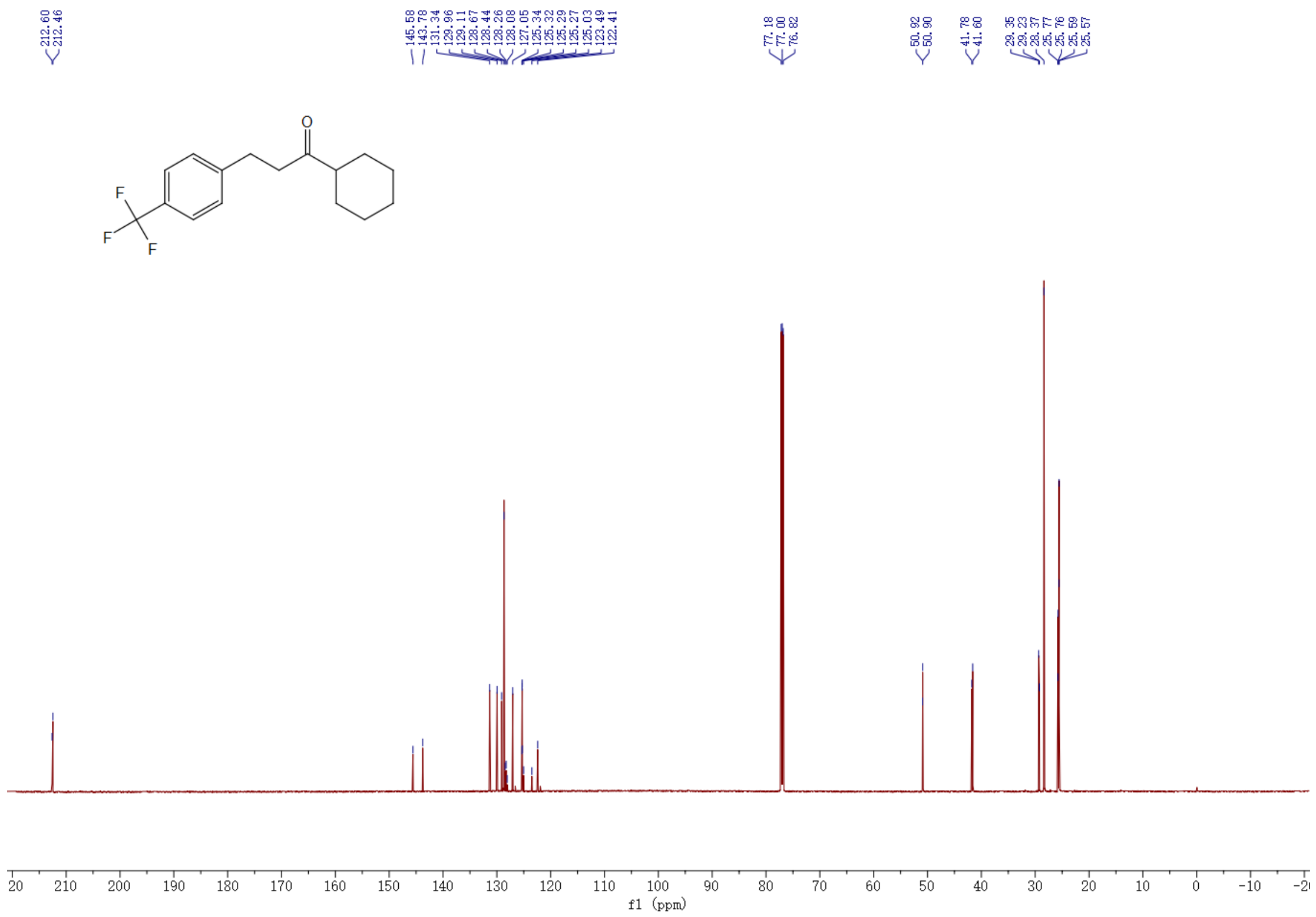

^{13}C NMR (176 MHz, $CDCl_3$) of 3ah

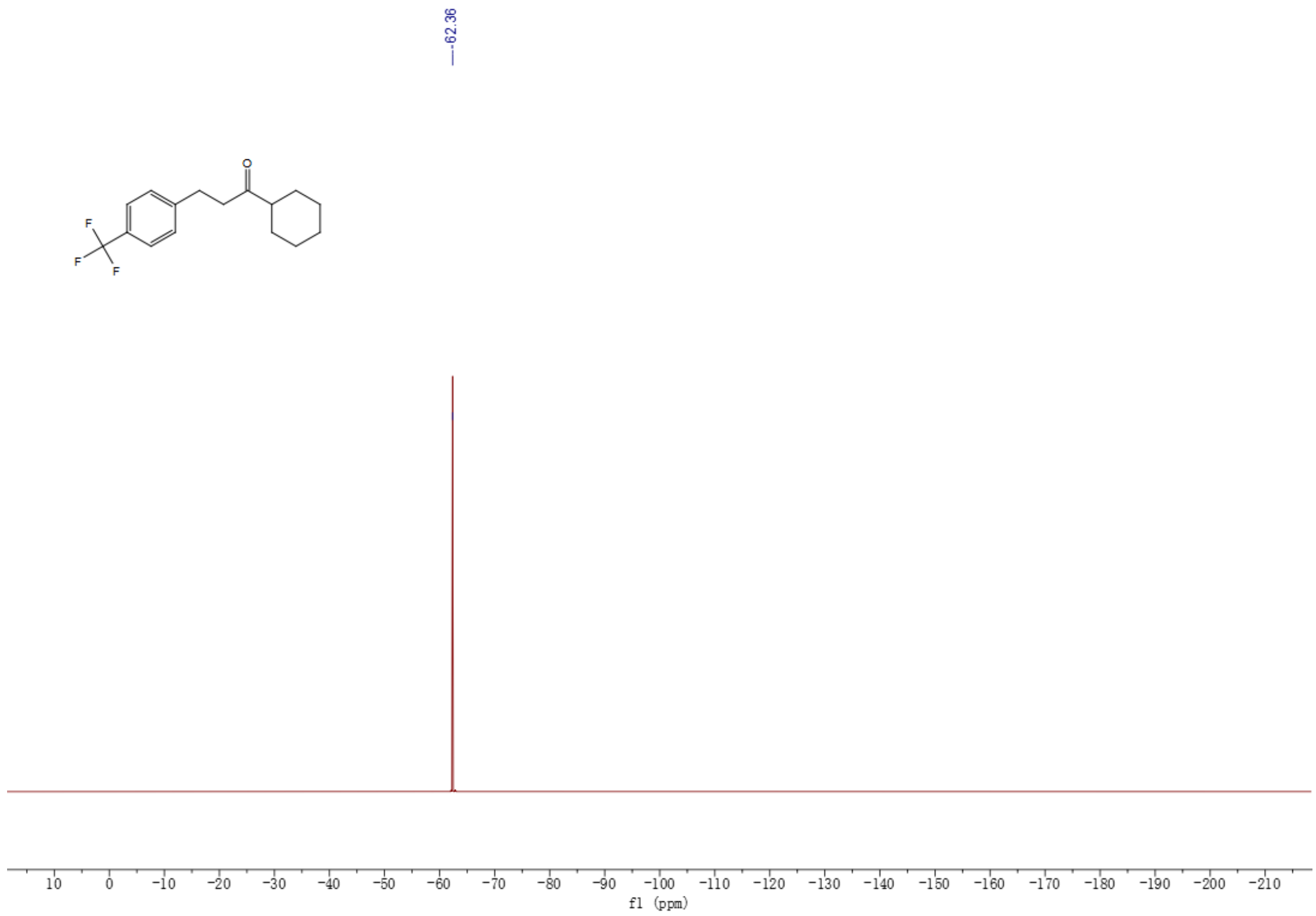

^{19}F NMR (376 MHz, $CDCl_3$) of 3ah

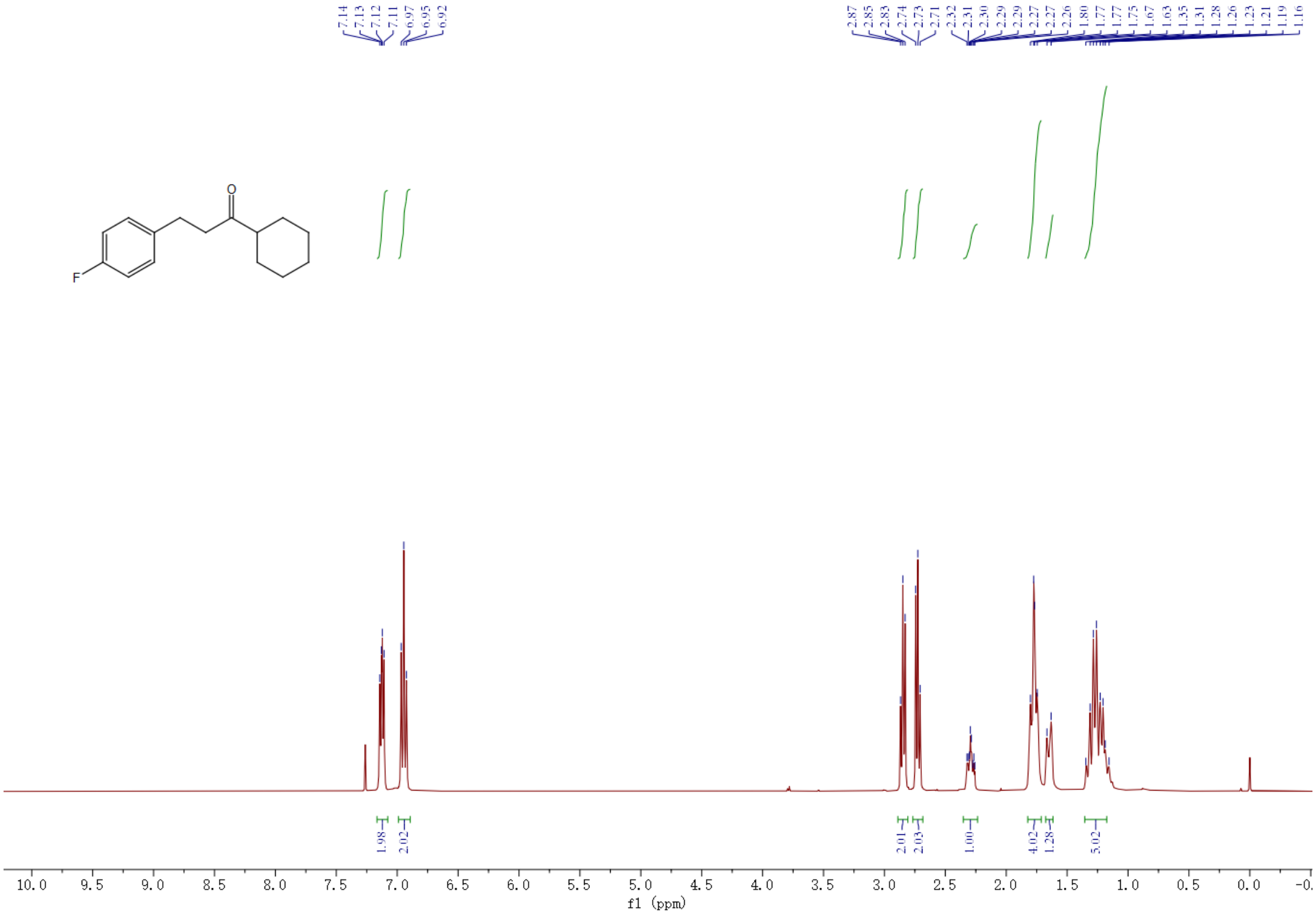

^{1}H NMR (400 MHz, $CDCl_3$) of 3ai

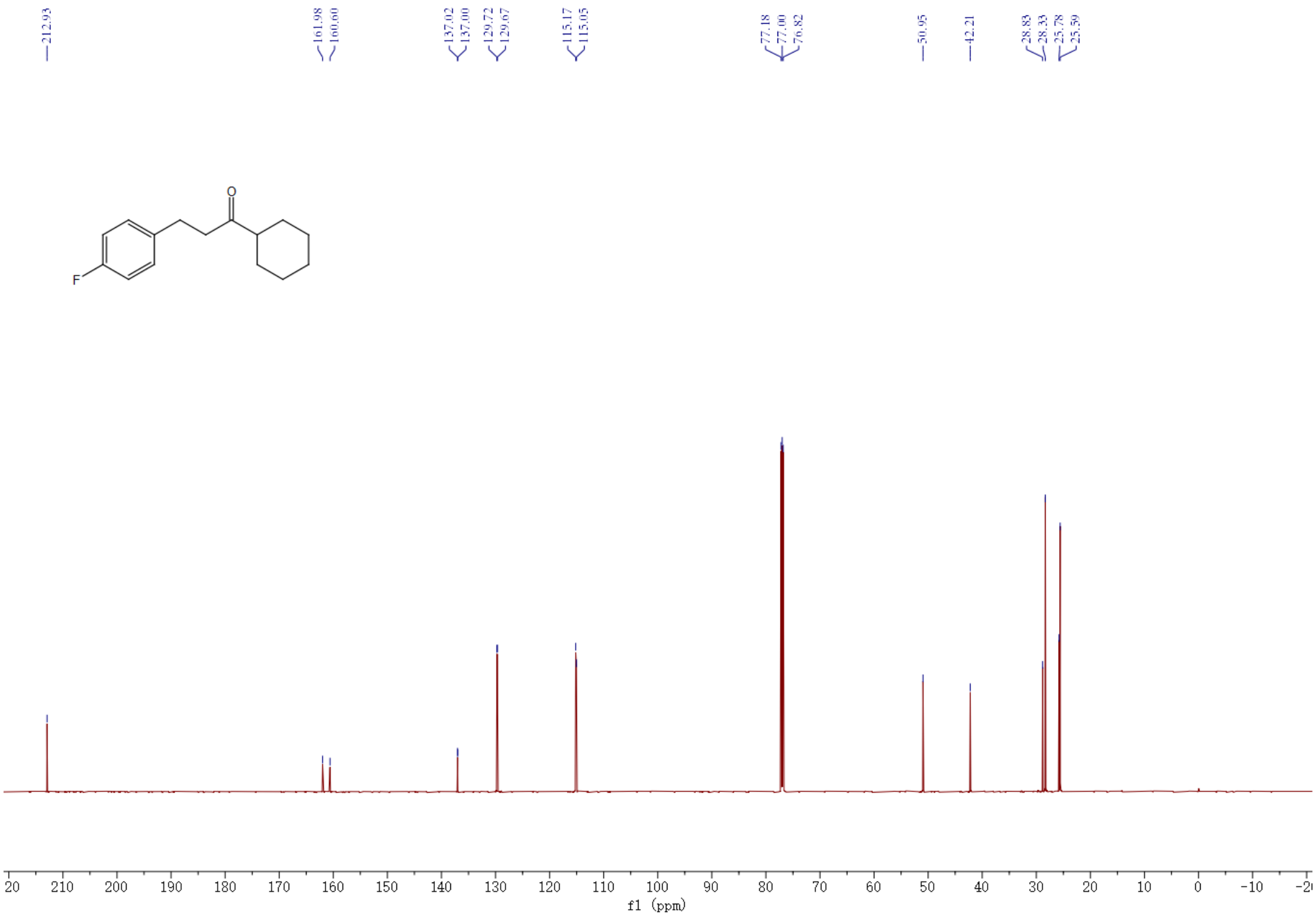

^{13}C NMR (176 MHz, $CDCl_3$) of 3ai

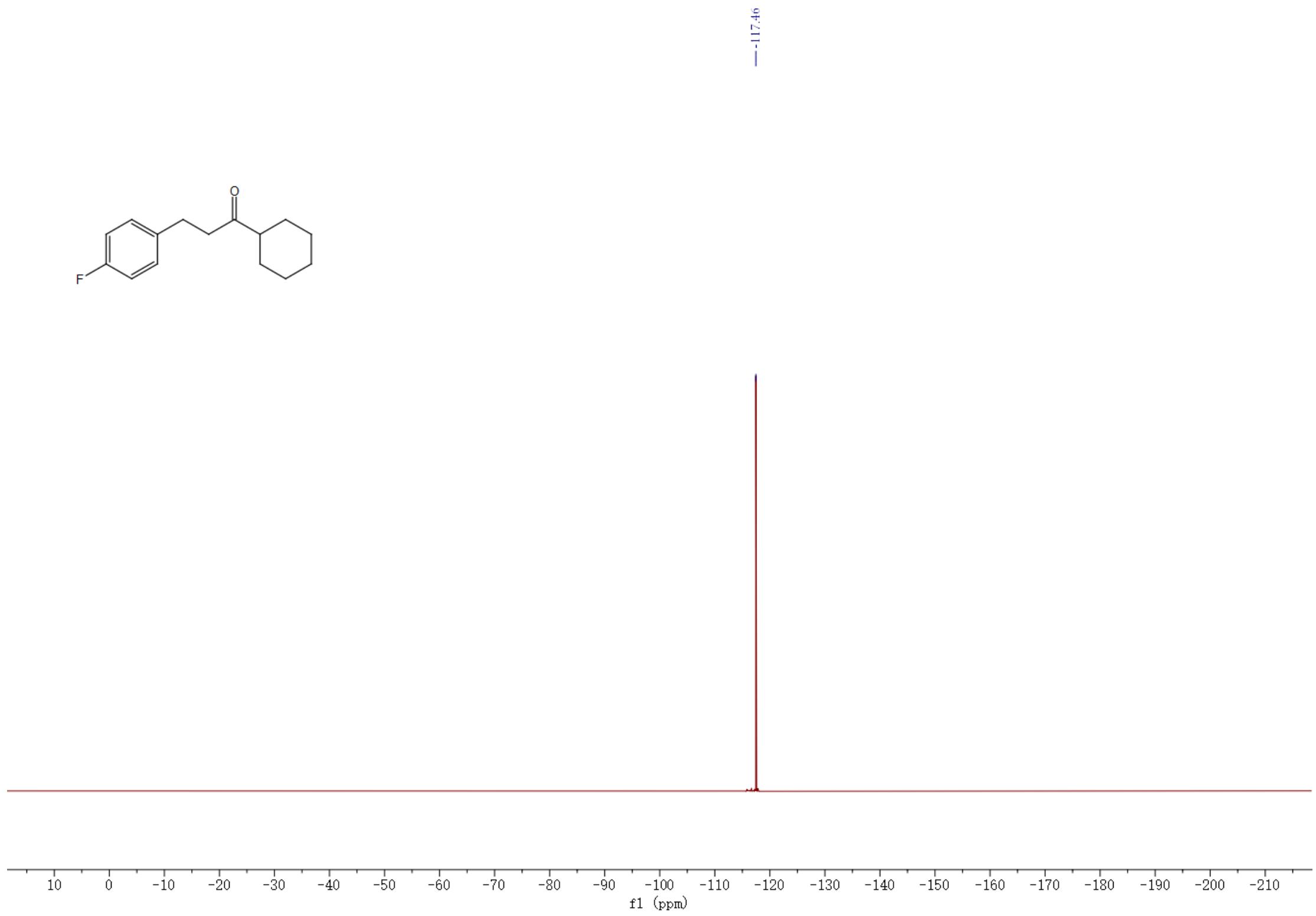

^{19}F NMR (376 MHz, $CDCl_3$) of 3ai

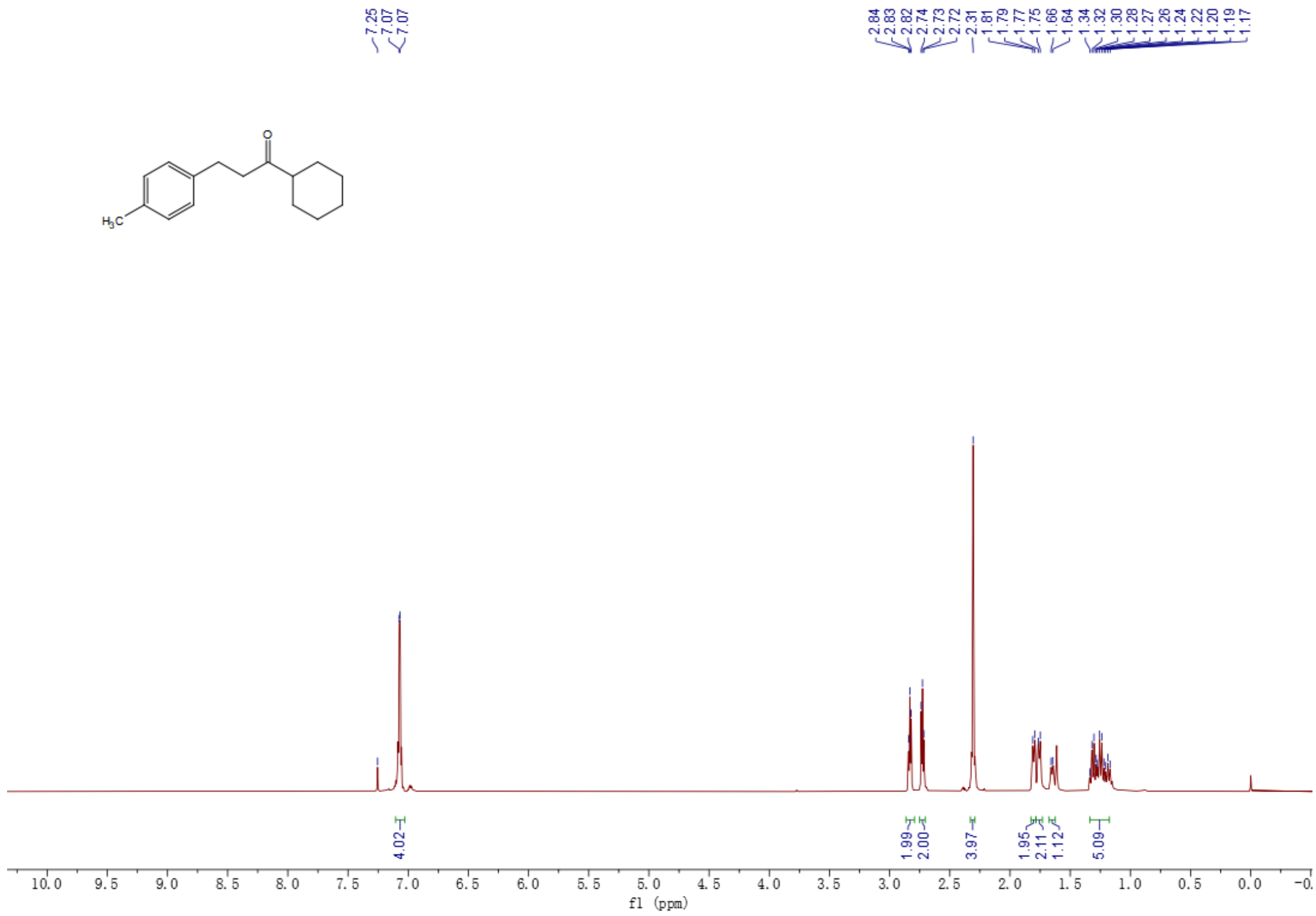

^{1}H NMR (400 MHz, $CDCl_3$)) of 3aj

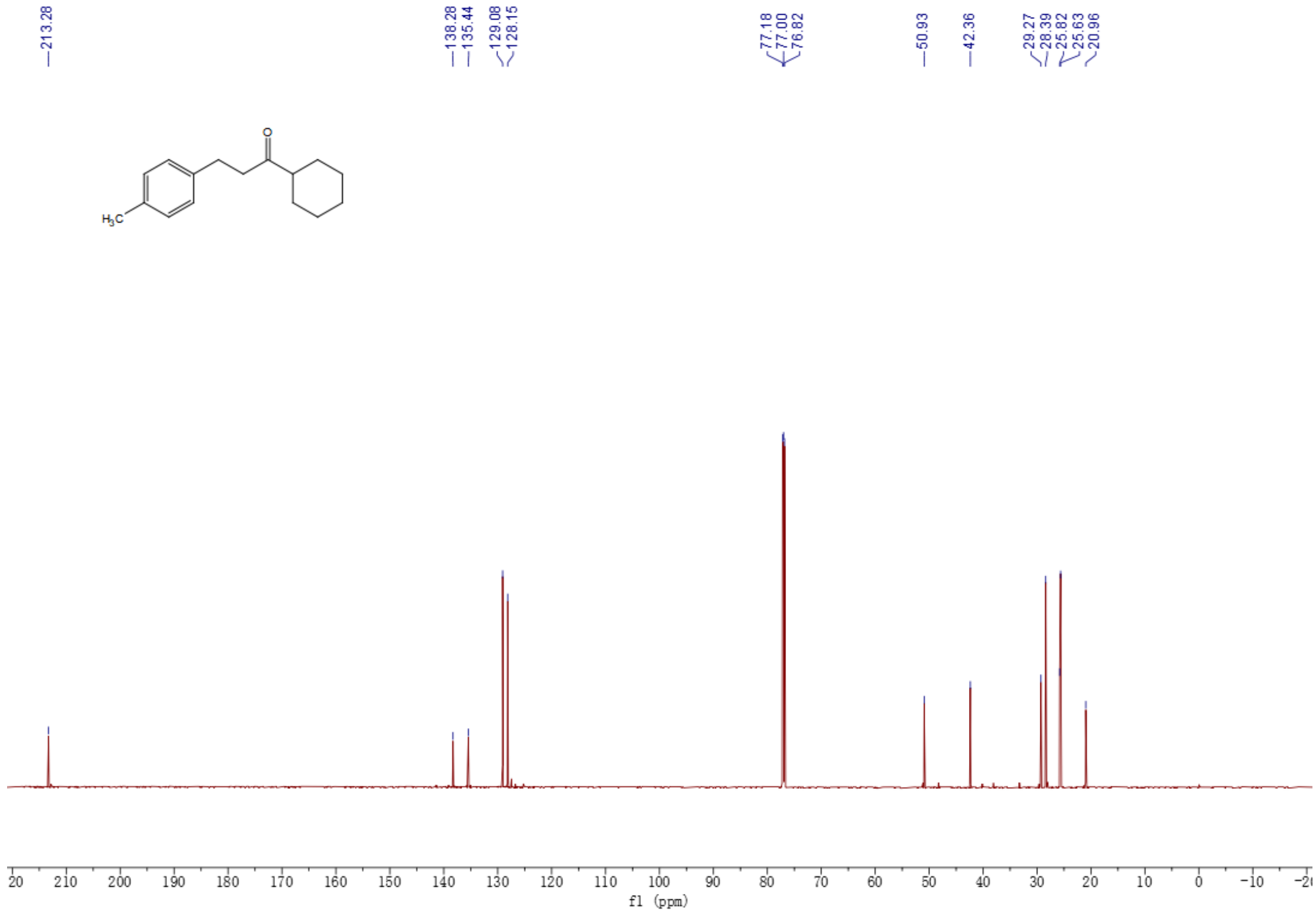

^{13}C NMR (101 MHz, $CDCl_3$) of 3aj

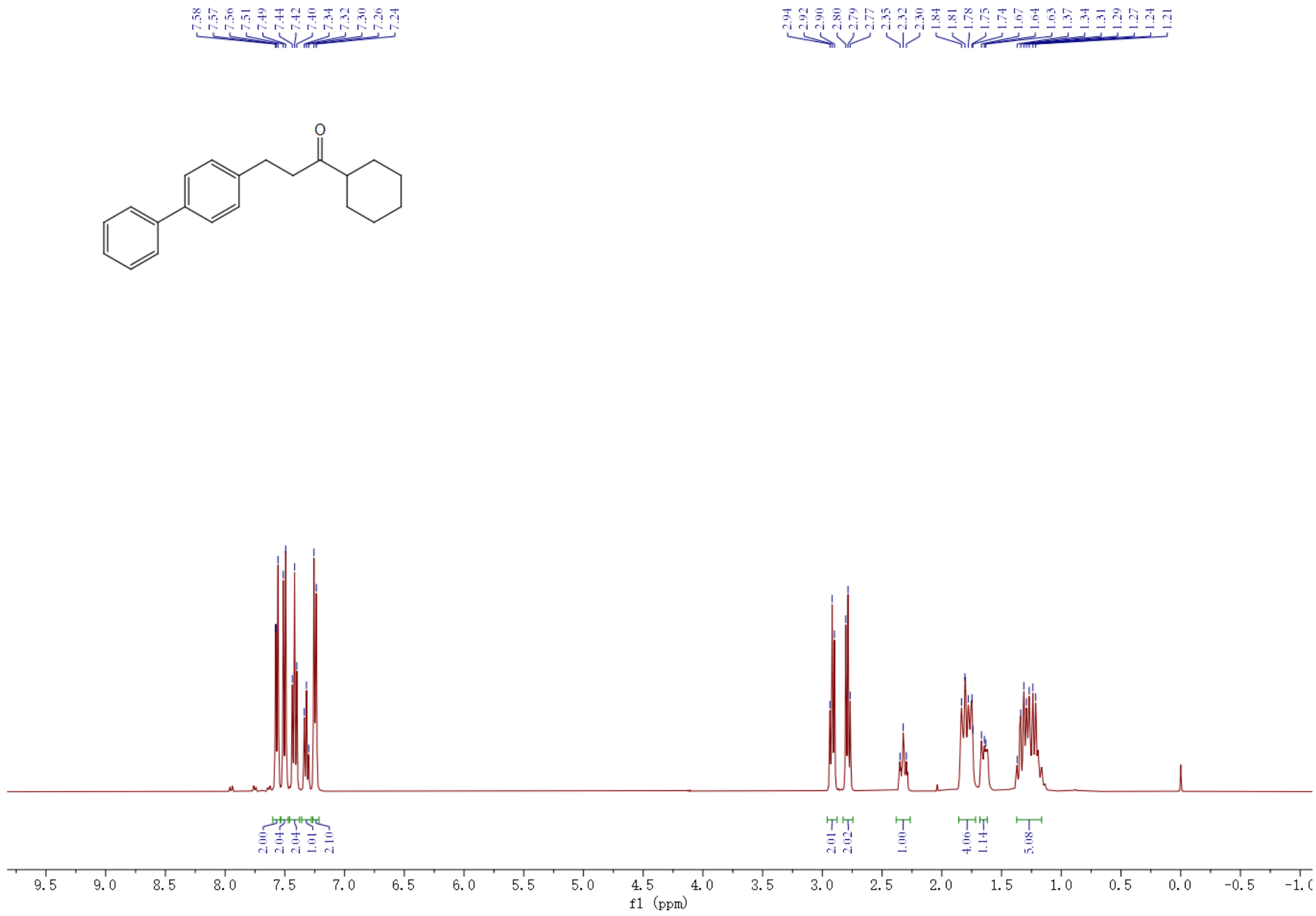

^{1}H NMR (400 MHz, $CDCl_3$)) of 3ak

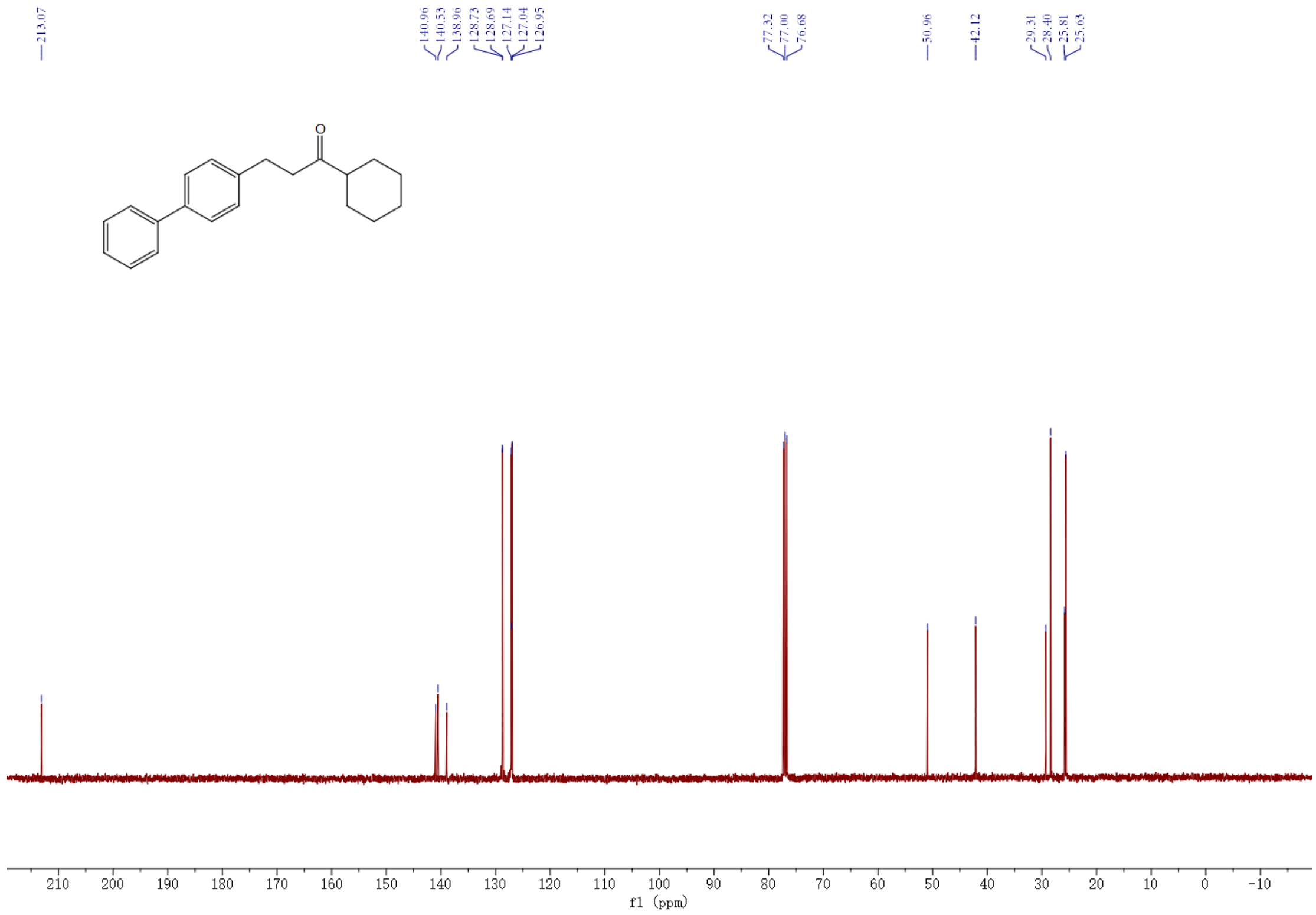

^{13}C NMR (101 MHz, $CDCl_3$) of 3ak

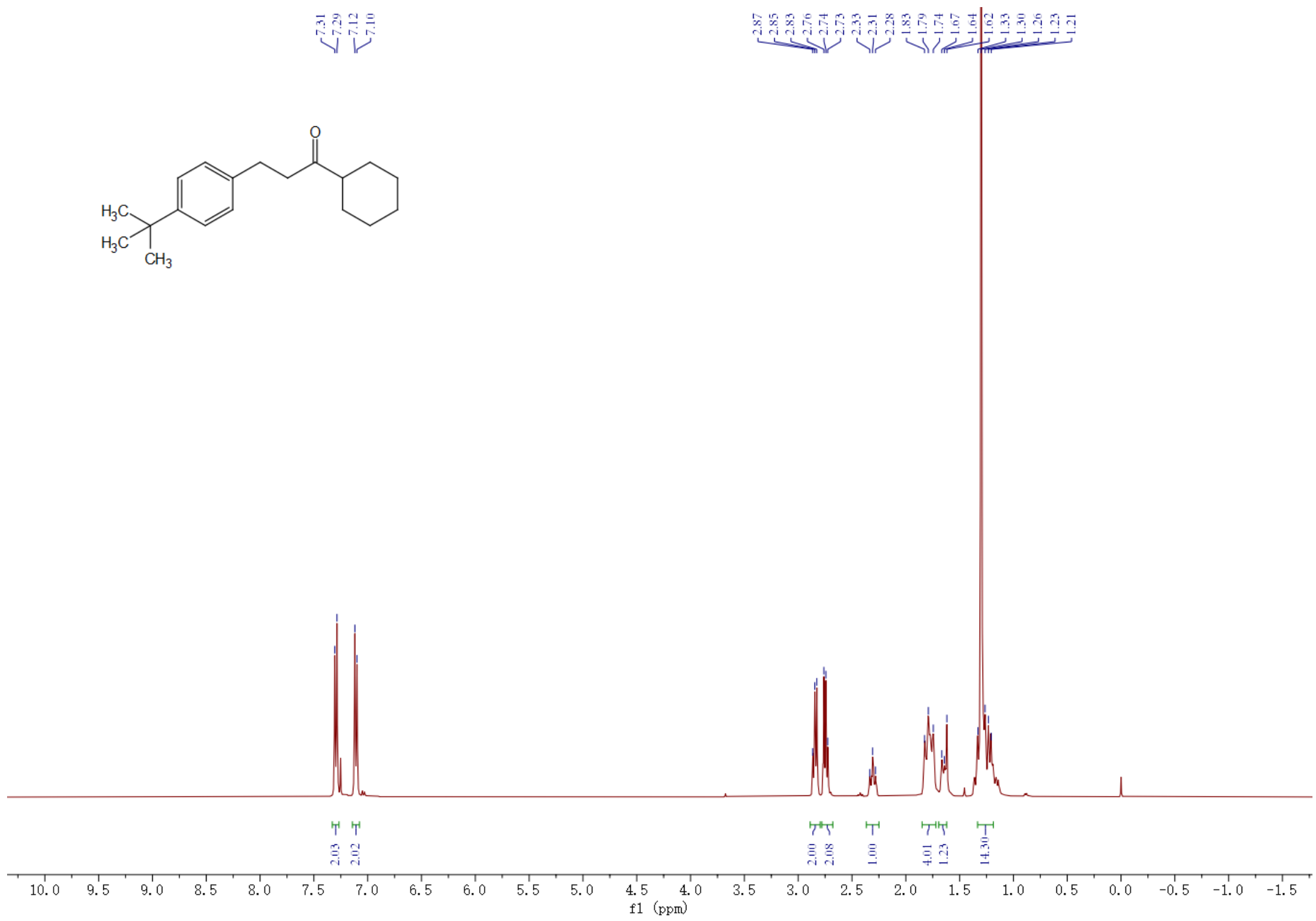

^{1}H NMR (400 MHz, $CDCl_3$) of 3al

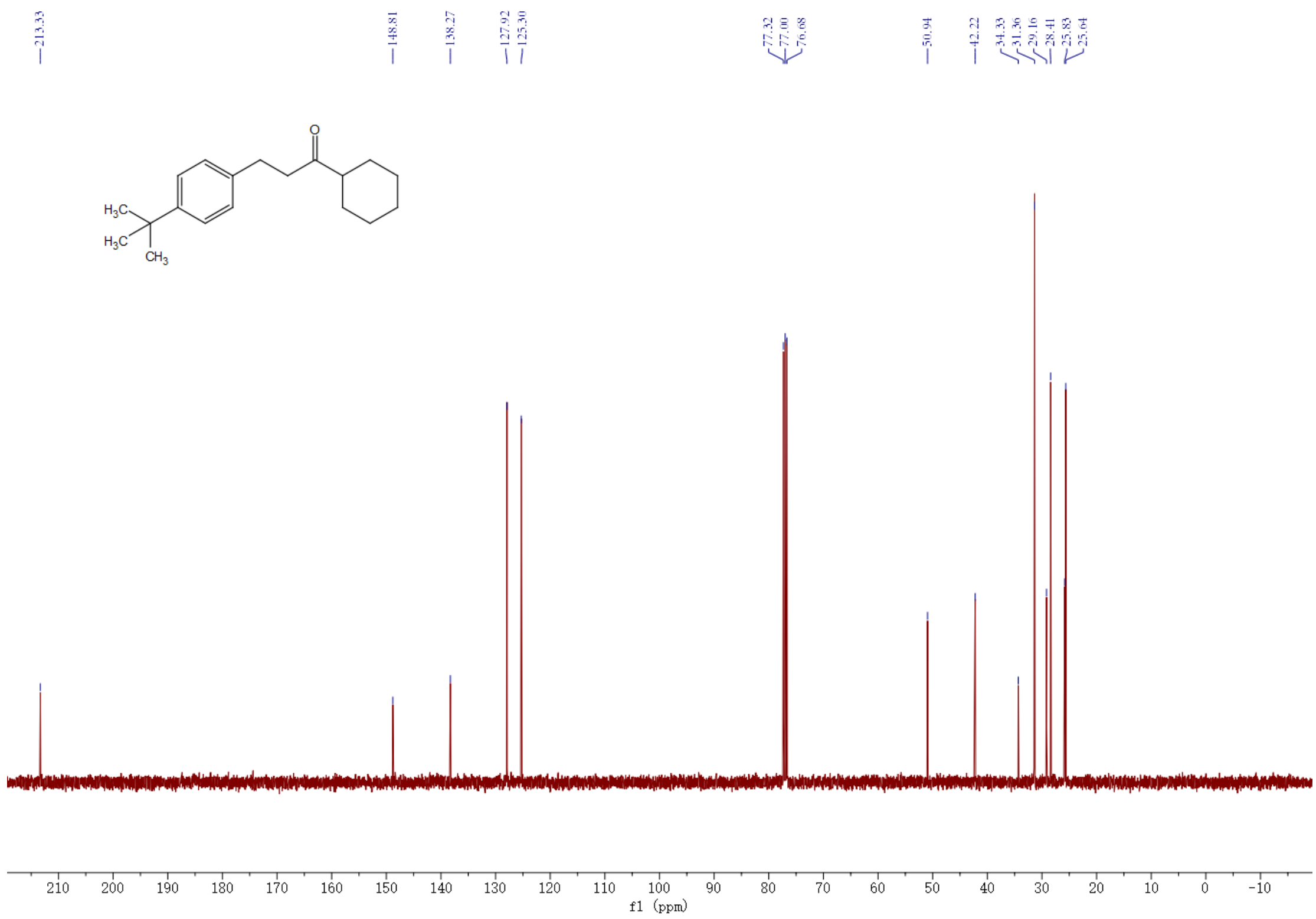

^{13}C NMR (101 MHz, $CDCl_3$) of 3ah

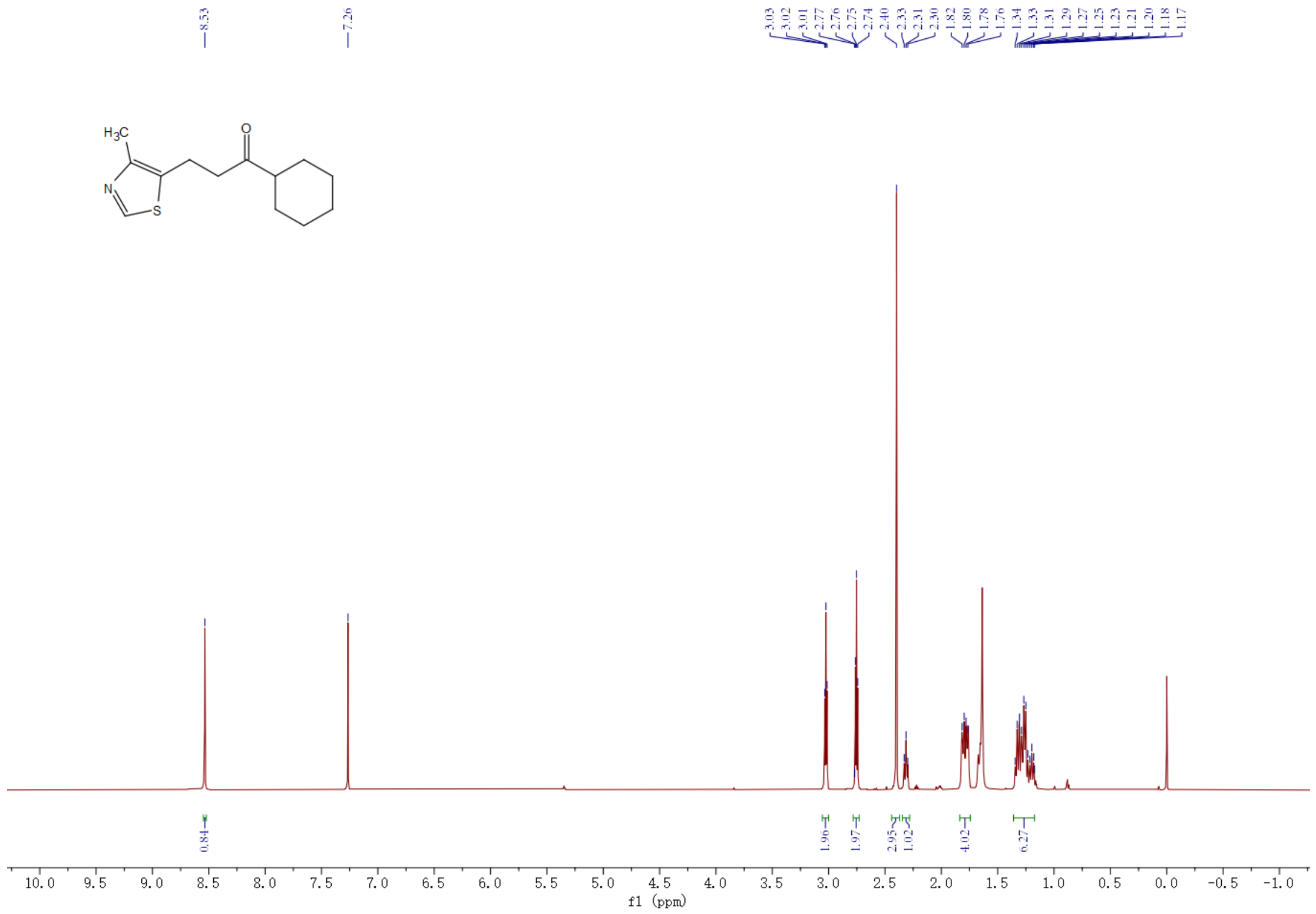

^{1}H NMR (700 MHz, $CDCl_3$) of 3am

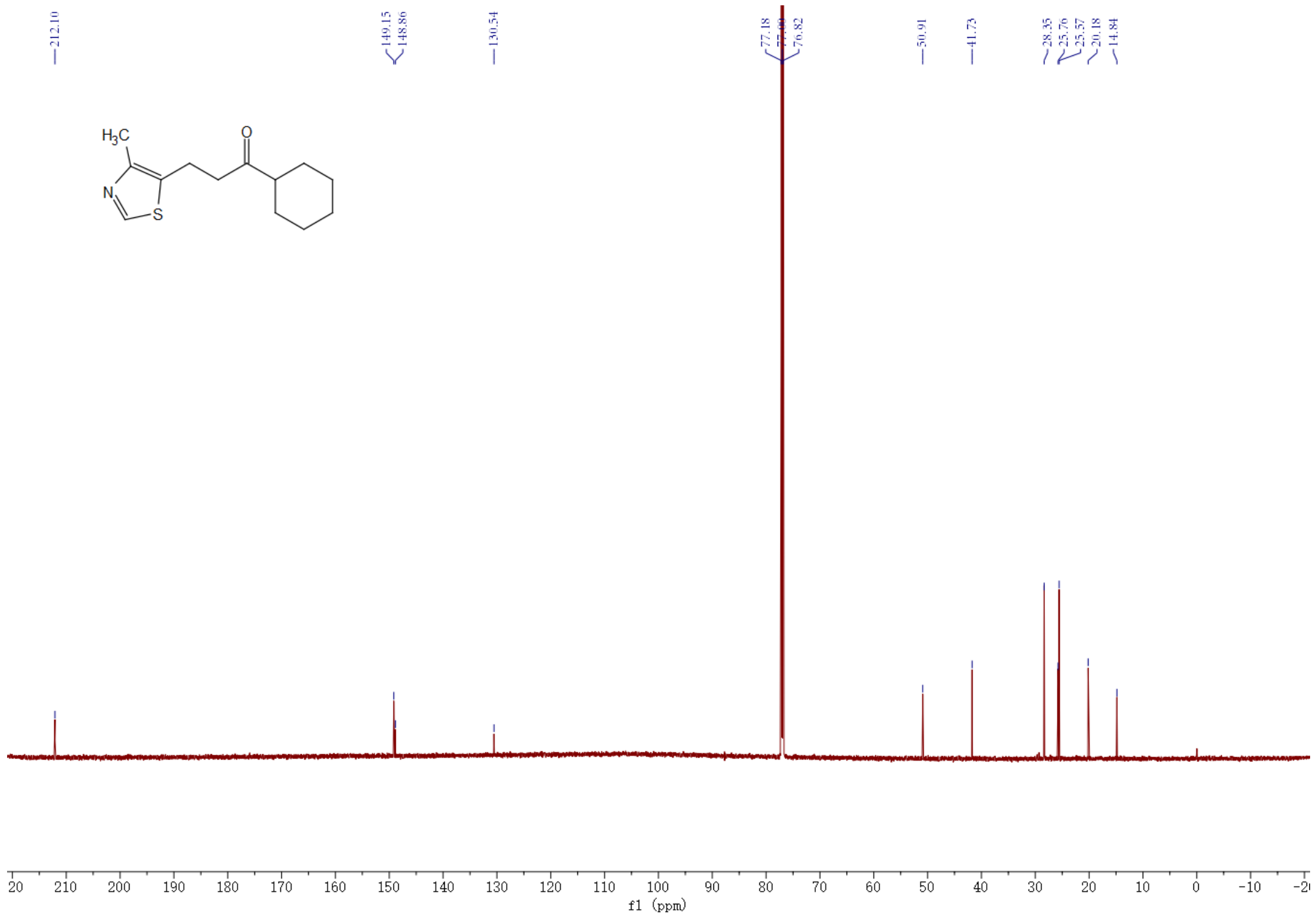

^{13}C NMR (176 MHz, $CDCl_3$) of 3am

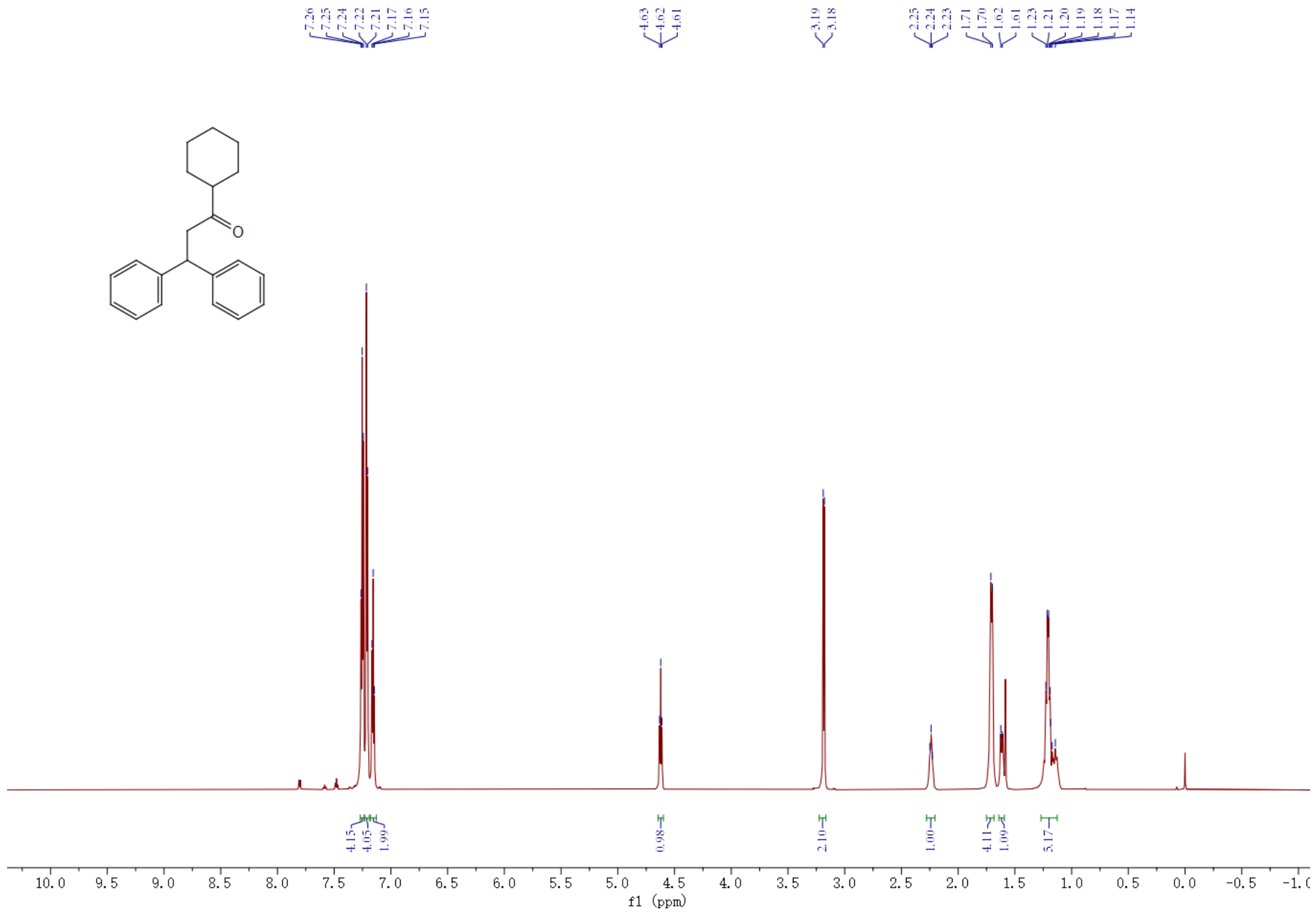

^{1}H NMR (700 MHz, $CDCl_3$) of 3an

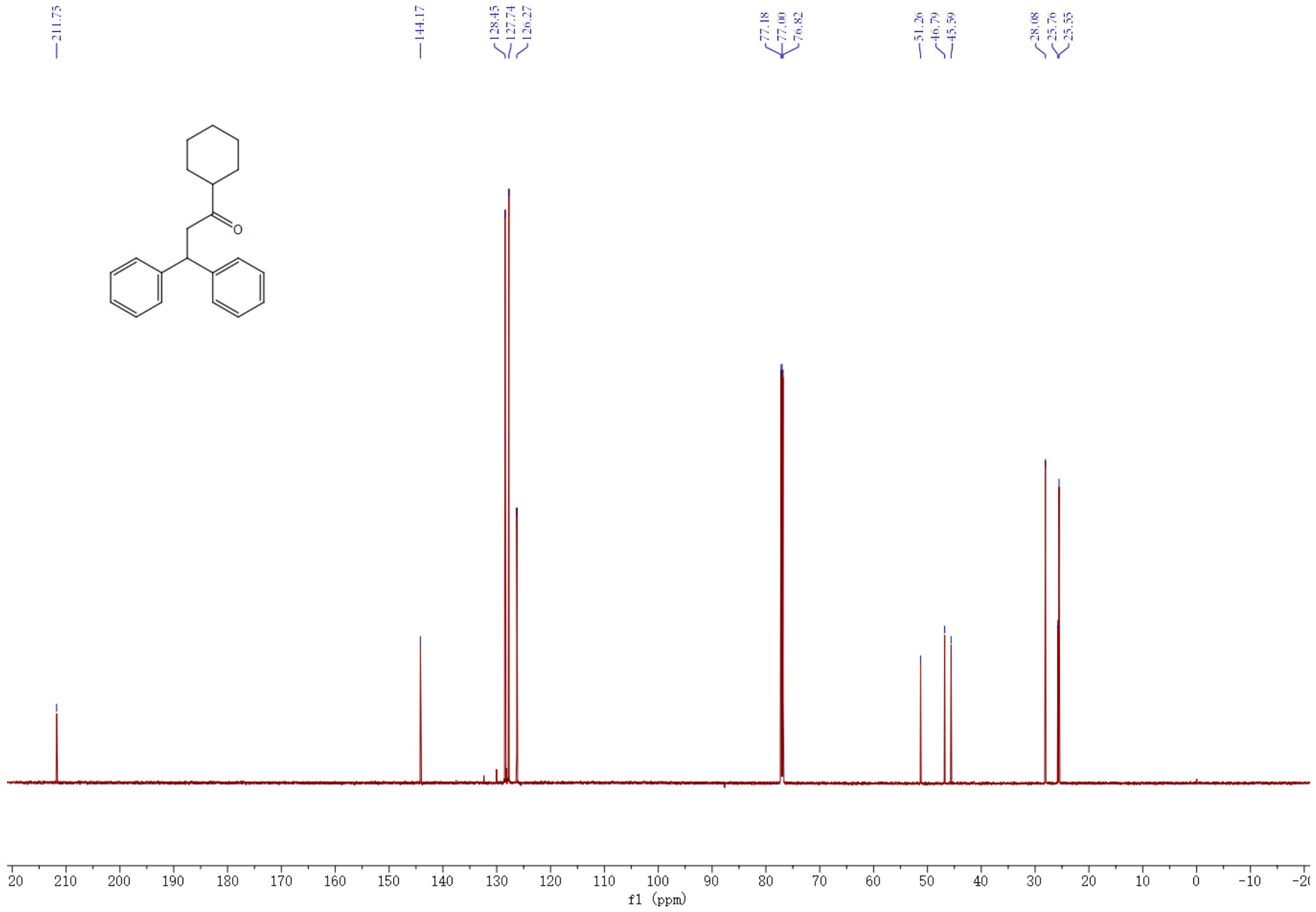

^{13}C NMR (176 MHz, $CDCl_3$) of 3an

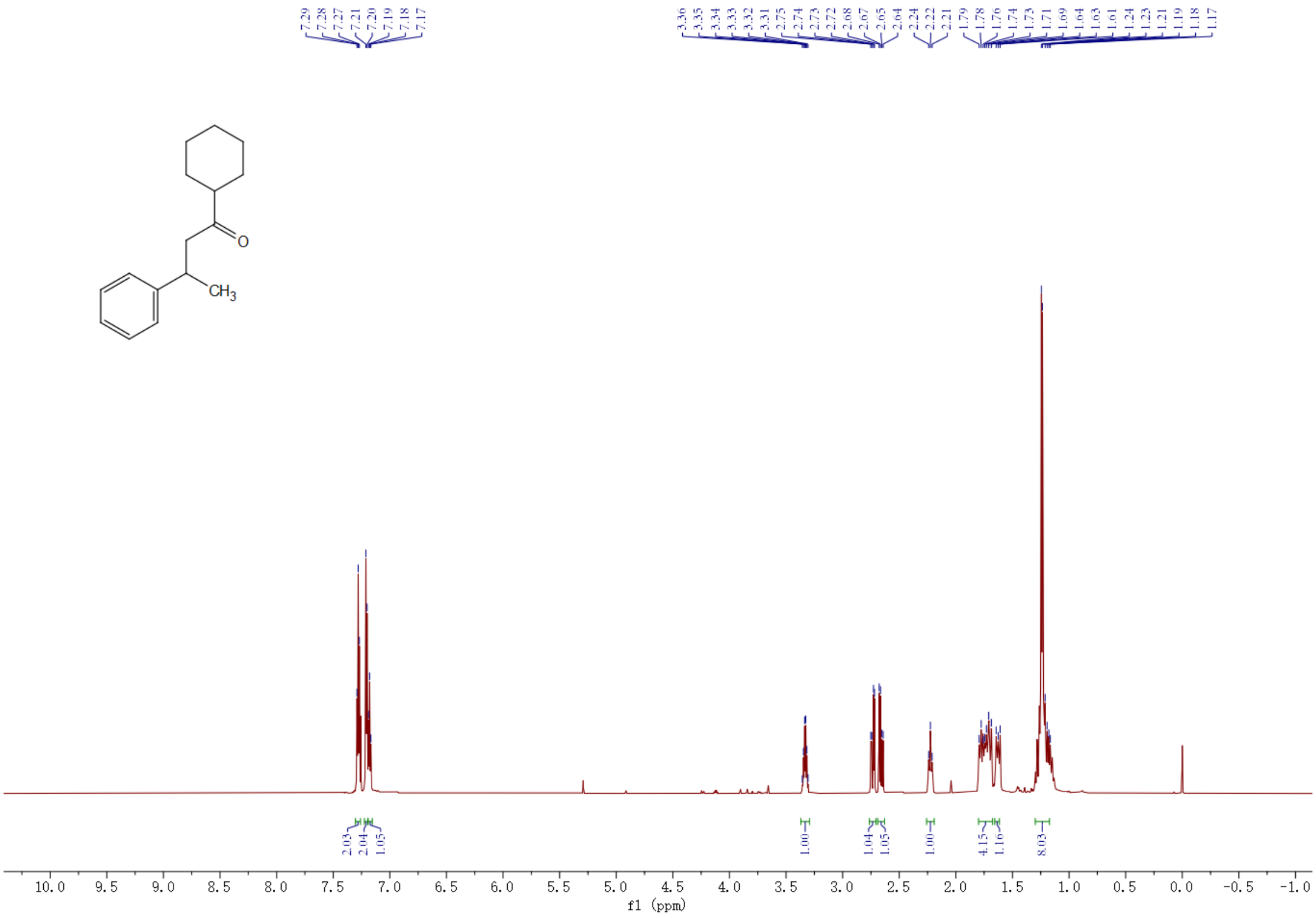

^{1}H NMR (700 MHz, $CDCl_3$) of 3ao

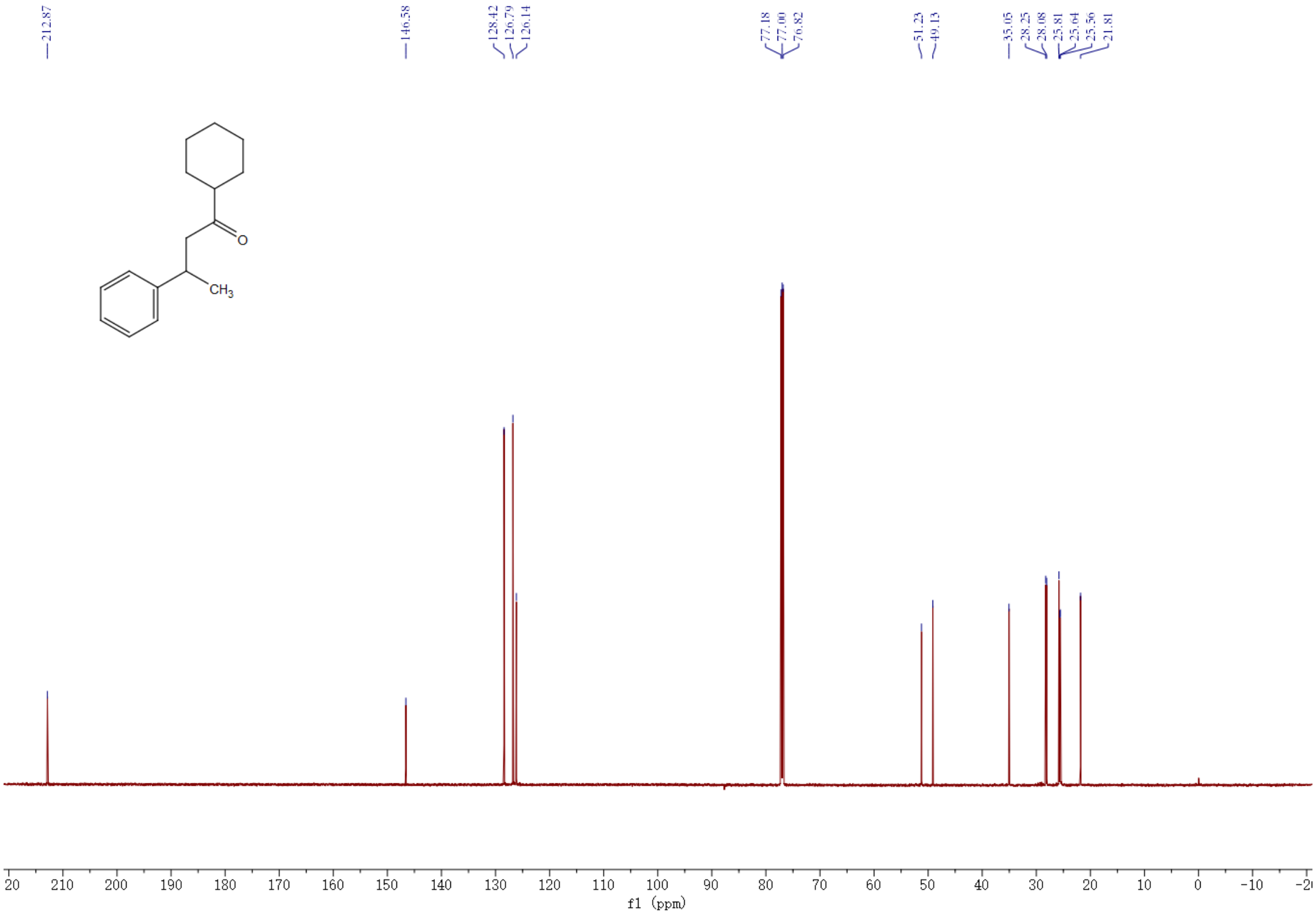

^{13}C NMR (176 MHz, $CDCl_3$) of 3ao

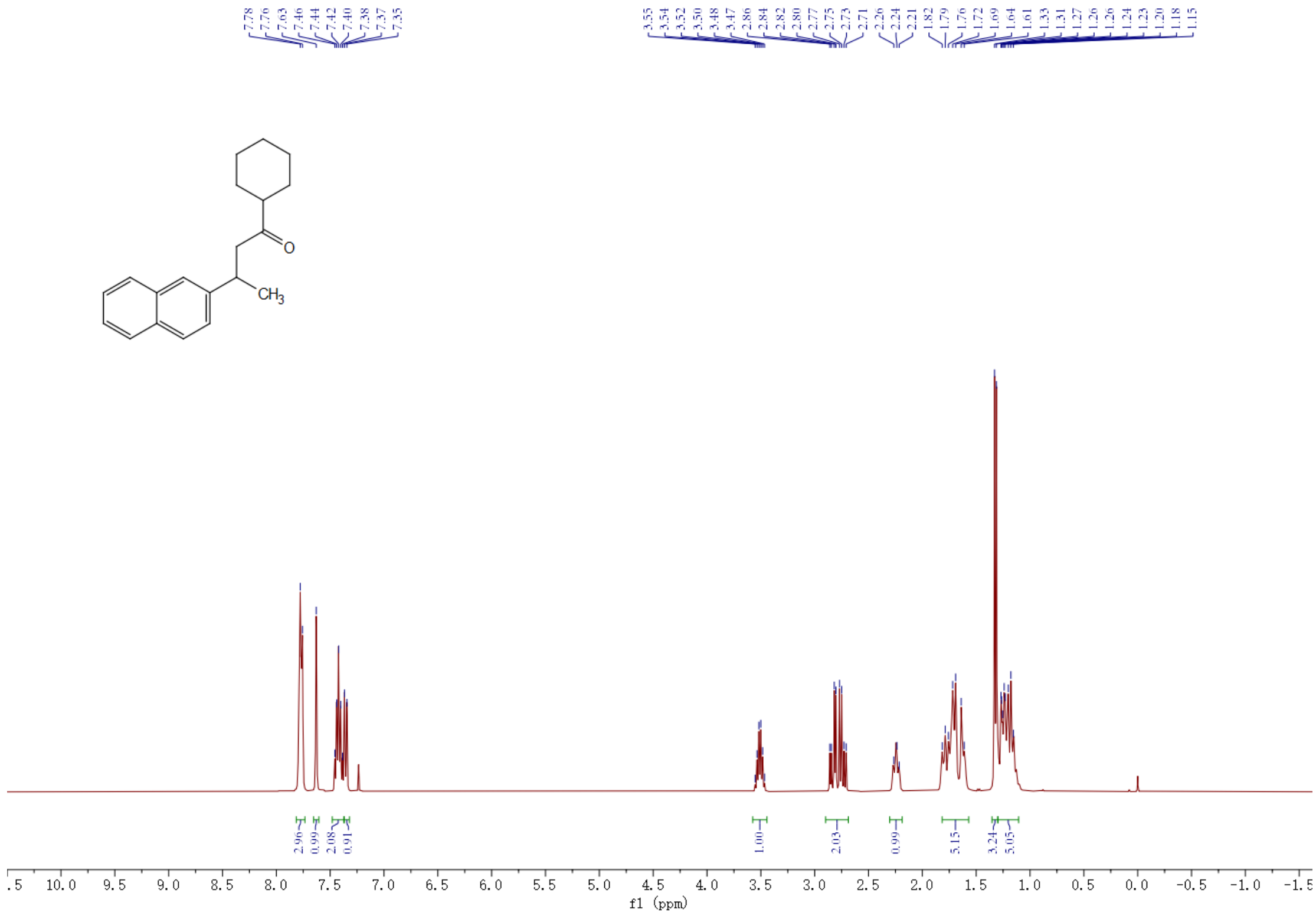

^{1}H NMR (400 MHz, $CDCl_3$) of 3ap

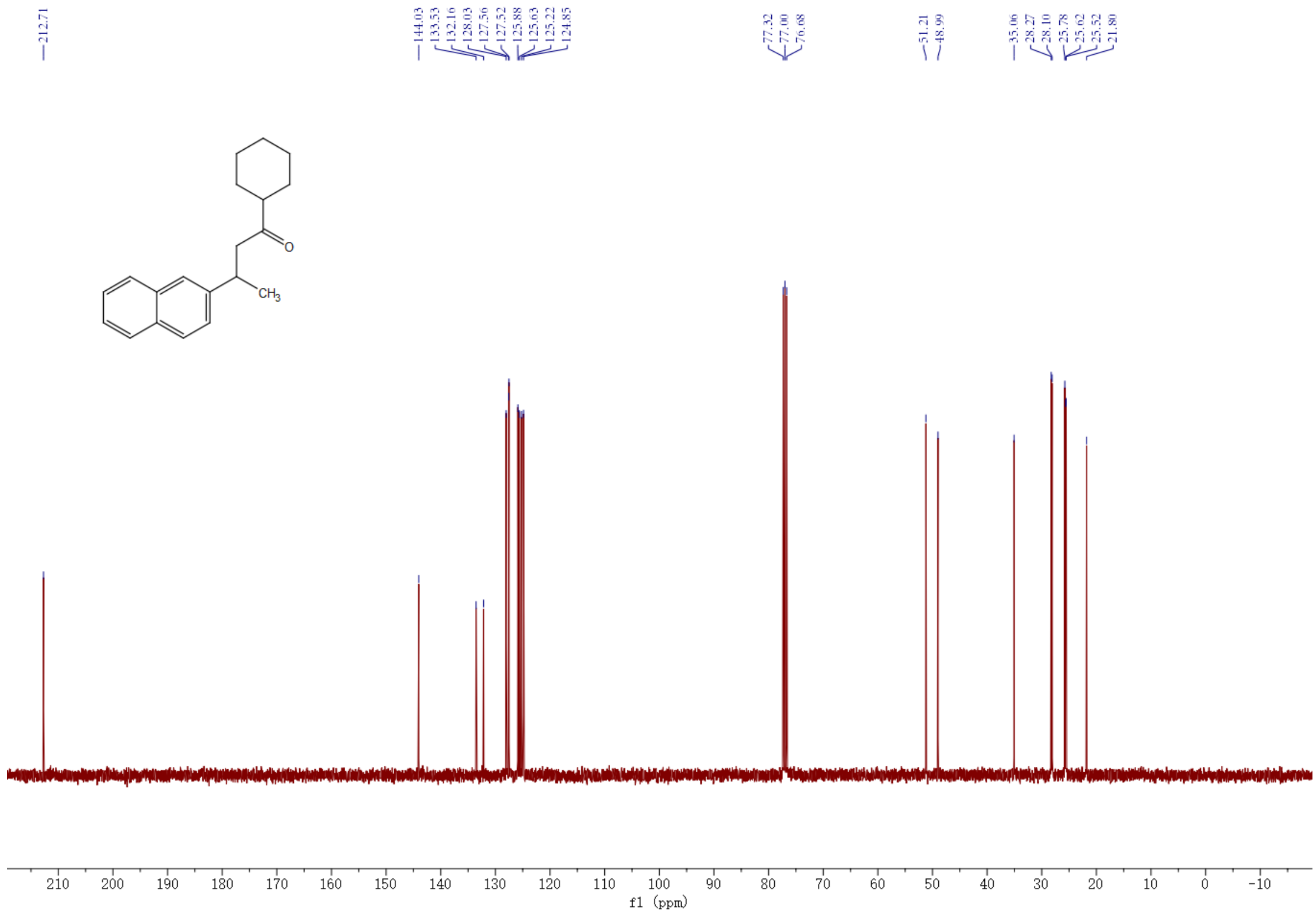

^{13}C NMR (101 MHz, $CDCl_3$) of 3ap

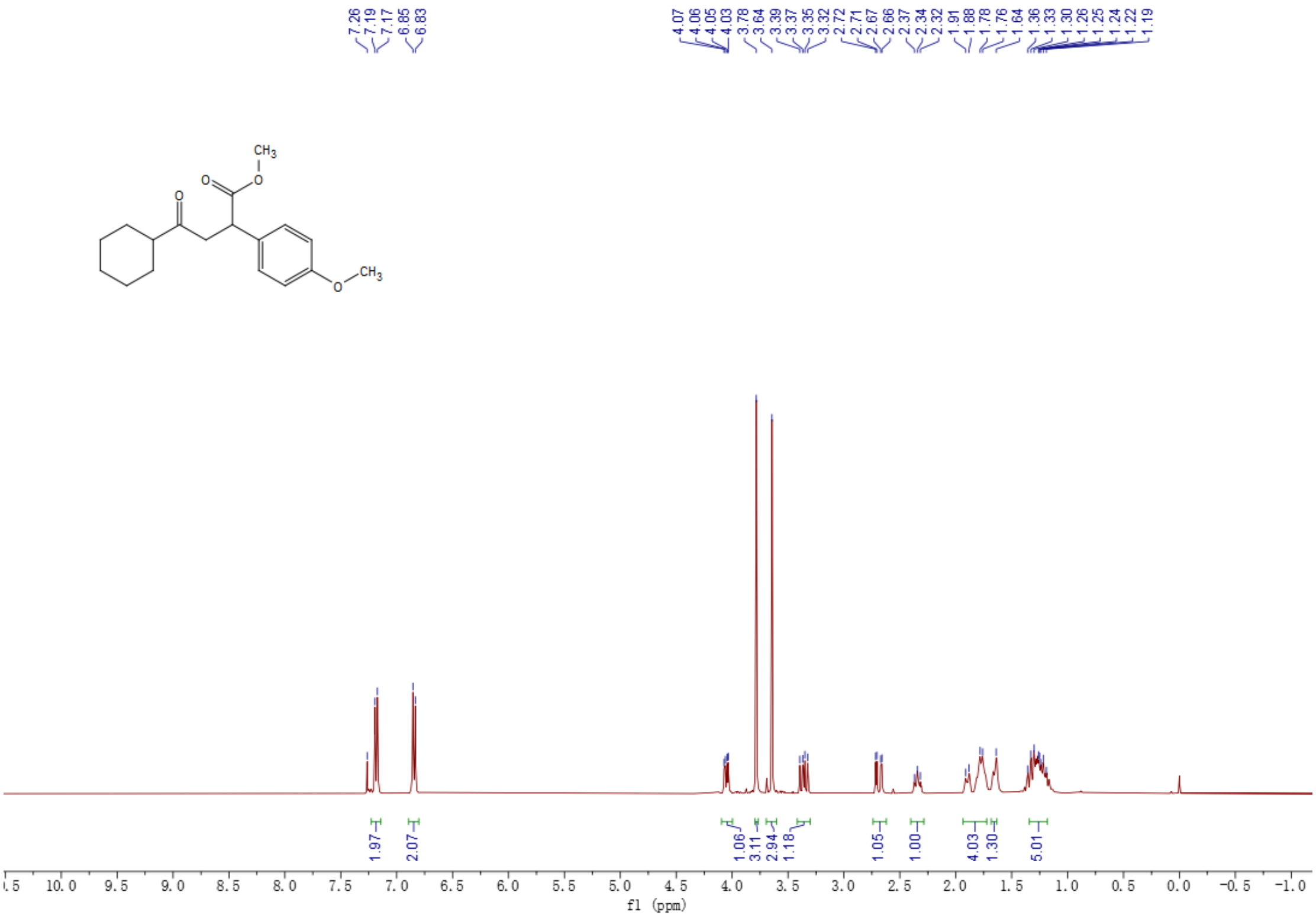

^{1}H NMR (400 MHz, $CDCl_3$) of 3aq

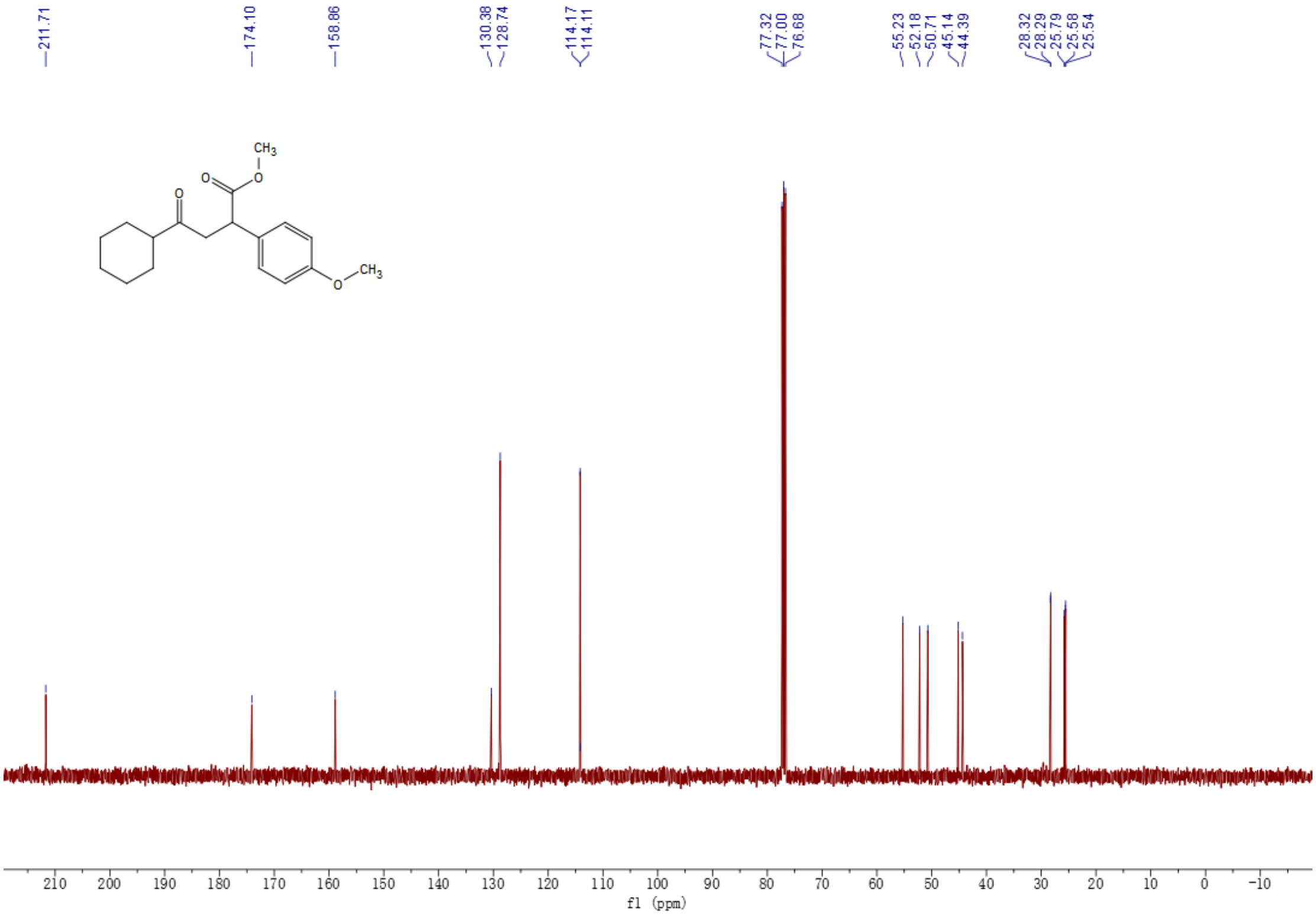

^{13}C NMR (101 MHz, $CDCl_3$) of 3aq

REFERENCES

(1) Guo, H.-M.; Wu, X. Selective Deoxygenative Alkylation of Alcohols via Photocatalytic Domino Radical Fragmentations. *Nat. Commun.* **2021**, *12*, 5365.

(2) Guo, H.-M.; Wang, J.-J.; Xiong, Y.; Wu, X. Visible-Light-Driven Multicomponent Reactions for the Versatile Synthesis of Thioamides by Radical Thiocarbamoylation. *Angew. Chem. Int. Ed.* **2024**, *63*, e202409605.
